# Supplementary material for: Amynthas corticis genome reveals molecular mechanisms behind global distribution
Source: Commun Biol. 2021 Jan 29;4:135. doi: 10.1038/s42003-021-01659-4 (PMC7846840; doi:10.1038/s42003-021-01659-4)
Supplement: Supplementary file 2 — Supplementary Information [file 42003_2021_1659_MOESM2_ESM.pdf]

Amynthas corticis genome reveals molecular mechanisms behind global distribution

Xing Wang[1,2,†]*, Yi Zhang[3,†], Yufeng Zhang[4,†], Mingming Kang[5,†], Yuanbo Li[1,2], Samuel W James[6], Yang Yang[7], Yanmeng Bi[8], Hao Jiang[1], Yi Zhao[1]*, Zhenjun Sun[1,2]*

[1]College of Resources and Environmental Sciences, China Agricultural University, Beijing, China.

[2]Beijing Key Laboratory of Biodiversity and Organic Farming, Beijing, China.

[3]School of Environmental Science & Engineering, Southern University of Science and Technology, Shenzhen, Guangdong, China.

[4]Hebei Key Laboratory of Animal Diversity, Langfang Normal University, Langfang, Hebei, China.

[5]College of Biological Sciences, China Agricultural University, Beijing,China.

[6]Maharishi University of Management, Fairfield, Iowa, USA.

[7]Beijing Gencode Diagnostics Laboratory, Beijing, China.

[8]School of Environmental and Municipal Engineering, Tianjin Chengjian University, Tianjin, China.

[†]These authors contributed equally: Xing Wang, Yi Zhang, Yufeng Zhang, Mingming Kang.

* Correspondence to: College of Resources and Environmental Sciences, China Agricultural University, Beijing 100193, China

E-mail address: swwangxing@cau.edu.cn (X. Wang); zhaoyis@163.com (Y. Zhao); sun108@cau.edu.cn (Z.J. Sun).

Supplementary figures

Supplementary Fig. 1: Morphological and anatomical characters of earthworm *Amynthas corticis*.

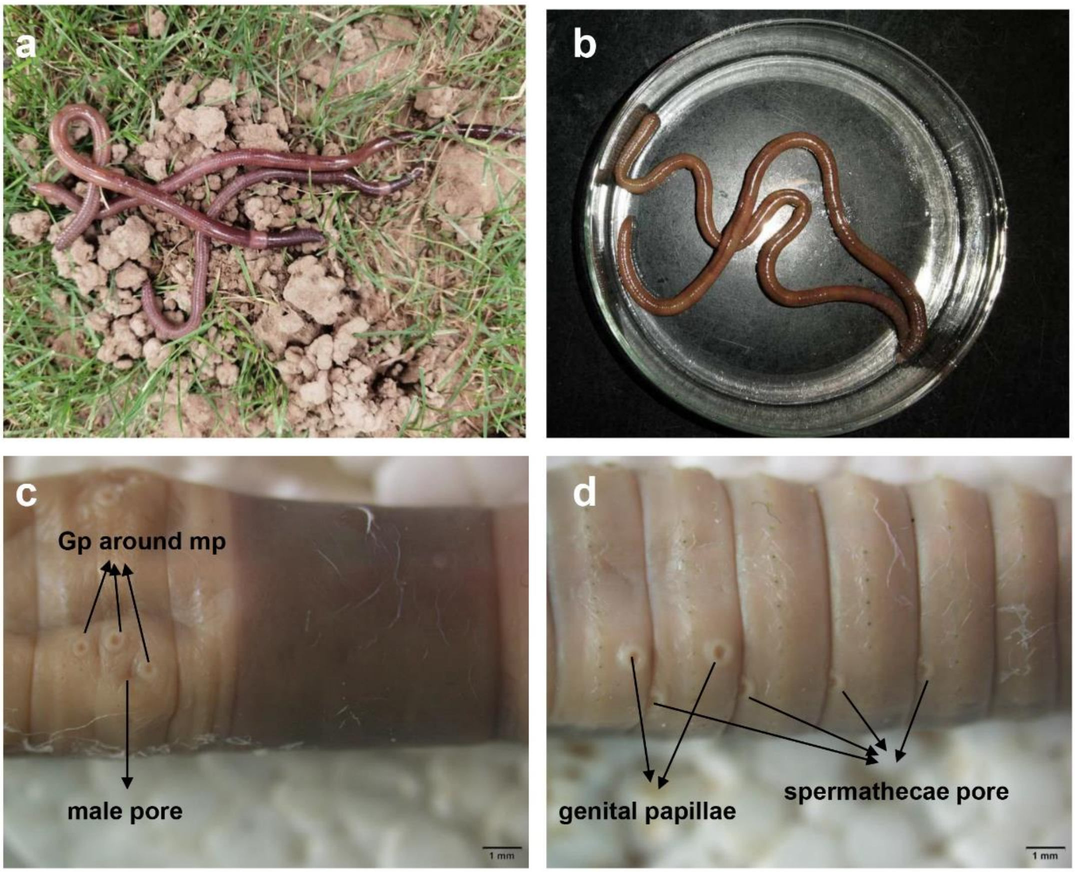

a, *A. corticis* in the wild. **b,** *A. corticis* in the lab. **c,** Three small and circular genital papillae are present around each male pore of *A. corticis*. **d,** Four pairs of spermathecae locate in venter of intersegments (5/6, 6/7, 7/8 and 8/9) of *A. corticis*.

Supplementary Fig. 2: COI barcode phylogeny for *Amynthas*.

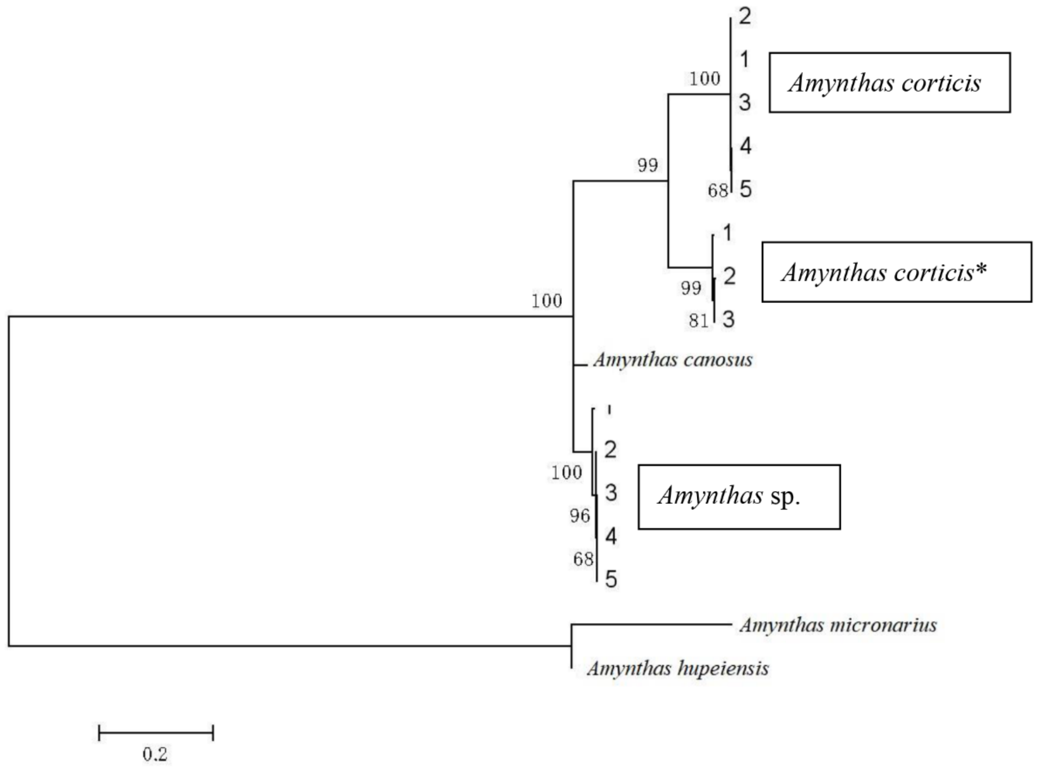

The gene tree was constructed based on COI barcode sequences of *A. corticis* as well as other earthworms in the genus of *Amynthas*. The number labeled at tips of the gene tree meant different earthworm individuals. COI barcode sequences were aligned via ClustalW. The gene tree was constructed via MEGA v.6.0 with maximum likelihood (ML) algorithm. Branch support values were calculated through bootstrap testing with 1000 replications. * represents the COI barcode extracted from the *A. corticis* genome discussed in this paper, others represent COI from GenBank.

Supplementary Fig. 3: Interaction intensities of scaffolds.

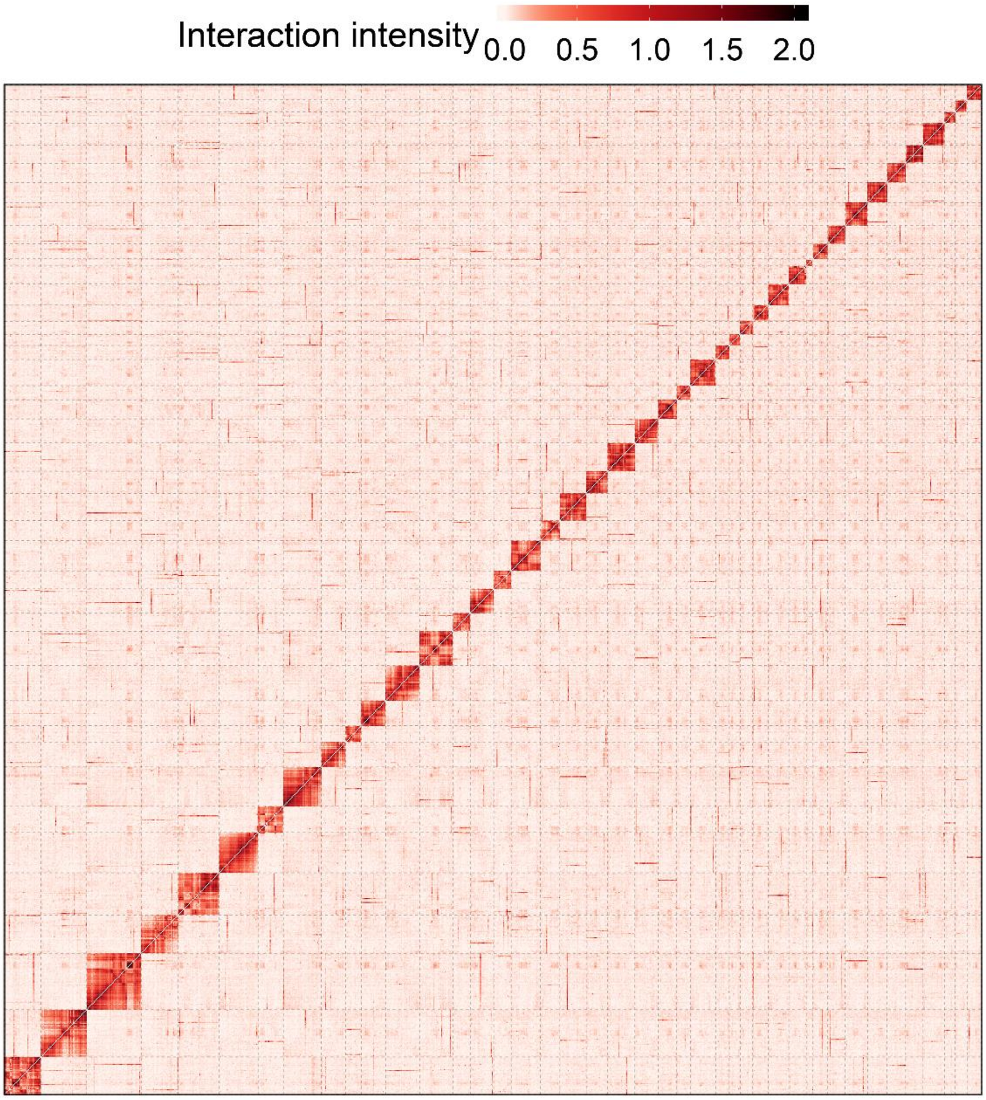

After assembly of contigs using PacBio SMRT long reads and polishing using paired-end reads sequenced from short-insert libraries, the genome of *Amynthas corticis* was scaffolding using Hi-C sequencing data ~~(Supplementary Information S5)~~. The heatmap shows interaction intensities calculated based on the mapping of Hi-C sequencing data between all pairs of the longest 42 scaffolds, in which each row and each column represents one scaffold, respectively. The darker the color is, the stronger the interaction intensity is. Interaction intensities within the same scaffold are much stronger than ones between different scaffolds, indicating correct placing of contigs on scaffolds.

Supplementary Fig. 4: The number distribution of expanded gene families.

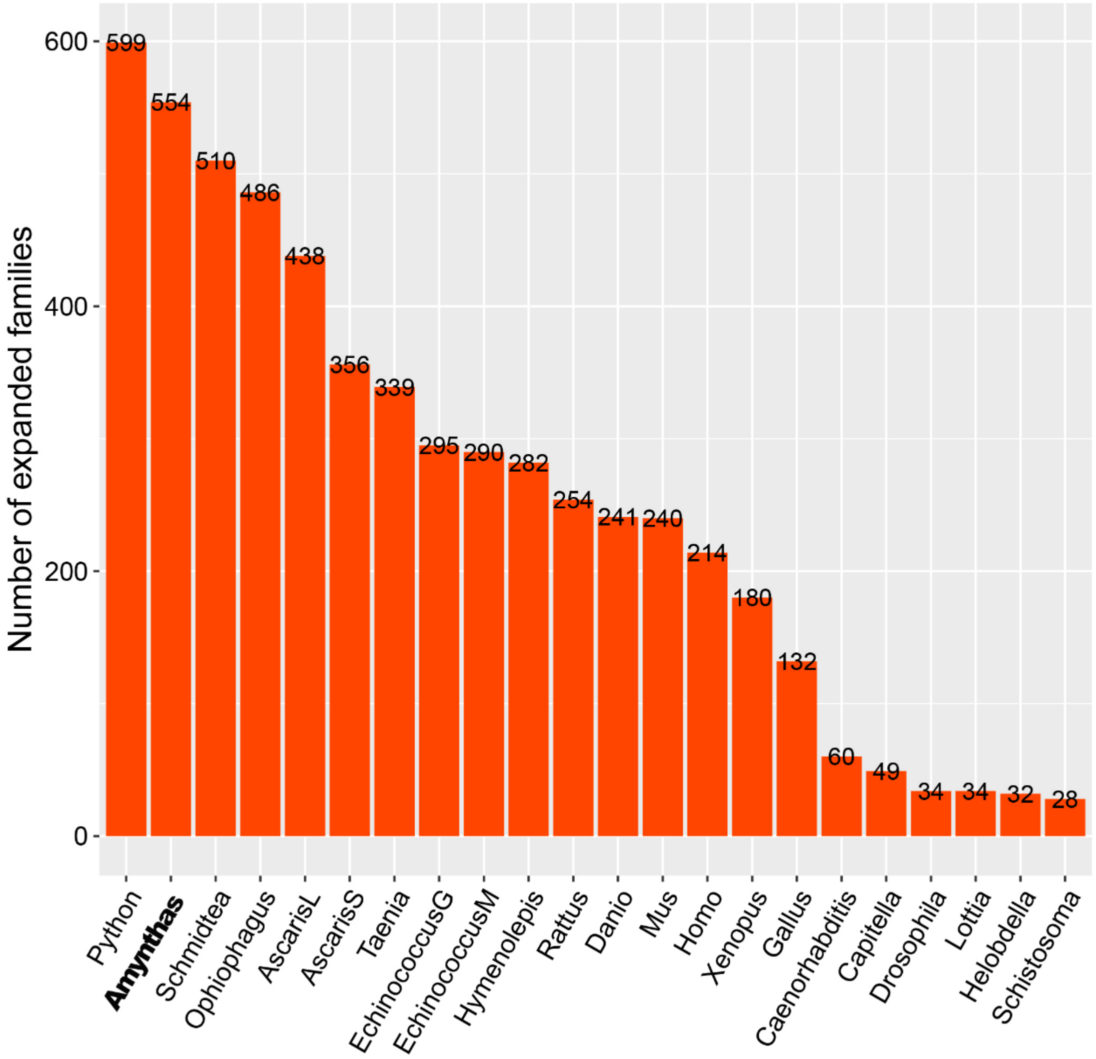

Gene families with significantly accelerated rates of gene duplication and loss indicated by CAFE were selected to do this statistic. For each species, if the count of gene members in a family is larger than that of the MRCA of all species, this family was labeled as expanded for the species~~ (Supplementary Information S15)~~. *Amynthas corticis* is represented by earthworm in this figure, and it has the second most expanded families, which is a distinguished feature compared with other two annelids. In this figure, Earthworm: *Amynthas corticis*, Helobdella: *Helobdella robusta*, Capitella: *Capitella teleta*, EchinococcusG: *Echinococcus granulosus*, EchinococcusM: *Echinococcus multilocularis*, Hymenolepis: *Hymenolepis microstoma*, Taenia: *Taenia solium*, Schistosoma: *Schistosoma mansoni*, Schmidtea: *Schmidtea mediterranea*, AscarisL: *Ascaris lumbricoides*, AscarisS: *Ascaris suum*, Caenorhabditis:

Caenorhabditis elegans, Lottia: *Lottia gigantea*, Drosophila: *Drosophila melanogaster*, Danio: *Danio rerio*, Xenopus: *Xenopus tropicalis*, Ophiophagus: *Ophiophagus hannah*, Python: *Python bivittatus*, Gallus: *Gallus gallus*, Mus: *Mus musculus*, Rattus: *Rattus norvegicus* and Homo: *Homo sapiens*.

Supplementary Fig. 5: The number distribution of contracted gene families.

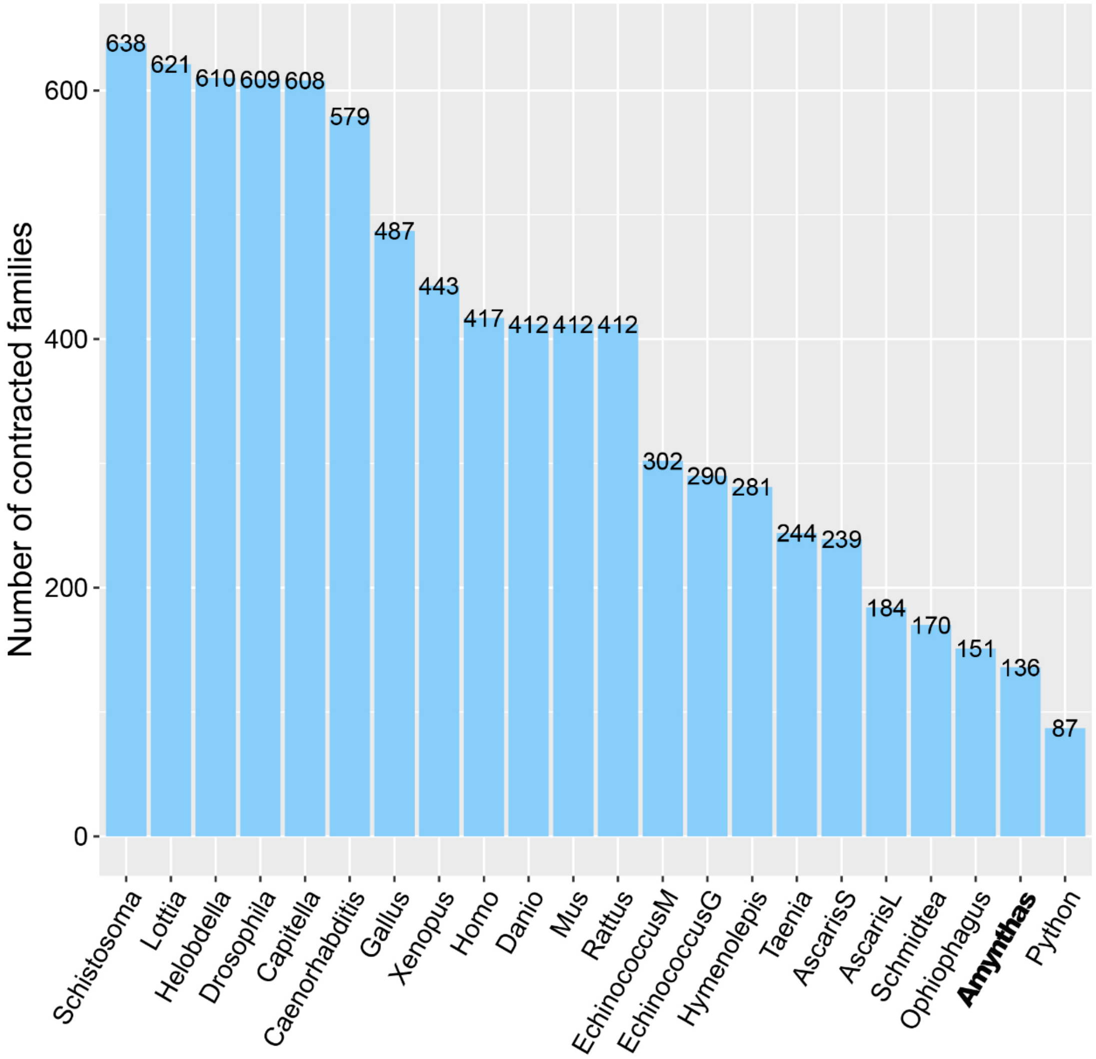

Gene families with significantly accelerated evolutionary rate indicated by CAFE were selected to do this statistics. For each species, if the count of gene members in a family is smaller than that of the MRCA of all species, this family was labeled as contracted for the species. *Amynthas corticis* is represented by earthworm in this figure, and it has the second fewest contracted families, which is a distinguished feature compared with other two annelids. Names of species in this figure are the same as Supplementary Fig. 4.

Supplementary Fig. 6: The number distribution of stable gene families.

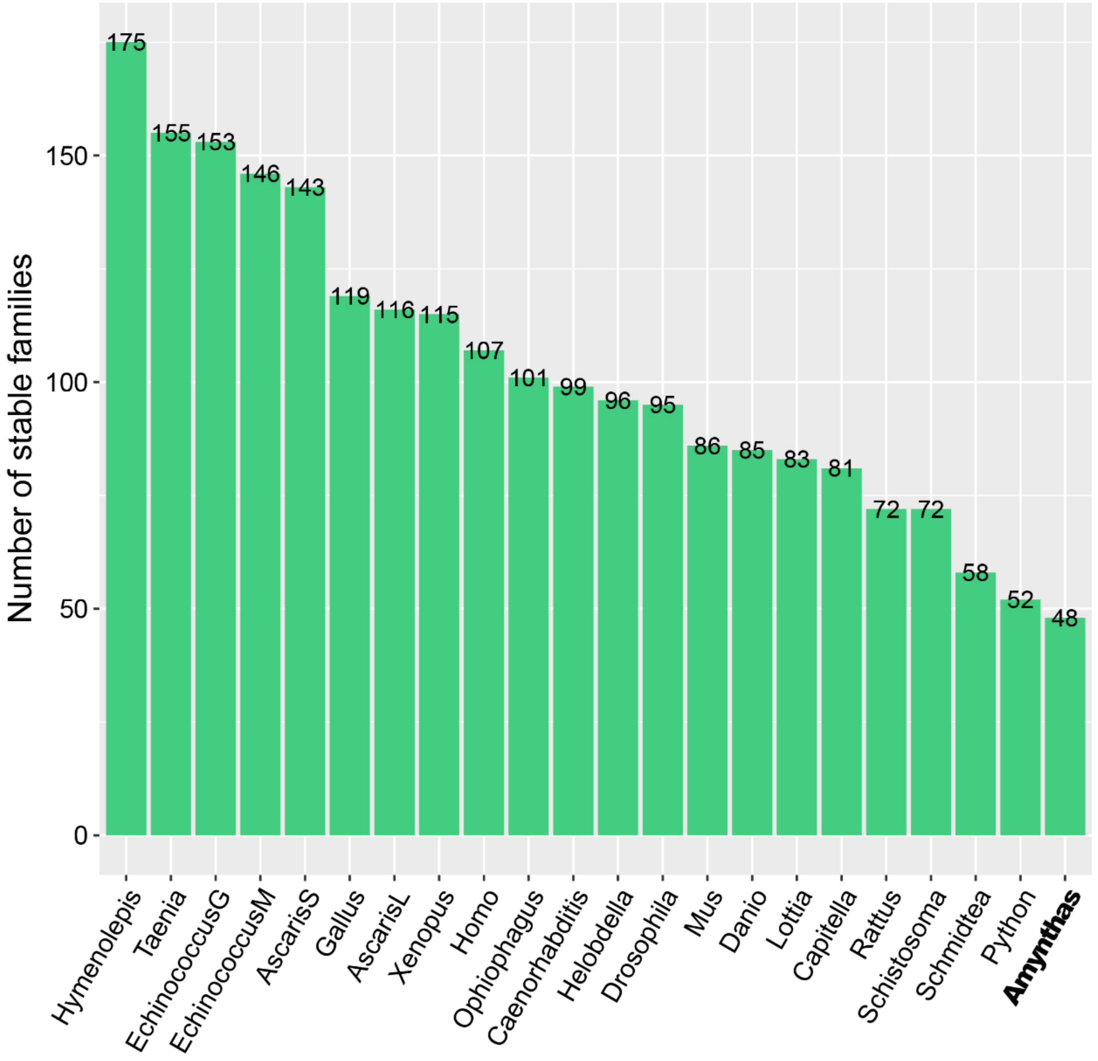

Gene families with significantly accelerated evolutionary rate indicated by CAFE were selected to do this statistics. For each species, if the count of gene members in a family is equal to that of the MRCA of all species, this family was labeled as stable for the species. *Amynthas corticis* is represented by earthworm in this figure, and it has the fewest stable families, suggesting evolutionary changes of gene families in *A. corticis* have been dramatic. Names of species in this figure are the same as Supplementary Fig 4.

Supplementary Fig. 7: Odds ratio of significantly enriched GO terms attributed to GOSlim of immune system process.

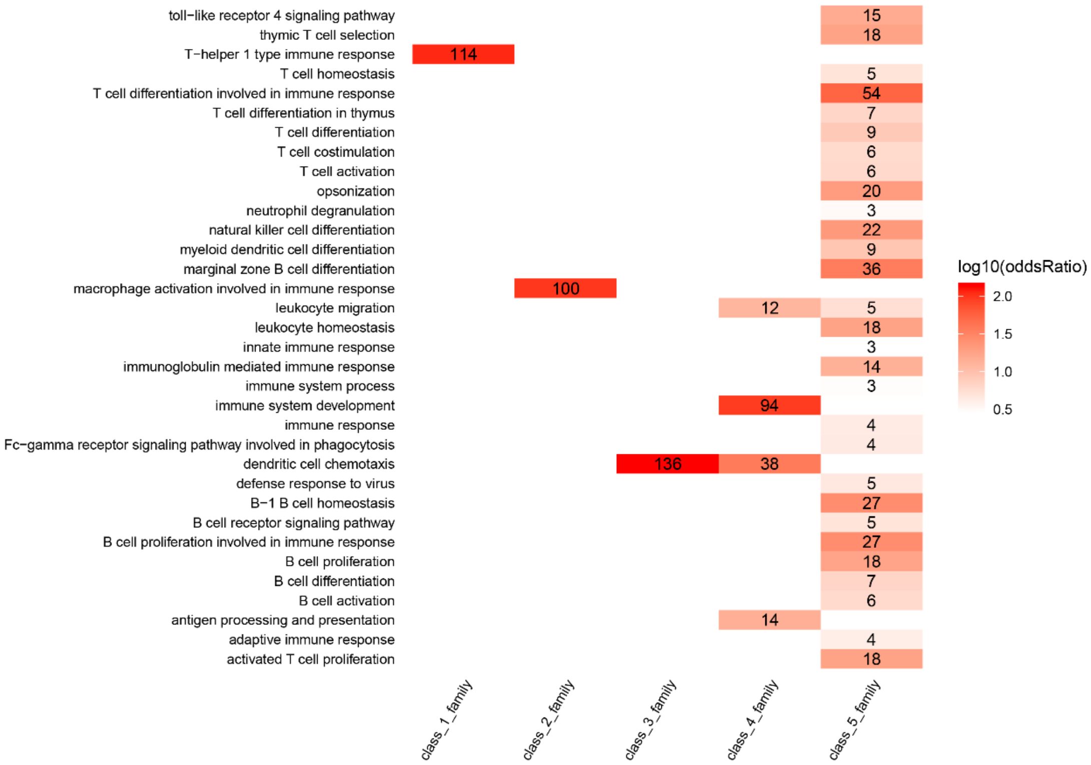

GO enrichment analysis was conducted for five subsets of gene families with positive variable loading in the first component. Odds ratio of significantly enriched GO terms attributed to GOSlim of immune system process was colored and labeled for each subset of gene families, in which the redder the color is, the higher the odds ratio is.

Supplementary Fig. 8: Odds ratio of significantly enriched GO terms attributed

to GOSlim of response to stress.

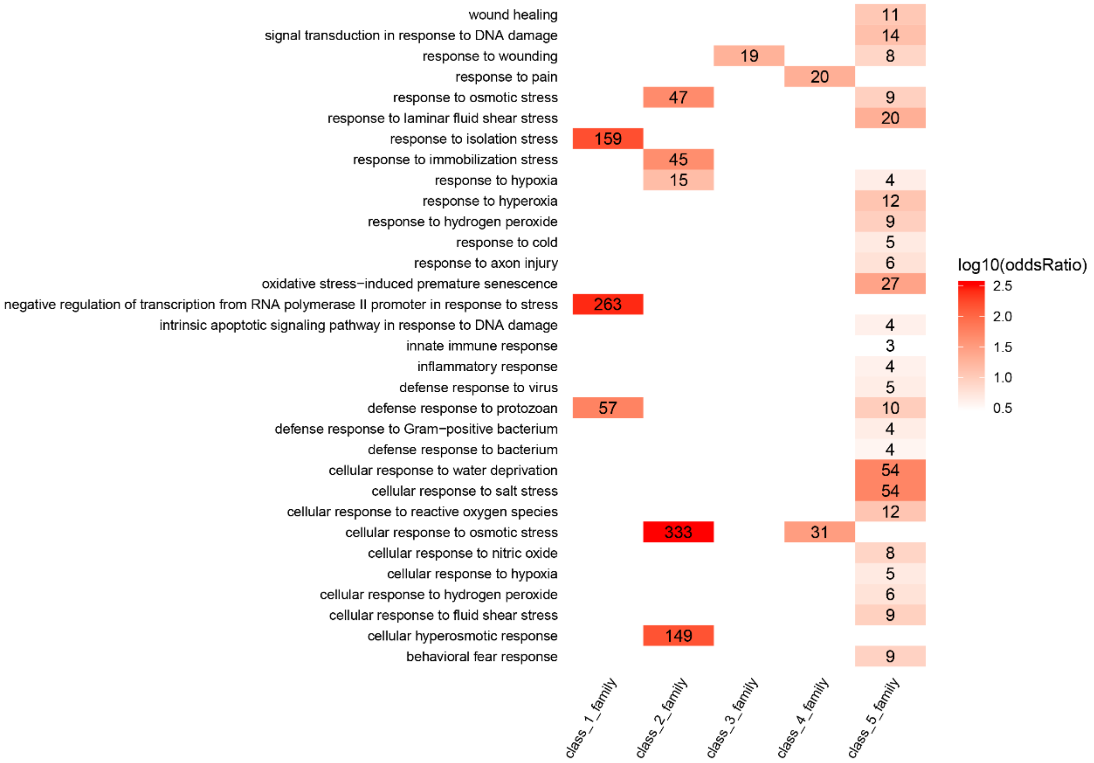

GO enrichment analysis was conducted for five subsets of gene families with positive variable loading in the first component. Odds ratio of significantly enriched GO terms attributed to GOSlim of response to stress was colored and labeled for each subset of gene families, in which the redder the color is, the higher the odds ratio is.

Supplementary Fig. 9: Odds ratio of significantly enriched GO terms attributed to GOSlim of homeostatic process.

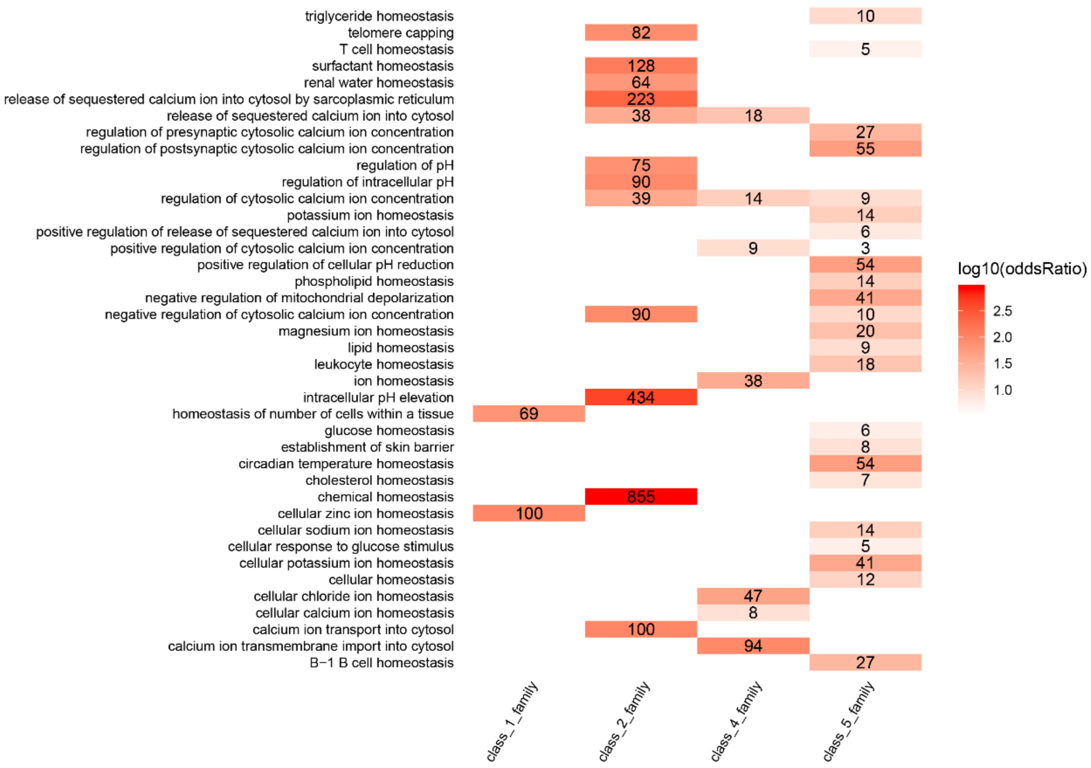

GO enrichment analysis was conducted for five subsets of gene families with positive variable loading in the first component. Odds ratio of significantly enriched GO terms attributed to GOSlim of homeostatic process was colored and labeled for each subset of gene families, in which the redder the color is, the higher the odds ratio is.

Supplementary Fig. 10: Odds ratio of significantly enriched GO terms attributed to GOSlim of anatomical structure development.

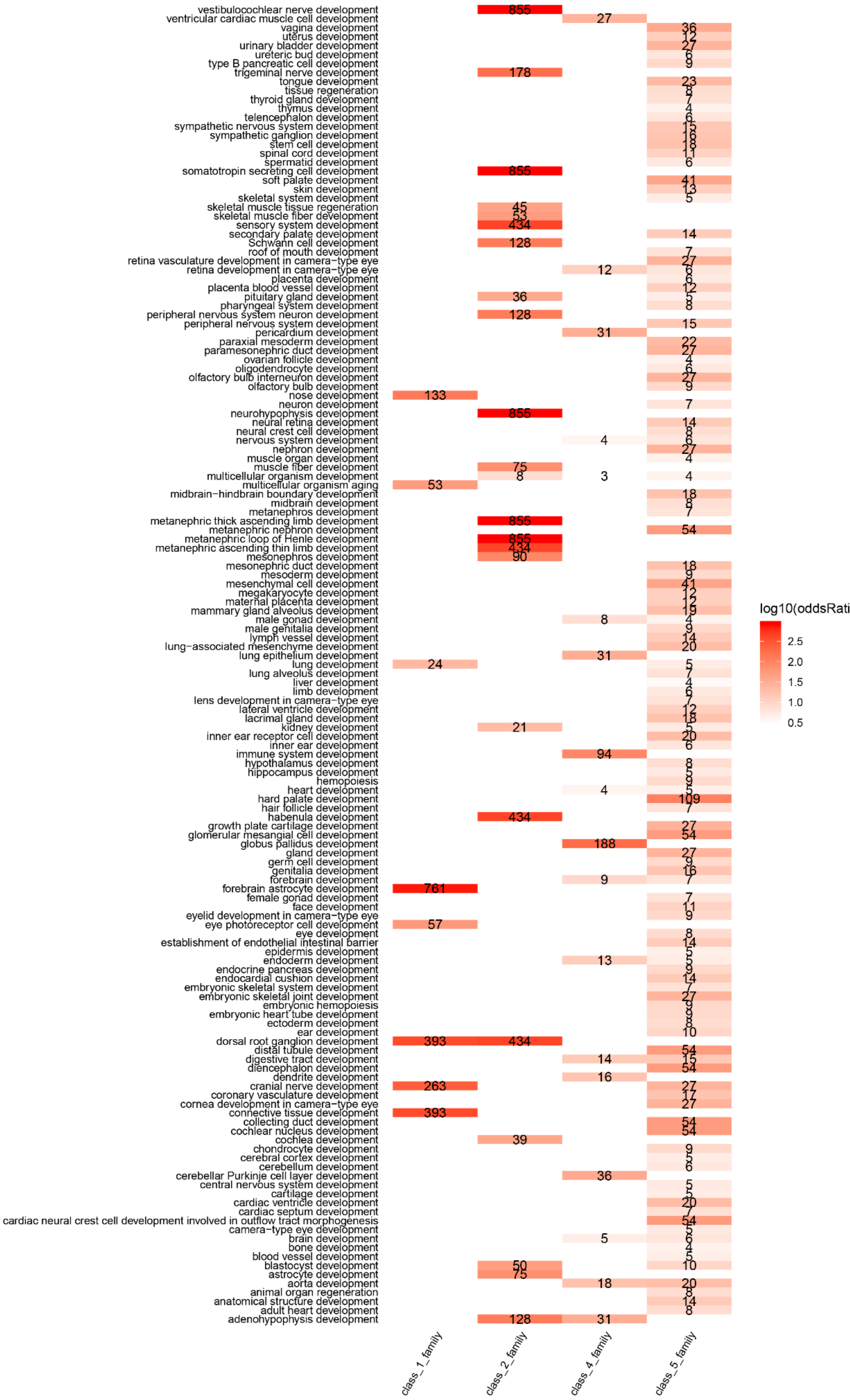

GO enrichment analysis was conducted for five subsets of gene families with positive variable loading in the first component. Odds ratio of significantly enriched GO terms attributed to GOSlim of anatomical structure development was colored and labeled for each subset of gene families, in which the redder the color is, the higher the odds ratio

is.

Supplementary Fig. 11: Odds ratio of significantly enriched GO terms attributed to GOSlim of signal transduction.

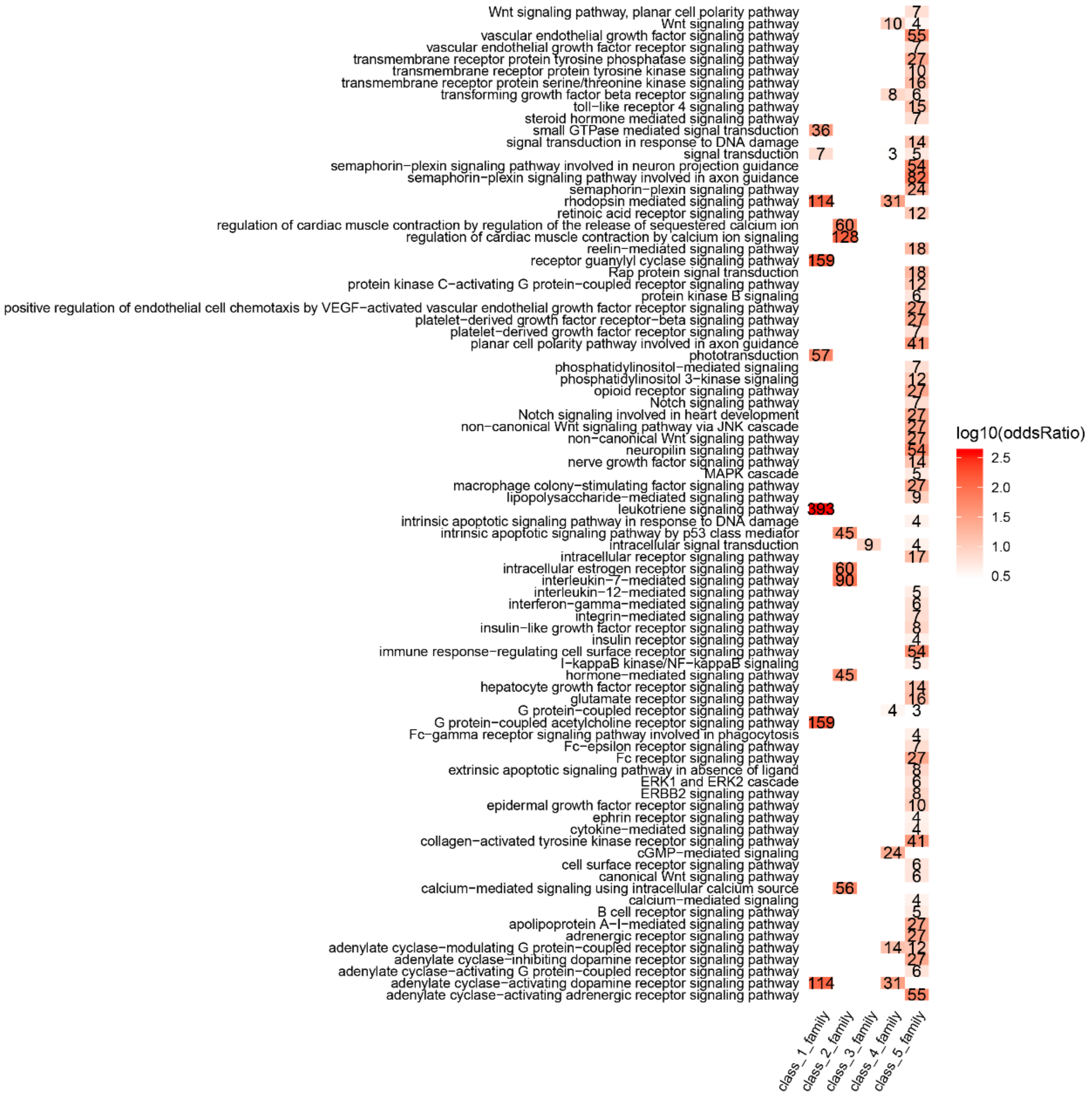

GO enrichment analysis was conducted for five subsets of gene families with positive variable loading in the first component. Odds ratio of significantly enriched GO terms attributed to GOSlim of signal transduction was colored and labeled for each subset of gene families, in which the redder the color is, the higher the odds ratio is.

Supplementary Fig. 12: Odds ratio of significantly enriched GO terms attributed to GOSlim of cell differentiation.

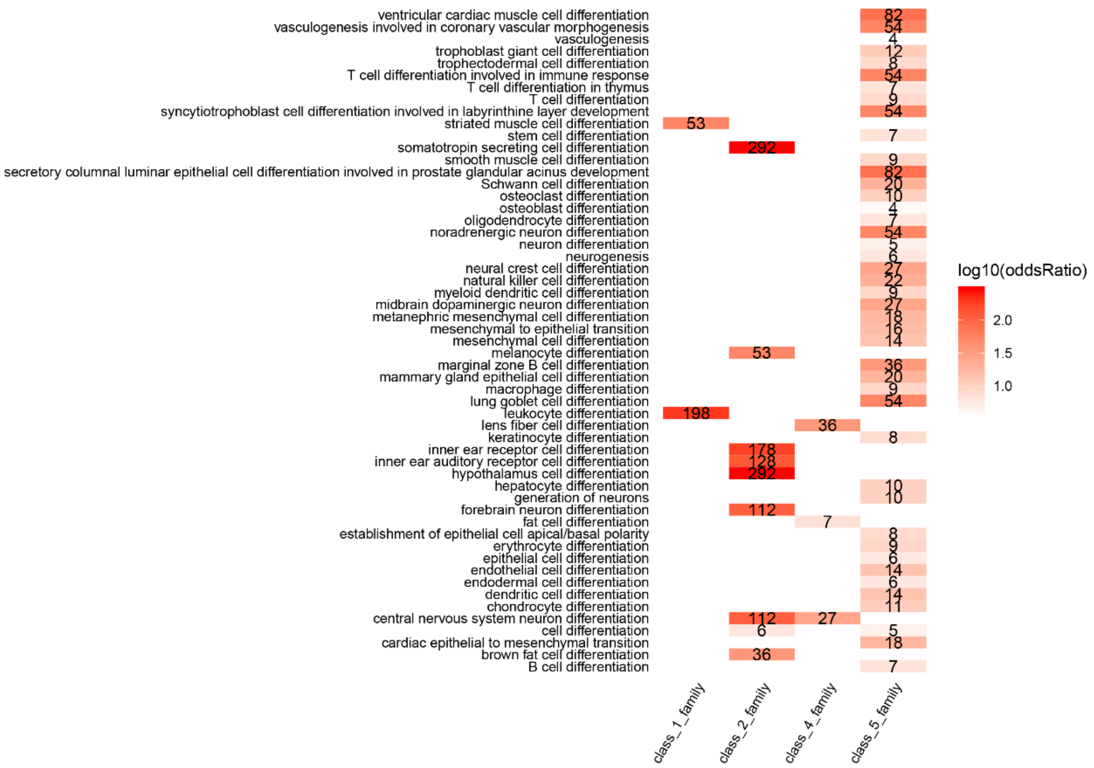

GO enrichment analysis was conducted for five subsets of gene families with positive variable loading in the first component. Odds ratio of significantly enriched GO terms attributed to GOSlim of cell differentiation was colored and labeled for each subset of gene families, in which the redder the color is, the higher the odds ratio is.

Supplementary Fig. 13: Genomic locations of orthologous hits of well determined earthworm defensive genes.

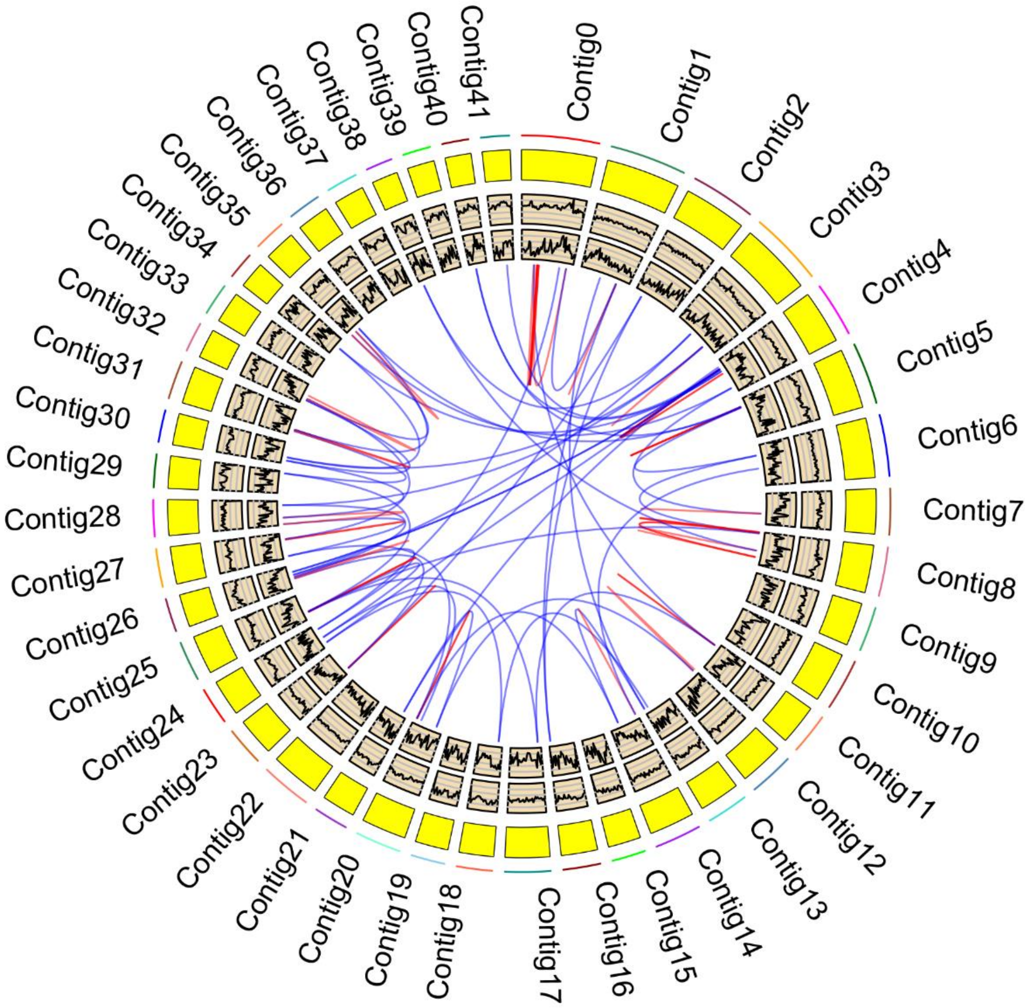

Genomics locations of 111 orthologous hits of 15 well determined earthworm defensive genes were plotted. The outermost ring represents the longest 42 scaffolds. Then, from outside to inside, two rings represent the density distribution of repeat sequences and SNPs. Density distributions of SNPs in genomic regions containing orthologous hits and other regions are significantly different. Lines in the center connect orthologous hits of the same well determined earthworm defensive gene, in which hits on the same chromosome are connected by red lines and hits on different chromosomes are connected by blue lines.

Supplementary Fig. 14: Expression of orthologous hits of well determined earthworm defensive genes.

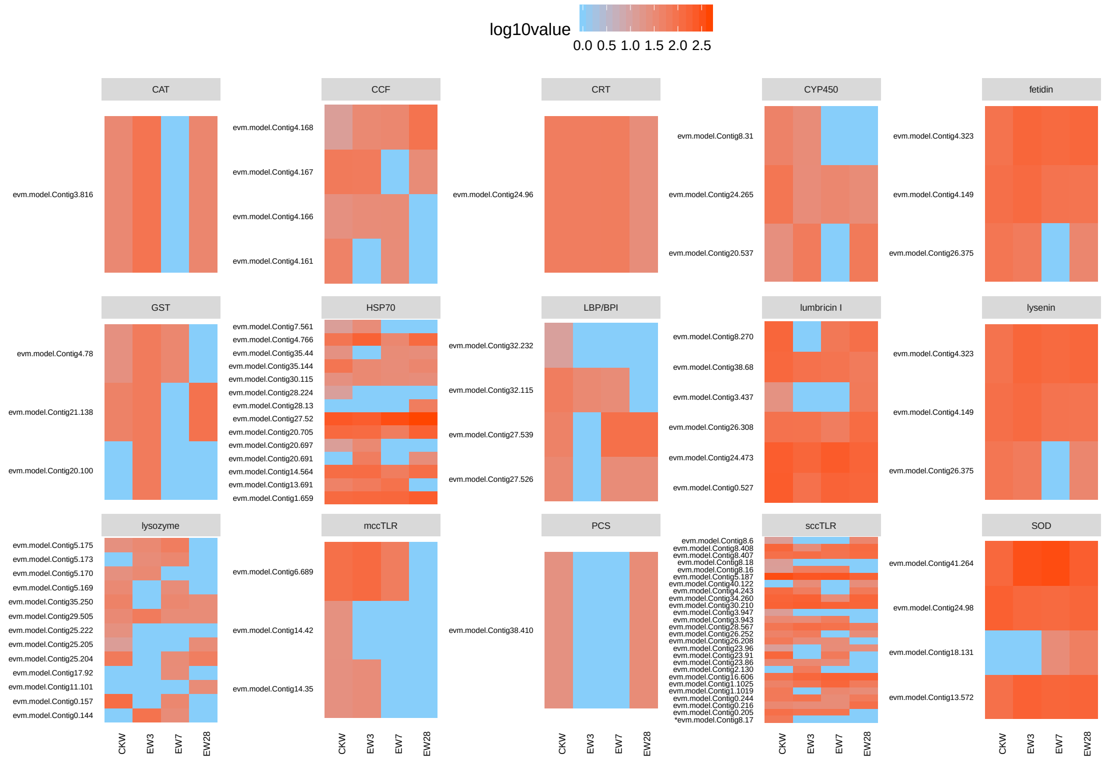

Expression of orthologous hits of well determined earthworm defensive genes were detected by protein mass spectrum of gut removed body along the time course, including before incubation (CKW) in infected soil with pathogenic *E. coli O157:H7*, on the third (EW3), seventh (EW7) and twenty-eighth (EW28) day after incubation. Each panel in this figure represents expression of orthologous hits of each well determined earthworm defensive gene, in which the name of defensive gene is labeled on the top and mean expression levels at different time points are colored from blue (low expression level) to red (high expression level). The name of orthologous hit with significantly varied expression identified in time series analysis was labeled with a star signal.

Supplementary Fig. 15: Proofs supporting that *Eisenia fetida* is diploid.

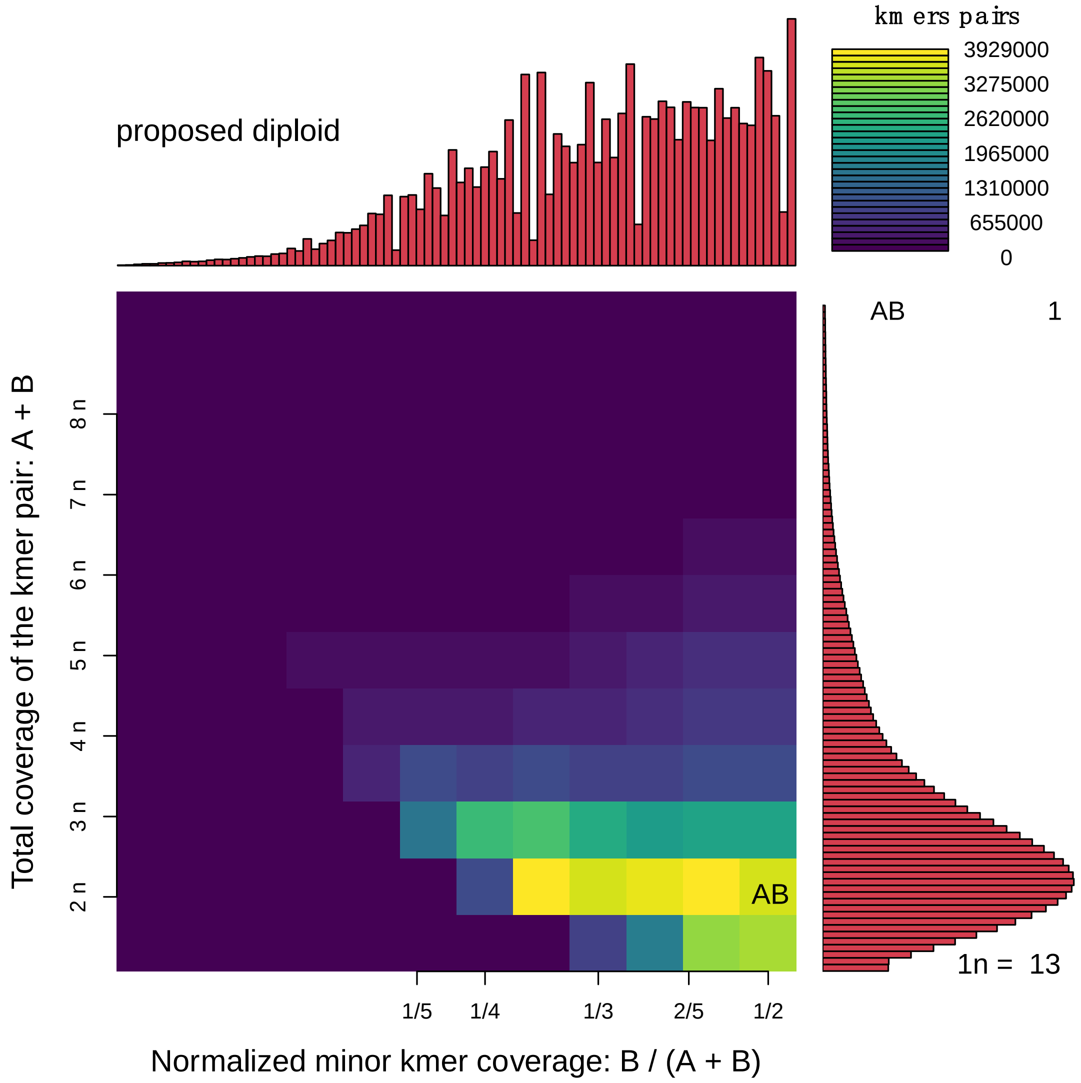

Heatmap for coverage pattern of heterozygous k-mer pairs, in which X axis indicates the normalized minor k-mer coverage, while Y axis indicates the total k-mer pairs coverage. The location on the heatmap colored by bright yellow represents that two are the most k-mer pairs supporting the normalized minor k-mer coverage of 1/2 and the total k-mer pairs coverage of 2n.

Supplementary Fig. 16: Odds ratio distribution of enriched functions related to immune system.

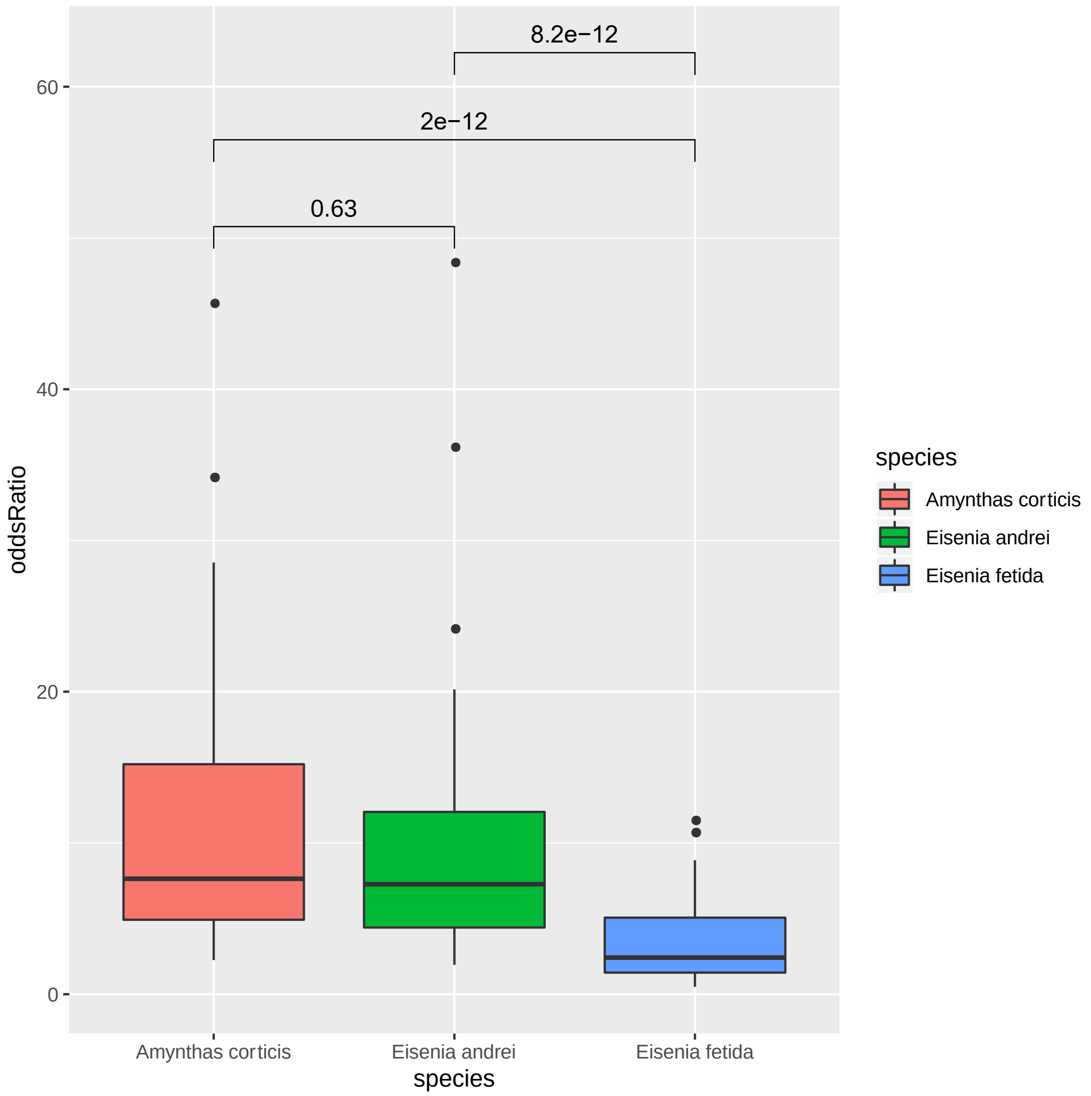

Odds ratio distribution of enriched functions related to immune system process among genomes of *A. corticis*, *Eisenia fetida* and *Eisenia andrei*, in which Wilcoxon rank sum test was adopted to do the comparing analysis.

Supplementary Fig. 17: Odds ratio distribution of enriched functions related to response to stress.

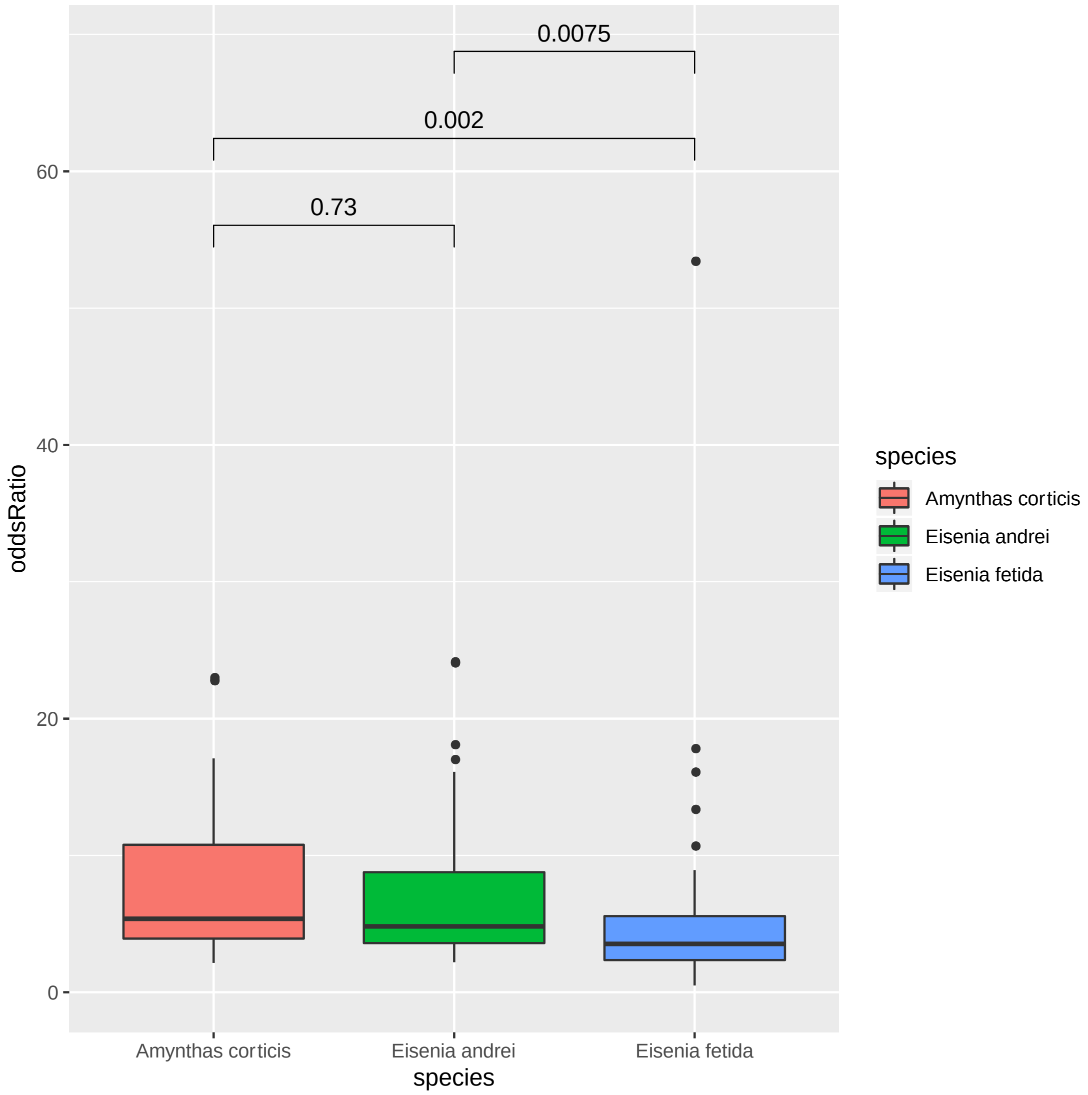

Odds ratio distribution of enriched functions related to response to stress among genomes of *A. corticis*, *Eisenia fetida* and *Eisenia andrei*, in which Wilcoxon rank sum test was adopted to do the comparing analysis.

Supplementary Fig. 18: Odds ratio distribution of enriched functions related to homeostatic process.

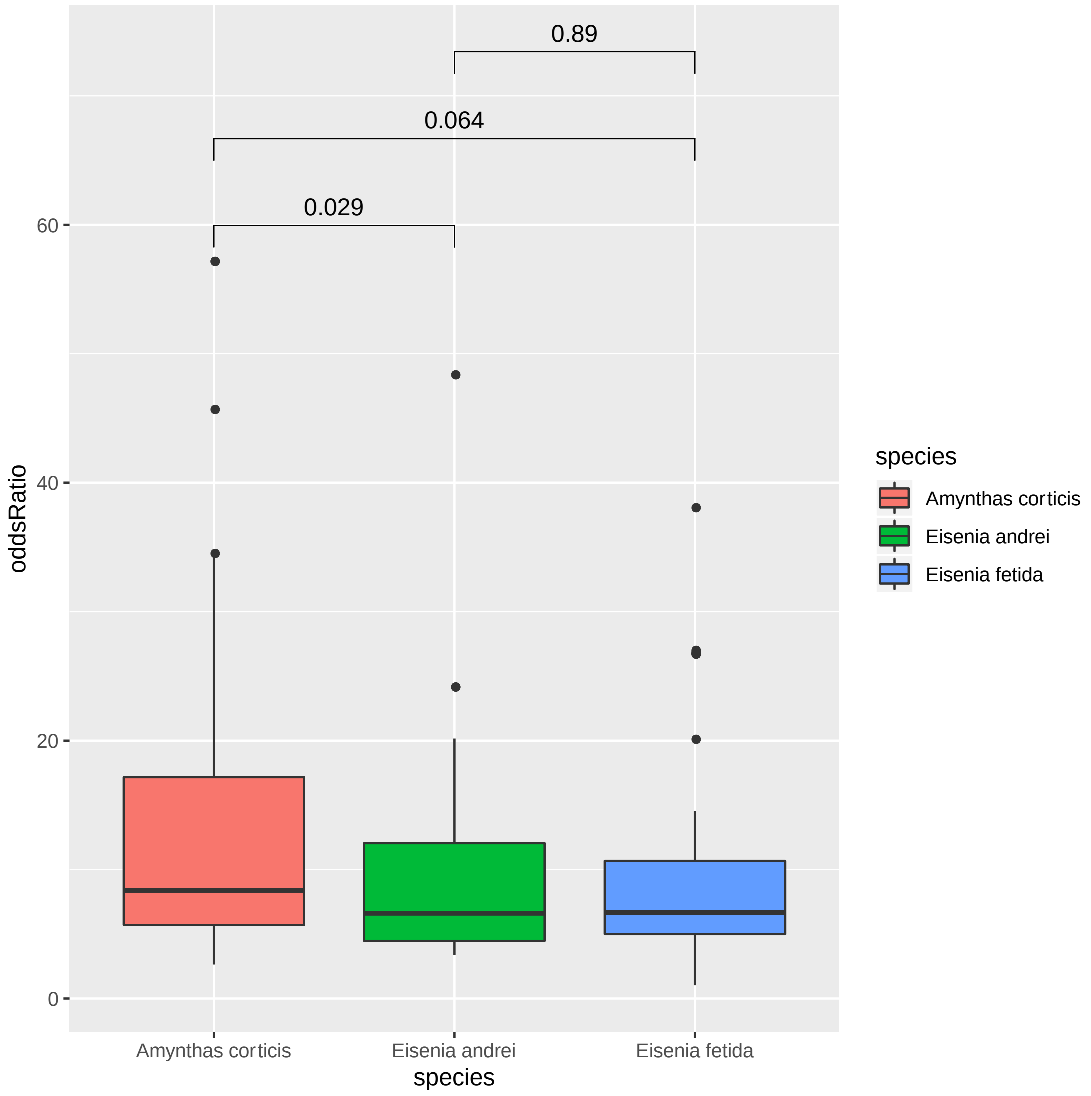

Odds ratio distribution of enriched functions related to homeostatic process among genomes of *A. corticis*, *Eisenia fetida* and *Eisenia andrei*, in which Wilcoxon rank sum test was adopted to do the comparing analysis.
