# Supplementary material for: Amynthas corticis genome reveals molecular mechanisms behind global distribution
Source: Commun Biol. 2021 Jan 29;4:135. doi: 10.1038/s42003-021-01659-4 (PMC7846840; doi:10.1038/s42003-021-01659-4)
Supplement: Supplementary file 3 — Description of Additional Supplementary Files [file 42003_2021_1659_MOESM3_ESM.pdf]

Description of Additional Supplementary Files

File name: Supplementary data file 1

Description: *Amynthas corticis* genes annotated by TreeFam family and HGNC gene list.

File name: Supplementary data file 2

Description: Duplicated regions in *Amynthas corticis* genome identified by MCScanX.

File name: Supplementary data file 3

Description: Density distribution of repeats, noncoding genes, coding genes and SNPs in *Amynthas corticis* genome.

File name: Supplementary data file 4

Description: Spearman's rank correlation coefficients of gene member count in families with accelerated rates of gene duplication and loss between species.

File name: Supplementary data file 5

Description: PCA coordinate calculated based on gene member count in families with accelerated rates of gene duplication and loss.

File name: Supplementary data file 6

Description: GO enrichment analysis for 5 subsets of gene families.

File name: Supplementary data file 7

Description: GO enrichment analysis for the fifth subset of gene families in *Amynthas corticis*, *Eisenia fetida* and *Eisenia andrei*.

File name: Supplementary data file 8

Description: Mass spectrum value of *Amynthas corticis* proteins with varied expression along the time course before and after incubation in an artificial soil infected with pathogenic *Escherichia coli* O157:H7.

File name: Supplementary data file 9

Description: GO enrichment analysis for *Amynthas corticis* proteins with varied expression along the time course before and after incubation in an artificial soil infected with pathogenic *Escherichia coli* O157:H7.

File name: Supplementary data file 10

Description: 16S rDNA abundance of microorganisms in *Amynthas corticis* gut with varied abundance along the time course before and after incubation in an artificial soil infected with pathogenic *Escherichia coli* O157:H7.

File name: Supplementary data file 11

Description: COG annotation for microorganisms in *Amynthas corticis* gut with varied abundance along the time course before and after incubation in an artificial soil infected with pathogenic *Escherichia coli* O157:H7.

File name: Supplementary data file 12

Description: Nodes in four motifs of interaction network between genome and gut microbiome of *Amynthas corticis*.

File name: Supplementary data file 13

Description: Features of microorganisms involved in interaction network between genome and gut microbiome of *Amynthas corticis*.

File name: Supplementary data file 14

Description: Information of orthologous hits of well determined defensive genes in *Amynthas corticis* genome.

File name: Supplementary data file 15

Description: Protein mass spectrum value of orthologous hits of well determined defensive genes in *Amynthas corticis* genome.

File name: Supplementary data file 16

Description: Sources of species proteins involved in evolutionary analysis in this study.
