# Supplementary material for: Amynthas corticis genome reveals molecular mechanisms behind global distribution
Source: Commun Biol. 2021 Jan 29;4:135. doi: 10.1038/s42003-021-01659-4 (PMC7846840; doi:10.1038/s42003-021-01659-4)
Supplement: Supplementary file 5 — Supplementary Data 2 [file 42003_2021_1659_MOESM5_ESM.pdf]

```
############### Parameters ###############
# MATCH_SCORE: 50
# MATCH_SIZE: 5
# GAP_PENALTY: -1
# OVERLAP_WINDOW: 5
# E_VALUE: 1e-05
# MAX GAPS: 25
############### Statistics ###############
# Number of collinear genes: 5589, Percentage: 19.10
# Number of all genes: 29256
##########################################
## Alignment 0: score=628.0 e_value=4.5e-38 N=14 ew0&ew0 plus
  0-  0:	evm.model.Contig0.90	evm.model.Contig0.701	 6e-109
  0-  1:	evm.model.Contig0.91	evm.model.Contig0.702	  4e-36
  0-  2:	evm.model.Contig0.95	evm.model.Contig0.705	 1e-122
  0-  3:	evm.model.Contig0.99	evm.model.Contig0.712	 5e-139
  0-  4:	evm.model.Contig0.100	evm.model.Contig0.714	 8e-124
  0-  5:	evm.model.Contig0.113	evm.model.Contig0.726	  6e-58
  0-  6:	evm.model.Contig0.116	evm.model.Contig0.734	      0
  0-  7:	evm.model.Contig0.117	evm.model.Contig0.735	      0
  0-  8:	evm.model.Contig0.131	evm.model.Contig0.744	  6e-68
  0-  9:	evm.model.Contig0.132	evm.model.Contig0.745	  1e-98
  0- 10:	evm.model.Contig0.133	evm.model.Contig0.767	      0
  0- 11:	evm.model.Contig0.134	evm.model.Contig0.769	      0
  0- 12:	evm.model.Contig0.136	evm.model.Contig0.770	      0
  0- 13:	evm.model.Contig0.142	evm.model.Contig0.778	  1e-61
## Alignment 1: score=278.0 e_value=1.3e-08 N=6 ew0&ew0 plus
  1-  0:	evm.model.Contig0.14	evm.model.Contig0.420	  7e-65
  1-  1:	evm.model.Contig0.19	evm.model.Contig0.425	  9e-51
  1-  2:	evm.model.Contig0.24	evm.model.Contig0.431	  6e-60
  1-  3:	evm.model.Contig0.25	evm.model.Contig0.433	  6e-63
  1-  4:	evm.model.Contig0.29	evm.model.Contig0.437	  4e-50
  1-  5:	evm.model.Contig0.39	evm.model.Contig0.440	 2e-150
## Alignment 2: score=266.0 e_value=7.2e-12 N=7 ew0&ew0 plus
  2-  0:	evm.model.Contig0.99	evm.model.Contig0.686	  2e-46
  2-  1:	evm.model.Contig0.104	evm.model.Contig0.696	  6e-32
  2-  2:	evm.model.Contig0.114	evm.model.Contig0.722	  7e-74
  2-  3:	evm.model.Contig0.132	evm.model.Contig0.732	  6e-45
  2-  4:	evm.model.Contig0.133	evm.model.Contig0.735	 2e-138
  2-  5:	evm.model.Contig0.143	evm.model.Contig0.759	  2e-61
  2-  6:	evm.model.Contig0.152	evm.model.Contig0.761	  2e-48
## Alignment 3: score=455.0 e_value=8.4e-21 N=10 ew0&ew0 minus
  3-  0:	evm.model.Contig0.19	evm.model.Contig0.369	  2e-56
  3-  1:	evm.model.Contig0.21	evm.model.Contig0.366	  1e-57
  3-  2:	evm.model.Contig0.23	evm.model.Contig0.365	  6e-74
  3-  3:	evm.model.Contig0.25	evm.model.Contig0.360	  7e-18
  3-  4:	evm.model.Contig0.26	evm.model.Contig0.358	 9e-118
  3-  5:	evm.model.Contig0.32	evm.model.Contig0.351	 1e-118
  3-  6:	evm.model.Contig0.42	evm.model.Contig0.340	  2e-56
  3-  7:	evm.model.Contig0.46	evm.model.Contig0.337	 1e-116
  3-  8:	evm.model.Contig0.49	evm.model.Contig0.336	  3e-67
  3-  9:	evm.model.Contig0.59	evm.model.Contig0.319	 2e-104
## Alignment 4: score=313.0 e_value=8.2e-12 N=7 ew0&ew8 plus
  4-  0:	evm.model.Contig0.526	evm.model.Contig8.263	  3e-78
```

```
  4-  1: evm.model.Contig0.527    evm.model.Contig8.270      4e-54
  4-  2: evm.model.Contig0.534    evm.model.Contig8.273          0
  4-  3: evm.model.Contig0.539    evm.model.Contig8.277          0
  4-  4: evm.model.Contig0.541    evm.model.Contig8.278      3e-20
  4-  5: evm.model.Contig0.551    evm.model.Contig8.283          0
  4-  6: evm.model.Contig0.561    evm.model.Contig8.295      2e-95
## Alignment 5: score=278.0 e_value=2.8e-08 N=6 ew0&ew8 plus
  5-  0: evm.model.Contig0.567    evm.model.Contig8.228     7e-117
  5-  1: evm.model.Contig0.570    evm.model.Contig8.230      2e-57
  5-  2: evm.model.Contig0.580    evm.model.Contig8.239          0
  5-  3: evm.model.Contig0.582    evm.model.Contig8.243      3e-23
  5-  4: evm.model.Contig0.587    evm.model.Contig8.248      4e-36
  5-  5: evm.model.Contig0.590    evm.model.Contig8.253      3e-87
## Alignment 6: score=267.0 e_value=2.3e-10 N=6 ew0&ew8 minus
  6-  0: evm.model.Contig0.296    evm.model.Contig8.182      1e-18
  6-  1: evm.model.Contig0.302    evm.model.Contig8.181      2e-70
  6-  2: evm.model.Contig0.310    evm.model.Contig8.176      1e-30
  6-  3: evm.model.Contig0.328    evm.model.Contig8.175      3e-52
  6-  4: evm.model.Contig0.330    evm.model.Contig8.173      4e-55
  6-  5: evm.model.Contig0.334    evm.model.Contig8.169     9e-154
## Alignment 7: score=322.0 e_value=5.1e-13 N=7 ew1&ew1 plus
  7-  0: evm.model.Contig1.1020   evm.model.Contig1.1069    6e-123
  7-  1: evm.model.Contig1.1021   evm.model.Contig1.1072    2e-124
  7-  2: evm.model.Contig1.1024   evm.model.Contig1.1076     1e-68
  7-  3: evm.model.Contig1.1029   evm.model.Contig1.1078         0
  7-  4: evm.model.Contig1.1040   evm.model.Contig1.1090    3e-110
  7-  5: evm.model.Contig1.1041   evm.model.Contig1.1093     4e-17
  7-  6: evm.model.Contig1.1048   evm.model.Contig1.1100     2e-34
## Alignment 8: score=642.0 e_value=9.8e-30 N=13 ew1&ew1 minus
  8-  0: evm.model.Contig1.450    evm.model.Contig1.490          0
  8-  1: evm.model.Contig1.453    evm.model.Contig1.489          0
  8-  2: evm.model.Contig1.454    evm.model.Contig1.488          0
  8-  3: evm.model.Contig1.455    evm.model.Contig1.487          0
  8-  4: evm.model.Contig1.456    evm.model.Contig1.486          0
  8-  5: evm.model.Contig1.457    evm.model.Contig1.485          0
  8-  6: evm.model.Contig1.458    evm.model.Contig1.484      2e-11
  8-  7: evm.model.Contig1.460    evm.model.Contig1.482          0
  8-  8: evm.model.Contig1.461    evm.model.Contig1.481          0
  8-  9: evm.model.Contig1.462    evm.model.Contig1.479          0
  8- 10: evm.model.Contig1.463    evm.model.Contig1.476          0
  8- 11: evm.model.Contig1.464    evm.model.Contig1.473          0
  8- 12: evm.model.Contig1.465    evm.model.Contig1.472          0
## Alignment 9: score=273.0 e_value=1e-11 N=6 ew1&ew1 minus
  9-  0: evm.model.Contig1.1040   evm.model.Contig1.1090    3e-110
  9-  1: evm.model.Contig1.1049   evm.model.Contig1.1083    3e-113
  9-  2: evm.model.Contig1.1059   evm.model.Contig1.1080     3e-71
  9-  3: evm.model.Contig1.1069   evm.model.Contig1.1076    2e-132
  9-  4: evm.model.Contig1.1071   evm.model.Contig1.1074         0
  9-  5: evm.model.Contig1.1072   evm.model.Contig1.1073         0
## Alignment 10: score=588.0 e_value=1.3e-31 N=13 ew1&ew2 plus
 10-  0: evm.model.Contig1.8      evm.model.Contig2.1027         0
 10-  1: evm.model.Contig1.9      evm.model.Contig2.1028         0
 10-  2: evm.model.Contig1.11     evm.model.Contig2.1030     5e-49
 10-  3: evm.model.Contig1.15     evm.model.Contig2.1033     3e-93
```

```
 10-  4: evm.model.Contig1.17     evm.model.Contig2.1036    4e-148
 10-  5: evm.model.Contig1.18     evm.model.Contig2.1037     2e-76
 10-  6: evm.model.Contig1.20     evm.model.Contig2.1053     3e-50
 10-  7: evm.model.Contig1.22     evm.model.Contig2.1059         0
 10-  8: evm.model.Contig1.24     evm.model.Contig2.1060    6e-165
 10-  9: evm.model.Contig1.34     evm.model.Contig2.1069         0
 10- 10: evm.model.Contig1.36     evm.model.Contig2.1079         0
 10- 11: evm.model.Contig1.41     evm.model.Contig2.1089     1e-17
 10- 12: evm.model.Contig1.50     evm.model.Contig2.1096         0
## Alignment 11: score=553.0 e_value=8.2e-26 N=12 ew1&ew2 plus
 11-  0: evm.model.Contig1.142    evm.model.Contig2.555          0
 11-  1: evm.model.Contig1.147    evm.model.Contig2.558     4e-177
 11-  2: evm.model.Contig1.150    evm.model.Contig2.560          0
 11-  3: evm.model.Contig1.153    evm.model.Contig2.561          0
 11-  4: evm.model.Contig1.155    evm.model.Contig2.563          0
 11-  5: evm.model.Contig1.161    evm.model.Contig2.568     8e-158
 11-  6: evm.model.Contig1.167    evm.model.Contig2.570     2e-123
 11-  7: evm.model.Contig1.168    evm.model.Contig2.574          0
 11-  8: evm.model.Contig1.181    evm.model.Contig2.584     5e-176
 11-  9: evm.model.Contig1.182    evm.model.Contig2.586      4e-86
 11- 10: evm.model.Contig1.191    evm.model.Contig2.593          0
 11- 11: evm.model.Contig1.194    evm.model.Contig2.598      2e-92
## Alignment 12: score=411.0 e_value=3.1e-20 N=10 ew1&ew2 plus
 12-  0: evm.model.Contig1.540    evm.model.Contig2.839     3e-153
 12-  1: evm.model.Contig1.542    evm.model.Contig2.852      8e-21
 12-  2: evm.model.Contig1.548    evm.model.Contig2.873      4e-35
 12-  3: evm.model.Contig1.562    evm.model.Contig2.898      1e-31
 12-  4: evm.model.Contig1.573    evm.model.Contig2.899      4e-42
 12-  5: evm.model.Contig1.579    evm.model.Contig2.900          0
 12-  6: evm.model.Contig1.583    evm.model.Contig2.902      8e-62
 12-  7: evm.model.Contig1.587    evm.model.Contig2.906     8e-133
 12-  8: evm.model.Contig1.588    evm.model.Contig2.911     1e-163
 12-  9: evm.model.Contig1.596    evm.model.Contig2.920      3e-60
## Alignment 13: score=405.0 e_value=3.1e-20 N=9 ew1&ew2 plus
 13-  0: evm.model.Contig1.1098   evm.model.Contig2.18      7e-144
 13-  1: evm.model.Contig1.1118   evm.model.Contig2.27       2e-67
 13-  2: evm.model.Contig1.1127   evm.model.Contig2.37           0
 13-  3: evm.model.Contig1.1133   evm.model.Contig2.40           0
 13-  4: evm.model.Contig1.1134   evm.model.Contig2.41      1e-131
 13-  5: evm.model.Contig1.1136   evm.model.Contig2.42      2e-155
 13-  6: evm.model.Contig1.1139   evm.model.Contig2.45       2e-63
 13-  7: evm.model.Contig1.1140   evm.model.Contig2.46      2e-113
 13-  8: evm.model.Contig1.1150   evm.model.Contig2.49           0
## Alignment 14: score=282.0 e_value=8.6e-09 N=6 ew1&ew2 plus
 14-  0: evm.model.Contig1.57     evm.model.Contig2.823     5e-178
 14-  1: evm.model.Contig1.60     evm.model.Contig2.826     3e-160
 14-  2: evm.model.Contig1.66     evm.model.Contig2.830          0
 14-  3: evm.model.Contig1.67     evm.model.Contig2.831          0
 14-  4: evm.model.Contig1.68     evm.model.Contig2.833      2e-51
 14-  5: evm.model.Contig1.79     evm.model.Contig2.835      9e-38
## Alignment 15: score=1159.0 e_value=9.1e-75 N=25 ew1&ew2 minus
 15-  0: evm.model.Contig1.620    evm.model.Contig2.503      8e-54
 15-  1: evm.model.Contig1.621    evm.model.Contig2.502      6e-15
 15-  2: evm.model.Contig1.622    evm.model.Contig2.501      3e-45
```

```
 15-  3: evm.model.Contig1.625    evm.model.Contig2.498      5e-67
 15-  4: evm.model.Contig1.630    evm.model.Contig2.493     4e-123
 15-  5: evm.model.Contig1.631    evm.model.Contig2.491          0
 15-  6: evm.model.Contig1.636    evm.model.Contig2.487          0
 15-  7: evm.model.Contig1.637    evm.model.Contig2.485      9e-41
 15-  8: evm.model.Contig1.638    evm.model.Contig2.483     1e-133
 15-  9: evm.model.Contig1.648    evm.model.Contig2.476      4e-25
 15- 10: evm.model.Contig1.650    evm.model.Contig2.475     5e-151
 15- 11: evm.model.Contig1.661    evm.model.Contig2.469      1e-79
 15- 12: evm.model.Contig1.666    evm.model.Contig2.465          0
 15- 13: evm.model.Contig1.675    evm.model.Contig2.460      6e-65
 15- 14: evm.model.Contig1.677    evm.model.Contig2.456     6e-114
 15- 15: evm.model.Contig1.690    evm.model.Contig2.443          0
 15- 16: evm.model.Contig1.692    evm.model.Contig2.440      6e-28
 15- 17: evm.model.Contig1.696    evm.model.Contig2.433     4e-152
 15- 18: evm.model.Contig1.700    evm.model.Contig2.429      6e-65
 15- 19: evm.model.Contig1.702    evm.model.Contig2.421      2e-38
 15- 20: evm.model.Contig1.703    evm.model.Contig2.419      1e-27
 15- 21: evm.model.Contig1.708    evm.model.Contig2.415     2e-143
 15- 22: evm.model.Contig1.714    evm.model.Contig2.408          0
 15- 23: evm.model.Contig1.715    evm.model.Contig2.407      3e-59
 15- 24: evm.model.Contig1.718    evm.model.Contig2.404      8e-75
## Alignment 16: score=848.0 e_value=7.6e-56 N=19 ew1&ew2 minus
 16-  0: evm.model.Contig1.258    evm.model.Contig2.710      2e-75
 16-  1: evm.model.Contig1.259    evm.model.Contig2.705     2e-180
 16-  2: evm.model.Contig1.264    evm.model.Contig2.699          0
 16-  3: evm.model.Contig1.268    evm.model.Contig2.695          0
 16-  4: evm.model.Contig1.270    evm.model.Contig2.688      8e-50
 16-  5: evm.model.Contig1.278    evm.model.Contig2.687     4e-112
 16-  6: evm.model.Contig1.279    evm.model.Contig2.686          0
 16-  7: evm.model.Contig1.280    evm.model.Contig2.685      2e-88
 16-  8: evm.model.Contig1.290    evm.model.Contig2.670      2e-12
 16-  9: evm.model.Contig1.291    evm.model.Contig2.668      6e-70
 16- 10: evm.model.Contig1.295    evm.model.Contig2.664      9e-35
 16- 11: evm.model.Contig1.301    evm.model.Contig2.640      2e-54
 16- 12: evm.model.Contig1.303    evm.model.Contig2.638     1e-135
 16- 13: evm.model.Contig1.314    evm.model.Contig2.637          0
 16- 14: evm.model.Contig1.317    evm.model.Contig2.635     7e-169
 16- 15: evm.model.Contig1.318    evm.model.Contig2.631     4e-127
 16- 16: evm.model.Contig1.320    evm.model.Contig2.628      6e-22
 16- 17: evm.model.Contig1.321    evm.model.Contig2.610     6e-110
 16- 18: evm.model.Contig1.323    evm.model.Contig2.608      1e-35
## Alignment 17: score=698.0 e_value=3.2e-43 N=16 ew1&ew2 minus
 17-  0: evm.model.Contig1.835    evm.model.Contig2.269      9e-64
 17-  1: evm.model.Contig1.838    evm.model.Contig2.265      1e-25
 17-  2: evm.model.Contig1.839    evm.model.Contig2.262      2e-26
 17-  3: evm.model.Contig1.851    evm.model.Contig2.238          0
 17-  4: evm.model.Contig1.854    evm.model.Contig2.221      3e-58
 17-  5: evm.model.Contig1.855    evm.model.Contig2.220      6e-58
 17-  6: evm.model.Contig1.863    evm.model.Contig2.217          0
 17-  7: evm.model.Contig1.877    evm.model.Contig2.208          0
 17-  8: evm.model.Contig1.878    evm.model.Contig2.203      5e-62
 17-  9: evm.model.Contig1.879    evm.model.Contig2.202      4e-73
 17- 10: evm.model.Contig1.883    evm.model.Contig2.198      2e-20
```

```
 17- 11: evm.model.Contig1.904    evm.model.Contig2.184          0
 17- 12: evm.model.Contig1.905    evm.model.Contig2.177     8e-119
 17- 13: evm.model.Contig1.906    evm.model.Contig2.176          0
 17- 14: evm.model.Contig1.911    evm.model.Contig2.175      6e-55
 17- 15: evm.model.Contig1.913    evm.model.Contig2.174      8e-68
## Alignment 18: score=639.0 e_value=1.6e-33 N=14 ew1&ew2 minus
 18-  0: evm.model.Contig1.66     evm.model.Contig2.820     7e-100
 18-  1: evm.model.Contig1.67     evm.model.Contig2.819          0
 18-  2: evm.model.Contig1.68     evm.model.Contig2.818      1e-53
 18-  3: evm.model.Contig1.80     evm.model.Contig2.817      2e-49
 18-  4: evm.model.Contig1.97     evm.model.Contig2.804      2e-41
 18-  5: evm.model.Contig1.102    evm.model.Contig2.799     2e-180
 18-  6: evm.model.Contig1.104    evm.model.Contig2.795          0
 18-  7: evm.model.Contig1.109    evm.model.Contig2.793      5e-94
 18-  8: evm.model.Contig1.112    evm.model.Contig2.791      3e-86
 18-  9: evm.model.Contig1.116    evm.model.Contig2.786      2e-54
 18- 10: evm.model.Contig1.117    evm.model.Contig2.783     3e-121
 18- 11: evm.model.Contig1.119    evm.model.Contig2.782      7e-18
 18- 12: evm.model.Contig1.127    evm.model.Contig2.775     3e-104
 18- 13: evm.model.Contig1.135    evm.model.Contig2.773      7e-74
## Alignment 19: score=379.0 e_value=4.9e-17 N=9 ew1&ew2 minus
 19-  0: evm.model.Contig1.377    evm.model.Contig2.1012     9e-82
 19-  1: evm.model.Contig1.387    evm.model.Contig2.1010    8e-137
 19-  2: evm.model.Contig1.400    evm.model.Contig2.1007     2e-40
 19-  3: evm.model.Contig1.409    evm.model.Contig2.1006    7e-152
 19-  4: evm.model.Contig1.410    evm.model.Contig2.980      1e-82
 19-  5: evm.model.Contig1.415    evm.model.Contig2.977      5e-30
 19-  6: evm.model.Contig1.420    evm.model.Contig2.973     7e-149
 19-  7: evm.model.Contig1.425    evm.model.Contig2.969          0
 19-  8: evm.model.Contig1.431    evm.model.Contig2.963      7e-76
## Alignment 20: score=332.0 e_value=9.6e-16 N=8 ew1&ew2 minus
 20-  0: evm.model.Contig1.80     evm.model.Contig2.834      2e-51
 20-  1: evm.model.Contig1.82     evm.model.Contig2.833      3e-54
 20-  2: evm.model.Contig1.86     evm.model.Contig2.819          0
 20-  3: evm.model.Contig1.96     evm.model.Contig2.802          0
 20-  4: evm.model.Contig1.111    evm.model.Contig2.794      3e-70
 20-  5: evm.model.Contig1.116    evm.model.Contig2.792      2e-46
 20-  6: evm.model.Contig1.117    evm.model.Contig2.789          0
 20-  7: evm.model.Contig1.136    evm.model.Contig2.774      6e-38
## Alignment 21: score=325.0 e_value=4.2e-11 N=7 ew1&ew2 minus
 21-  0: evm.model.Contig1.216    evm.model.Contig2.551     3e-111
 21-  1: evm.model.Contig1.217    evm.model.Contig2.548     7e-168
 21-  2: evm.model.Contig1.222    evm.model.Contig2.547      1e-73
 21-  3: evm.model.Contig1.226    evm.model.Contig2.540     4e-148
 21-  4: evm.model.Contig1.229    evm.model.Contig2.536          0
 21-  5: evm.model.Contig1.231    evm.model.Contig2.533     9e-136
 21-  6: evm.model.Contig1.234    evm.model.Contig2.524          0
## Alignment 22: score=317.0 e_value=3e-12 N=7 ew1&ew2 minus
 22-  0: evm.model.Contig1.497    evm.model.Contig2.761          0
 22-  1: evm.model.Contig1.502    evm.model.Contig2.755          0
 22-  2: evm.model.Contig1.503    evm.model.Contig2.754     3e-166
 22-  3: evm.model.Contig1.505    evm.model.Contig2.742     2e-131
 22-  4: evm.model.Contig1.507    evm.model.Contig2.740          0
 22-  5: evm.model.Contig1.508    evm.model.Contig2.726     8e-172
```

```
 22-  6: evm.model.Contig1.512    evm.model.Contig2.725          0
## Alignment 23: score=280.0 e_value=5.9e-10 N=6 ew1&ew2 minus
 23-  0: evm.model.Contig1.602    evm.model.Contig2.332      1e-29
 23-  1: evm.model.Contig1.605    evm.model.Contig2.326      3e-25
 23-  2: evm.model.Contig1.608    evm.model.Contig2.320      4e-55
 23-  3: evm.model.Contig1.609    evm.model.Contig2.319          0
 23-  4: evm.model.Contig1.610    evm.model.Contig2.318      2e-99
 23-  5: evm.model.Contig1.614    evm.model.Contig2.307          0
## Alignment 24: score=279.0 e_value=1.1e-08 N=6 ew1&ew2 minus
 24-  0: evm.model.Contig1.756    evm.model.Contig2.364          0
 24-  1: evm.model.Contig1.764    evm.model.Contig2.359          0
 24-  2: evm.model.Contig1.771    evm.model.Contig2.350      2e-26
 24-  3: evm.model.Contig1.775    evm.model.Contig2.349      8e-47
 24-  4: evm.model.Contig1.776    evm.model.Contig2.348     1e-120
 24-  5: evm.model.Contig1.780    evm.model.Contig2.344      4e-40
## Alignment 25: score=274.0 e_value=2.5e-09 N=6 ew1&ew2 minus
 25-  0: evm.model.Contig1.719    evm.model.Contig2.301     1e-170
 25-  1: evm.model.Contig1.721    evm.model.Contig2.299      7e-36
 25-  2: evm.model.Contig1.724    evm.model.Contig2.296     5e-168
 25-  3: evm.model.Contig1.738    evm.model.Contig2.289     2e-119
 25-  4: evm.model.Contig1.739    evm.model.Contig2.280      3e-69
 25-  5: evm.model.Contig1.741    evm.model.Contig2.277      9e-21
## Alignment 26: score=266.0 e_value=2.1e-10 N=6 ew1&ew2 minus
 26-  0: evm.model.Contig1.458    evm.model.Contig2.1047     2e-13
 26-  1: evm.model.Contig1.460    evm.model.Contig2.1046         0
 26-  2: evm.model.Contig1.468    evm.model.Contig2.1041         0
 26-  3: evm.model.Contig1.471    evm.model.Contig2.1038     7e-40
 26-  4: evm.model.Contig1.487    evm.model.Contig2.1015     4e-70
 26-  5: evm.model.Contig1.490    evm.model.Contig2.1014     2e-74
## Alignment 27: score=250.0 e_value=8.9e-09 N=6 ew1&ew2 minus
 27-  0: evm.model.Contig1.665    evm.model.Contig2.473      3e-26
 27-  1: evm.model.Contig1.669    evm.model.Contig2.469      8e-92
 27-  2: evm.model.Contig1.691    evm.model.Contig2.444     3e-104
 27-  3: evm.model.Contig1.699    evm.model.Contig2.428      3e-47
 27-  4: evm.model.Contig1.702    evm.model.Contig2.427      5e-38
 27-  5: evm.model.Contig1.709    evm.model.Contig2.424     1e-107
## Alignment 28: score=275.0 e_value=3.4e-08 N=6 ew10&ew10 plus
 28-  0: evm.model.Contig10.678   evm.model.Contig10.735     3e-42
 28-  1: evm.model.Contig10.682   evm.model.Contig10.737         0
 28-  2: evm.model.Contig10.684   evm.model.Contig10.741     7e-60
 28-  3: evm.model.Contig10.695   evm.model.Contig10.745     2e-49
 28-  4: evm.model.Contig10.697   evm.model.Contig10.748    8e-114
 28-  5: evm.model.Contig10.705   evm.model.Contig10.749     2e-97
## Alignment 29: score=295.0 e_value=2.9e-09 N=6 ew10&ew10 minus
 29-  0: evm.model.Contig10.543   evm.model.Contig10.563         0
 29-  1: evm.model.Contig10.547   evm.model.Contig10.562    9e-112
 29-  2: evm.model.Contig10.548   evm.model.Contig10.561         0
 29-  3: evm.model.Contig10.549   evm.model.Contig10.560         0
 29-  4: evm.model.Contig10.551   evm.model.Contig10.558    8e-100
 29-  5: evm.model.Contig10.553   evm.model.Contig10.557         0
## Alignment 30: score=272.0 e_value=5.2e-11 N=7 ew10&ew10 minus
 30-  0: evm.model.Contig10.659   evm.model.Contig10.786    1e-136
 30-  1: evm.model.Contig10.671   evm.model.Contig10.784     7e-38
 30-  2: evm.model.Contig10.674   evm.model.Contig10.765         0
```

```
 30-  3: evm.model.Contig10.679   evm.model.Contig10.749     1e-51
 30-  4: evm.model.Contig10.682   evm.model.Contig10.746         0
 30-  5: evm.model.Contig10.695   evm.model.Contig10.735     7e-50
 30-  6: evm.model.Contig10.716   evm.model.Contig10.728    8e-162
## Alignment 31: score=983.0 e_value=3.8e-66 N=22 ew10&ew12 plus
 31-  0: evm.model.Contig10.300   evm.model.Contig12.591     1e-24
 31-  1: evm.model.Contig10.303   evm.model.Contig12.596     4e-28
 31-  2: evm.model.Contig10.304   evm.model.Contig12.605    1e-110
 31-  3: evm.model.Contig10.312   evm.model.Contig12.607         0
 31-  4: evm.model.Contig10.313   evm.model.Contig12.608     2e-35
 31-  5: evm.model.Contig10.322   evm.model.Contig12.610     1e-65
 31-  6: evm.model.Contig10.332   evm.model.Contig12.630         0
 31-  7: evm.model.Contig10.333   evm.model.Contig12.635         0
 31-  8: evm.model.Contig10.349   evm.model.Contig12.637    1e-104
 31-  9: evm.model.Contig10.353   evm.model.Contig12.638    1e-161
 31- 10: evm.model.Contig10.356   evm.model.Contig12.639    6e-112
 31- 11: evm.model.Contig10.359   evm.model.Contig12.648    7e-124
 31- 12: evm.model.Contig10.367   evm.model.Contig12.650     1e-26
 31- 13: evm.model.Contig10.379   evm.model.Contig12.658         0
 31- 14: evm.model.Contig10.380   evm.model.Contig12.659     6e-92
 31- 15: evm.model.Contig10.386   evm.model.Contig12.663         0
 31- 16: evm.model.Contig10.393   evm.model.Contig12.668     2e-91
 31- 17: evm.model.Contig10.394   evm.model.Contig12.669     3e-34
 31- 18: evm.model.Contig10.396   evm.model.Contig12.674    3e-130
 31- 19: evm.model.Contig10.397   evm.model.Contig12.676         0
 31- 20: evm.model.Contig10.398   evm.model.Contig12.677     3e-69
 31- 21: evm.model.Contig10.399   evm.model.Contig12.683         0
## Alignment 32: score=607.0 e_value=4.2e-30 N=13 ew10&ew12 plus
 32-  0: evm.model.Contig10.161   evm.model.Contig12.434    2e-130
 32-  1: evm.model.Contig10.162   evm.model.Contig12.435     1e-64
 32-  2: evm.model.Contig10.165   evm.model.Contig12.438         0
 32-  3: evm.model.Contig10.167   evm.model.Contig12.445         0
 32-  4: evm.model.Contig10.169   evm.model.Contig12.452         0
 32-  5: evm.model.Contig10.176   evm.model.Contig12.454         0
 32-  6: evm.model.Contig10.178   evm.model.Contig12.455     1e-90
 32-  7: evm.model.Contig10.180   evm.model.Contig12.457     5e-45
 32-  8: evm.model.Contig10.181   evm.model.Contig12.462    4e-106
 32-  9: evm.model.Contig10.183   evm.model.Contig12.463     1e-43
 32- 10: evm.model.Contig10.184   evm.model.Contig12.465     6e-39
 32- 11: evm.model.Contig10.190   evm.model.Contig12.477     8e-24
 32- 12: evm.model.Contig10.191   evm.model.Contig12.482    2e-133
## Alignment 33: score=601.0 e_value=3.3e-33 N=13 ew10&ew12 plus
 33-  0: evm.model.Contig10.107   evm.model.Contig12.291     6e-46
 33-  1: evm.model.Contig10.108   evm.model.Contig12.293    3e-128
 33-  2: evm.model.Contig10.109   evm.model.Contig12.295         0
 33-  3: evm.model.Contig10.110   evm.model.Contig12.296         0
 33-  4: evm.model.Contig10.111   evm.model.Contig12.297    9e-108
 33-  5: evm.model.Contig10.125   evm.model.Contig12.311    7e-109
 33-  6: evm.model.Contig10.133   evm.model.Contig12.325    4e-124
 33-  7: evm.model.Contig10.135   evm.model.Contig12.327         0
 33-  8: evm.model.Contig10.136   evm.model.Contig12.337         0
 33-  9: evm.model.Contig10.143   evm.model.Contig12.343    4e-147
 33- 10: evm.model.Contig10.144   evm.model.Contig12.344    5e-122
 33- 11: evm.model.Contig10.145   evm.model.Contig12.346     2e-33
```

```
 33- 12: evm.model.Contig10.147   evm.model.Contig12.351    2e-146
## Alignment 34: score=348.0 e_value=1.1e-16 N=9 ew10&ew12 plus
 34-  0: evm.model.Contig10.302   evm.model.Contig12.589    3e-118
 34-  1: evm.model.Contig10.318   evm.model.Contig12.597     4e-43
 34-  2: evm.model.Contig10.326   evm.model.Contig12.600     9e-35
 34-  3: evm.model.Contig10.341   evm.model.Contig12.616         0
 34-  4: evm.model.Contig10.348   evm.model.Contig12.641     6e-48
 34-  5: evm.model.Contig10.349   evm.model.Contig12.643    1e-104
 34-  6: evm.model.Contig10.373   evm.model.Contig12.648    8e-123
 34-  7: evm.model.Contig10.382   evm.model.Contig12.655     1e-43
 34-  8: evm.model.Contig10.392   evm.model.Contig12.662     8e-27
## Alignment 35: score=280.0 e_value=1e-14 N=6 ew10&ew12 plus
 35-  0: evm.model.Contig10.403   evm.model.Contig12.136     3e-21
 35-  1: evm.model.Contig10.411   evm.model.Contig12.156     2e-47
 35-  2: evm.model.Contig10.412   evm.model.Contig12.157     6e-32
 35-  3: evm.model.Contig10.413   evm.model.Contig12.158     2e-35
 35-  4: evm.model.Contig10.415   evm.model.Contig12.159     9e-85
 35-  5: evm.model.Contig10.416   evm.model.Contig12.160    7e-114
## Alignment 36: score=1211.0 e_value=2.2e-86 N=27 ew10&ew12 minus
 36-  0: evm.model.Contig10.433   evm.model.Contig12.264     2e-51
 36-  1: evm.model.Contig10.437   evm.model.Contig12.259         0
 36-  2: evm.model.Contig10.442   evm.model.Contig12.256         0
 36-  3: evm.model.Contig10.449   evm.model.Contig12.246     1e-44
 36-  4: evm.model.Contig10.453   evm.model.Contig12.242     2e-85
 36-  5: evm.model.Contig10.454   evm.model.Contig12.241     3e-34
 36-  6: evm.model.Contig10.458   evm.model.Contig12.237    2e-155
 36-  7: evm.model.Contig10.470   evm.model.Contig12.228         0
 36-  8: evm.model.Contig10.479   evm.model.Contig12.223     6e-88
 36-  9: evm.model.Contig10.484   evm.model.Contig12.220     2e-66
 36- 10: evm.model.Contig10.489   evm.model.Contig12.215     8e-12
 36- 11: evm.model.Contig10.490   evm.model.Contig12.210     8e-56
 36- 12: evm.model.Contig10.491   evm.model.Contig12.209    1e-124
 36- 13: evm.model.Contig10.502   evm.model.Contig12.208         0
 36- 14: evm.model.Contig10.504   evm.model.Contig12.207     3e-12
 36- 15: evm.model.Contig10.505   evm.model.Contig12.206     2e-21
 36- 16: evm.model.Contig10.519   evm.model.Contig12.197    6e-130
 36- 17: evm.model.Contig10.520   evm.model.Contig12.196     3e-97
 36- 18: evm.model.Contig10.521   evm.model.Contig12.195     9e-42
 36- 19: evm.model.Contig10.527   evm.model.Contig12.193     2e-55
 36- 20: evm.model.Contig10.529   evm.model.Contig12.186         0
 36- 21: evm.model.Contig10.537   evm.model.Contig12.184         0
 36- 22: evm.model.Contig10.544   evm.model.Contig12.180    6e-171
 36- 23: evm.model.Contig10.549   evm.model.Contig12.177         0
 36- 24: evm.model.Contig10.550   evm.model.Contig12.176         0
 36- 25: evm.model.Contig10.564   evm.model.Contig12.174     4e-71
 36- 26: evm.model.Contig10.585   evm.model.Contig12.157     7e-93
## Alignment 37: score=884.0 e_value=1.4e-53 N=20 ew10&ew12 minus
 37-  0: evm.model.Contig10.6     evm.model.Contig12.796     1e-75
 37-  1: evm.model.Contig10.13    evm.model.Contig12.790         0
 37-  2: evm.model.Contig10.21    evm.model.Contig12.786    1e-158
 37-  3: evm.model.Contig10.23    evm.model.Contig12.783         0
 37-  4: evm.model.Contig10.30    evm.model.Contig12.781     2e-95
 37-  5: evm.model.Contig10.31    evm.model.Contig12.778         0
 37-  6: evm.model.Contig10.32    evm.model.Contig12.768     4e-25
```

```
 37-  7: evm.model.Contig10.38    evm.model.Contig12.763     4e-55
 37-  8: evm.model.Contig10.46    evm.model.Contig12.745     1e-24
 37-  9: evm.model.Contig10.51    evm.model.Contig12.739    9e-117
 37- 10: evm.model.Contig10.52    evm.model.Contig12.737    7e-151
 37- 11: evm.model.Contig10.55    evm.model.Contig12.733         0
 37- 12: evm.model.Contig10.57    evm.model.Contig12.727         0
 37- 13: evm.model.Contig10.60    evm.model.Contig12.721         0
 37- 14: evm.model.Contig10.62    evm.model.Contig12.717    4e-138
 37- 15: evm.model.Contig10.75    evm.model.Contig12.711         0
 37- 16: evm.model.Contig10.82    evm.model.Contig12.709         0
 37- 17: evm.model.Contig10.84    evm.model.Contig12.708         0
 37- 18: evm.model.Contig10.89    evm.model.Contig12.691         0
 37- 19: evm.model.Contig10.93    evm.model.Contig12.685     3e-99
## Alignment 38: score=560.0 e_value=5.2e-34 N=14 ew10&ew12 minus
 38-  0: evm.model.Contig10.187   evm.model.Contig12.616    1e-152
 38-  1: evm.model.Contig10.205   evm.model.Contig12.591     2e-27
 38-  2: evm.model.Contig10.207   evm.model.Contig12.589     3e-49
 38-  3: evm.model.Contig10.211   evm.model.Contig12.585    2e-169
 38-  4: evm.model.Contig10.224   evm.model.Contig12.560     2e-40
 38-  5: evm.model.Contig10.237   evm.model.Contig12.543    3e-100
 38-  6: evm.model.Contig10.240   evm.model.Contig12.528     1e-41
 38-  7: evm.model.Contig10.243   evm.model.Contig12.520    1e-150
 38-  8: evm.model.Contig10.257   evm.model.Contig12.518     2e-94
 38-  9: evm.model.Contig10.262   evm.model.Contig12.510     2e-64
 38- 10: evm.model.Contig10.265   evm.model.Contig12.500    1e-138
 38- 11: evm.model.Contig10.274   evm.model.Contig12.499     2e-50
 38- 12: evm.model.Contig10.284   evm.model.Contig12.498     9e-47
 38- 13: evm.model.Contig10.290   evm.model.Contig12.492         0
## Alignment 39: score=379.0 e_value=4.6e-18 N=9 ew10&ew12 minus
 39-  0: evm.model.Contig10.117   evm.model.Contig12.393    9e-143
 39-  1: evm.model.Contig10.118   evm.model.Contig12.391    6e-113
 39-  2: evm.model.Contig10.126   evm.model.Contig12.378    2e-161
 39-  3: evm.model.Contig10.127   evm.model.Contig12.370     8e-98
 39-  4: evm.model.Contig10.141   evm.model.Contig12.351    2e-144
 39-  5: evm.model.Contig10.143   evm.model.Contig12.349     2e-34
 39-  6: evm.model.Contig10.149   evm.model.Contig12.339     2e-21
 39-  7: evm.model.Contig10.153   evm.model.Contig12.328         0
 39-  8: evm.model.Contig10.159   evm.model.Contig12.314     2e-51
## Alignment 40: score=333.0 e_value=1e-15 N=8 ew10&ew12 minus
 40-  0: evm.model.Contig10.201   evm.model.Contig12.568     7e-21
 40-  1: evm.model.Contig10.202   evm.model.Contig12.567     3e-27
 40-  2: evm.model.Contig10.220   evm.model.Contig12.555    2e-117
 40-  3: evm.model.Contig10.221   evm.model.Contig12.554    1e-112
 40-  4: evm.model.Contig10.230   evm.model.Contig12.539    1e-113
 40-  5: evm.model.Contig10.239   evm.model.Contig12.522     3e-68
 40-  6: evm.model.Contig10.254   evm.model.Contig12.506     1e-43
 40-  7: evm.model.Contig10.256   evm.model.Contig12.500    8e-139
## Alignment 41: score=324.0 e_value=1.1e-11 N=7 ew10&ew12 minus
 41-  0: evm.model.Contig10.573   evm.model.Contig12.142         0
 41-  1: evm.model.Contig10.575   evm.model.Contig12.140     6e-46
 41-  2: evm.model.Contig10.577   evm.model.Contig12.139     3e-51
 41-  3: evm.model.Contig10.578   evm.model.Contig12.138    3e-166
 41-  4: evm.model.Contig10.582   evm.model.Contig12.134     1e-42
 41-  5: evm.model.Contig10.583   evm.model.Contig12.128         0
```

```
 41-  6: evm.model.Contig10.590   evm.model.Contig12.111     1e-47
## Alignment 42: score=284.0 e_value=3.4e-12 N=7 ew10&ew12 minus
 42-  0: evm.model.Contig10.108   evm.model.Contig12.383    1e-128
 42-  1: evm.model.Contig10.120   evm.model.Contig12.357    4e-119
 42-  2: evm.model.Contig10.123   evm.model.Contig12.356     8e-29
 42-  3: evm.model.Contig10.124   evm.model.Contig12.355         0
 42-  4: evm.model.Contig10.143   evm.model.Contig12.335     2e-16
 42-  5: evm.model.Contig10.149   evm.model.Contig12.329     8e-42
 42-  6: evm.model.Contig10.157   evm.model.Contig12.313     7e-45
## Alignment 43: score=277.0 e_value=1e-08 N=6 ew10&ew12 minus
 43-  0: evm.model.Contig10.400   evm.model.Contig12.168     4e-99
 43-  1: evm.model.Contig10.409   evm.model.Contig12.161         0
 43-  2: evm.model.Contig10.413   evm.model.Contig12.158     2e-35
 43-  3: evm.model.Contig10.417   evm.model.Contig12.156    3e-137
 43-  4: evm.model.Contig10.421   evm.model.Contig12.155    1e-124
 43-  5: evm.model.Contig10.423   evm.model.Contig12.148    1e-112
## Alignment 44: score=300.0 e_value=9.8e-09 N=6 ew11&ew11 plus
 44-  0: evm.model.Contig11.237   evm.model.Contig11.262         0
 44-  1: evm.model.Contig11.238   evm.model.Contig11.263         0
 44-  2: evm.model.Contig11.239   evm.model.Contig11.264     9e-33
 44-  3: evm.model.Contig11.240   evm.model.Contig11.265     1e-81
 44-  4: evm.model.Contig11.241   evm.model.Contig11.266     5e-53
 44-  5: evm.model.Contig11.242   evm.model.Contig11.267         0
## Alignment 45: score=324.0 e_value=7.1e-13 N=7 ew11&ew11 minus
 45-  0: evm.model.Contig11.357   evm.model.Contig11.396         0
 45-  1: evm.model.Contig11.360   evm.model.Contig11.395         0
 45-  2: evm.model.Contig11.363   evm.model.Contig11.394         0
 45-  3: evm.model.Contig11.364   evm.model.Contig11.393    7e-177
 45-  4: evm.model.Contig11.365   evm.model.Contig11.391     2e-97
 45-  5: evm.model.Contig11.366   evm.model.Contig11.370    2e-156
 45-  6: evm.model.Contig11.367   evm.model.Contig11.368     3e-68
## Alignment 46: score=460.0 e_value=8e-21 N=10 ew11&ew23 plus
 46-  0: evm.model.Contig11.53    evm.model.Contig23.432     1e-83
 46-  1: evm.model.Contig11.54    evm.model.Contig23.436    2e-156
 46-  2: evm.model.Contig11.55    evm.model.Contig23.446         0
 46-  3: evm.model.Contig11.56    evm.model.Contig23.447     3e-18
 46-  4: evm.model.Contig11.66    evm.model.Contig23.448         0
 46-  5: evm.model.Contig11.69    evm.model.Contig23.456    3e-125
 46-  6: evm.model.Contig11.70    evm.model.Contig23.458     4e-41
 46-  7: evm.model.Contig11.71    evm.model.Contig23.459         0
 46-  8: evm.model.Contig11.80    evm.model.Contig23.464     5e-38
 46-  9: evm.model.Contig11.84    evm.model.Contig23.466     2e-78
## Alignment 47: score=414.0 e_value=2.4e-20 N=10 ew11&ew23 plus
 47-  0: evm.model.Contig11.389   evm.model.Contig23.548     5e-33
 47-  1: evm.model.Contig11.390   evm.model.Contig23.549     1e-17
 47-  2: evm.model.Contig11.397   evm.model.Contig23.552     4e-37
 47-  3: evm.model.Contig11.402   evm.model.Contig23.557     2e-76
 47-  4: evm.model.Contig11.415   evm.model.Contig23.565    6e-167
 47-  5: evm.model.Contig11.421   evm.model.Contig23.571     6e-65
 47-  6: evm.model.Contig11.444   evm.model.Contig23.591     7e-97
 47-  7: evm.model.Contig11.453   evm.model.Contig23.612     1e-69
 47-  8: evm.model.Contig11.466   evm.model.Contig23.613     1e-89
 47-  9: evm.model.Contig11.472   evm.model.Contig23.616     2e-99
## Alignment 48: score=310.0 e_value=3.3e-11 N=7 ew11&ew23 plus
```

```
 48-  0: evm.model.Contig11.8      evm.model.Contig23.482     2e-14
 48-  1: evm.model.Contig11.22     evm.model.Contig23.492         0
 48-  2: evm.model.Contig11.26     evm.model.Contig23.498     2e-81
 48-  3: evm.model.Contig11.28     evm.model.Contig23.506         0
 48-  4: evm.model.Contig11.29     evm.model.Contig23.516    2e-112
 48-  5: evm.model.Contig11.35     evm.model.Contig23.522    2e-169
 48-  6: evm.model.Contig11.36     evm.model.Contig23.524    2e-142
## Alignment 49: score=2765.0 e_value=3.7e-238 N=61 ew11&ew23 minus
 49-  0: evm.model.Contig11.90     evm.model.Contig23.426    8e-149
 49-  1: evm.model.Contig11.97     evm.model.Contig23.424     2e-37
 49-  2: evm.model.Contig11.101    evm.model.Contig23.422     1e-18
 49-  3: evm.model.Contig11.102    evm.model.Contig23.416     2e-27
 49-  4: evm.model.Contig11.105    evm.model.Contig23.412     1e-19
 49-  5: evm.model.Contig11.118    evm.model.Contig23.409     6e-18
 49-  6: evm.model.Contig11.122    evm.model.Contig23.403     2e-66
 49-  7: evm.model.Contig11.132    evm.model.Contig23.399     4e-57
 49-  8: evm.model.Contig11.133    evm.model.Contig23.398     5e-83
 49-  9: evm.model.Contig11.137    evm.model.Contig23.391     5e-80
 49- 10: evm.model.Contig11.142    evm.model.Contig23.389    2e-174
 49- 11: evm.model.Contig11.156    evm.model.Contig23.387    7e-149
 49- 12: evm.model.Contig11.157    evm.model.Contig23.381     6e-23
 49- 13: evm.model.Contig11.161    evm.model.Contig23.379         0
 49- 14: evm.model.Contig11.162    evm.model.Contig23.377     7e-41
 49- 15: evm.model.Contig11.165    evm.model.Contig23.375     6e-44
 49- 16: evm.model.Contig11.166    evm.model.Contig23.374         0
 49- 17: evm.model.Contig11.167    evm.model.Contig23.373         0
 49- 18: evm.model.Contig11.180    evm.model.Contig23.357     5e-53
 49- 19: evm.model.Contig11.182    evm.model.Contig23.350     3e-18
 49- 20: evm.model.Contig11.186    evm.model.Contig23.348     3e-25
 49- 21: evm.model.Contig11.201    evm.model.Contig23.347    2e-177
 49- 22: evm.model.Contig11.203    evm.model.Contig23.345     2e-85
 49- 23: evm.model.Contig11.204    evm.model.Contig23.344    2e-137
 49- 24: evm.model.Contig11.212    evm.model.Contig23.338         0
 49- 25: evm.model.Contig11.219    evm.model.Contig23.332         0
 49- 26: evm.model.Contig11.224    evm.model.Contig23.328     2e-95
 49- 27: evm.model.Contig11.229    evm.model.Contig23.325    4e-153
 49- 28: evm.model.Contig11.234    evm.model.Contig23.323    6e-119
 49- 29: evm.model.Contig11.244    evm.model.Contig23.322     2e-99
 49- 30: evm.model.Contig11.245    evm.model.Contig23.321         0
 49- 31: evm.model.Contig11.249    evm.model.Contig23.320         0
 49- 32: evm.model.Contig11.256    evm.model.Contig23.310    7e-119
 49- 33: evm.model.Contig11.257    evm.model.Contig23.309     8e-26
 49- 34: evm.model.Contig11.268    evm.model.Contig23.304         0
 49- 35: evm.model.Contig11.269    evm.model.Contig23.301     5e-59
 49- 36: evm.model.Contig11.271    evm.model.Contig23.300     1e-18
 49- 37: evm.model.Contig11.272    evm.model.Contig23.298     1e-96
 49- 38: evm.model.Contig11.275    evm.model.Contig23.297     5e-73
 49- 39: evm.model.Contig11.296    evm.model.Contig23.282     1e-73
 49- 40: evm.model.Contig11.300    evm.model.Contig23.280    2e-172
 49- 41: evm.model.Contig11.301    evm.model.Contig23.278    2e-124
 49- 42: evm.model.Contig11.302    evm.model.Contig23.277     1e-13
 49- 43: evm.model.Contig11.306    evm.model.Contig23.272         0
 49- 44: evm.model.Contig11.314    evm.model.Contig23.269         0
 49- 45: evm.model.Contig11.325    evm.model.Contig23.259    7e-141
```

```
 49- 46: evm.model.Contig11.330   evm.model.Contig23.253    1e-165
 49- 47: evm.model.Contig11.332   evm.model.Contig23.246     4e-93
 49- 48: evm.model.Contig11.333   evm.model.Contig23.237     9e-29
 49- 49: evm.model.Contig11.335   evm.model.Contig23.235         0
 49- 50: evm.model.Contig11.336   evm.model.Contig23.233     2e-72
 49- 51: evm.model.Contig11.337   evm.model.Contig23.232     3e-73
 49- 52: evm.model.Contig11.338   evm.model.Contig23.231    1e-100
 49- 53: evm.model.Contig11.345   evm.model.Contig23.230     3e-28
 49- 54: evm.model.Contig11.348   evm.model.Contig23.227     2e-85
 49- 55: evm.model.Contig11.353   evm.model.Contig23.225     7e-49
 49- 56: evm.model.Contig11.357   evm.model.Contig23.223    5e-168
 49- 57: evm.model.Contig11.361   evm.model.Contig23.221     3e-51
 49- 58: evm.model.Contig11.380   evm.model.Contig23.209    8e-130
 49- 59: evm.model.Contig11.386   evm.model.Contig23.204         0
 49- 60: evm.model.Contig11.387   evm.model.Contig23.203     4e-51
## Alignment 50: score=471.0 e_value=4.7e-25 N=11 ew11&ew23 minus
 50-  0: evm.model.Contig11.620   evm.model.Contig23.76      2e-44
 50-  1: evm.model.Contig11.642   evm.model.Contig23.73          0
 50-  2: evm.model.Contig11.645   evm.model.Contig23.72          0
 50-  3: evm.model.Contig11.654   evm.model.Contig23.51          0
 50-  4: evm.model.Contig11.660   evm.model.Contig23.41      1e-12
 50-  5: evm.model.Contig11.663   evm.model.Contig23.27      4e-95
 50-  6: evm.model.Contig11.669   evm.model.Contig23.24      4e-94
 50-  7: evm.model.Contig11.671   evm.model.Contig23.22      5e-62
 50-  8: evm.model.Contig11.672   evm.model.Contig23.21     3e-133
 50-  9: evm.model.Contig11.674   evm.model.Contig23.18     2e-115
 50- 10: evm.model.Contig11.681   evm.model.Contig23.14     7e-150
## Alignment 51: score=373.0 e_value=2e-19 N=9 ew11&ew23 minus
 51-  0: evm.model.Contig11.16    evm.model.Contig23.533    4e-119
 51-  1: evm.model.Contig11.22    evm.model.Contig23.527         0
 51-  2: evm.model.Contig11.27    evm.model.Contig23.507         0
 51-  3: evm.model.Contig11.28    evm.model.Contig23.506         0
 51-  4: evm.model.Contig11.37    evm.model.Contig23.482         0
 51-  5: evm.model.Contig11.43    evm.model.Contig23.474         0
 51-  6: evm.model.Contig11.45    evm.model.Contig23.473         0
 51-  7: evm.model.Contig11.47    evm.model.Contig23.470    6e-108
 51-  8: evm.model.Contig11.68    evm.model.Contig23.457     4e-37
## Alignment 52: score=355.0 e_value=1.6e-13 N=8 ew11&ew23 minus
 52-  0: evm.model.Contig11.482   evm.model.Contig23.667         0
 52-  1: evm.model.Contig11.492   evm.model.Contig23.658     1e-90
 52-  2: evm.model.Contig11.493   evm.model.Contig23.655         0
 52-  3: evm.model.Contig11.499   evm.model.Contig23.650         0
 52-  4: evm.model.Contig11.501   evm.model.Contig23.645         0
 52-  5: evm.model.Contig11.507   evm.model.Contig23.642     7e-64
 52-  6: evm.model.Contig11.523   evm.model.Contig23.631     3e-79
 52-  7: evm.model.Contig11.529   evm.model.Contig23.625    2e-163
## Alignment 53: score=254.0 e_value=1.7e-10 N=7 ew11&ew23 minus
 53-  0: evm.model.Contig11.298   evm.model.Contig23.286         0
 53-  1: evm.model.Contig11.311   evm.model.Contig23.264         0
 53-  2: evm.model.Contig11.327   evm.model.Contig23.252    3e-130
 53-  3: evm.model.Contig11.344   evm.model.Contig23.235         0
 53-  4: evm.model.Contig11.362   evm.model.Contig23.224     1e-53
 53-  5: evm.model.Contig11.378   evm.model.Contig23.221     2e-46
 53-  6: evm.model.Contig11.391   evm.model.Contig23.209    2e-143
```

```
## Alignment 54: score=439.0 e_value=3.3e-17 N=9 ew12&ew12 plus
 54-  0: evm.model.Contig12.241   evm.model.Contig12.406        0
 54-  1: evm.model.Contig12.243   evm.model.Contig12.409        0
 54-  2: evm.model.Contig12.244   evm.model.Contig12.412        0
 54-  3: evm.model.Contig12.246   evm.model.Contig12.414   4e-172
 54-  4: evm.model.Contig12.247   evm.model.Contig12.416        0
 54-  5: evm.model.Contig12.251   evm.model.Contig12.417    3e-43
 54-  6: evm.model.Contig12.252   evm.model.Contig12.418   2e-148
 54-  7: evm.model.Contig12.253   evm.model.Contig12.419        0
 54-  8: evm.model.Contig12.256   evm.model.Contig12.420        0
## Alignment 55: score=394.0 e_value=1.9e-15 N=8 ew12&ew12 plus
 55-  0: evm.model.Contig12.293   evm.model.Contig12.383        0
 55-  1: evm.model.Contig12.294   evm.model.Contig12.384        0
 55-  2: evm.model.Contig12.295   evm.model.Contig12.385        0
 55-  3: evm.model.Contig12.296   evm.model.Contig12.386        0
 55-  4: evm.model.Contig12.297   evm.model.Contig12.387        0
 55-  5: evm.model.Contig12.298   evm.model.Contig12.388   5e-165
 55-  6: evm.model.Contig12.299   evm.model.Contig12.389        0
 55-  7: evm.model.Contig12.300   evm.model.Contig12.396        0
## Alignment 56: score=527.0 e_value=1.3e-23 N=11 ew12&ew12 minus
 56-  0: evm.model.Contig12.275   evm.model.Contig12.393    2e-45
 56-  1: evm.model.Contig12.280   evm.model.Contig12.391        0
 56-  2: evm.model.Contig12.281   evm.model.Contig12.390        0
 56-  3: evm.model.Contig12.283   evm.model.Contig12.389        0
 56-  4: evm.model.Contig12.284   evm.model.Contig12.388    7e-62
 56-  5: evm.model.Contig12.285   evm.model.Contig12.387        0
 56-  6: evm.model.Contig12.286   evm.model.Contig12.386        0
 56-  7: evm.model.Contig12.287   evm.model.Contig12.383        0
 56-  8: evm.model.Contig12.304   evm.model.Contig12.381        0
 56-  9: evm.model.Contig12.305   evm.model.Contig12.380        0
 56- 10: evm.model.Contig12.306   evm.model.Contig12.379    2e-42
## Alignment 57: score=484.0 e_value=1.1e-26 N=10 ew12&ew12 minus
 57-  0: evm.model.Contig12.1     evm.model.Contig12.49         0
 57-  1: evm.model.Contig12.3     evm.model.Contig12.48         0
 57-  2: evm.model.Contig12.4     evm.model.Contig12.47    3e-103
 57-  3: evm.model.Contig12.5     evm.model.Contig12.46         0
 57-  4: evm.model.Contig12.6     evm.model.Contig12.45     6e-54
 57-  5: evm.model.Contig12.7     evm.model.Contig12.44    3e-173
 57-  6: evm.model.Contig12.8     evm.model.Contig12.43    9e-106
 57-  7: evm.model.Contig12.9     evm.model.Contig12.42         0
 57-  8: evm.model.Contig12.10    evm.model.Contig12.39    8e-145
 57-  9: evm.model.Contig12.23    evm.model.Contig12.25     2e-64
## Alignment 58: score=298.0 e_value=1.3e-08 N=6 ew12&ew2 minus
 58-  0: evm.model.Contig12.156   evm.model.Contig2.653    9e-105
 58-  1: evm.model.Contig12.157   evm.model.Contig2.651         0
 58-  2: evm.model.Contig12.158   evm.model.Contig2.650     2e-35
 58-  3: evm.model.Contig12.159   evm.model.Contig2.649    1e-107
 58-  4: evm.model.Contig12.160   evm.model.Contig2.648    4e-123
 58-  5: evm.model.Contig12.161   evm.model.Contig2.646         0
## Alignment 59: score=335.0 e_value=4.9e-13 N=7 ew13&ew13 plus
 59-  0: evm.model.Contig13.399   evm.model.Contig13.452        0
 59-  1: evm.model.Contig13.400   evm.model.Contig13.465        0
 59-  2: evm.model.Contig13.401   evm.model.Contig13.466        0
 59-  3: evm.model.Contig13.402   evm.model.Contig13.467        0
```

```
 59-  4: evm.model.Contig13.403   evm.model.Contig13.468         0
 59-  5: evm.model.Contig13.404   evm.model.Contig13.469         0
 59-  6: evm.model.Contig13.405   evm.model.Contig13.473         0
## Alignment 60: score=331.0 e_value=2e-13 N=7 ew13&ew13 minus
 60-  0: evm.model.Contig13.5     evm.model.Contig13.148         0
 60-  1: evm.model.Contig13.7     evm.model.Contig13.146     1e-75
 60-  2: evm.model.Contig13.10    evm.model.Contig13.130         0
 60-  3: evm.model.Contig13.12    evm.model.Contig13.129         0
 60-  4: evm.model.Contig13.13    evm.model.Contig13.128         0
 60-  5: evm.model.Contig13.14    evm.model.Contig13.127         0
 60-  6: evm.model.Contig13.17    evm.model.Contig13.126         0
## Alignment 61: score=631.0 e_value=4.6e-34 N=14 ew13&ew4 plus
 61-  0: evm.model.Contig13.14    evm.model.Contig4.662          0
 61-  1: evm.model.Contig13.17    evm.model.Contig4.666      2e-54
 61-  2: evm.model.Contig13.19    evm.model.Contig4.667          0
 61-  3: evm.model.Contig13.20    evm.model.Contig4.669     2e-140
 61-  4: evm.model.Contig13.21    evm.model.Contig4.674     7e-144
 61-  5: evm.model.Contig13.25    evm.model.Contig4.688      9e-41
 61-  6: evm.model.Contig13.29    evm.model.Contig4.689          0
 61-  7: evm.model.Contig13.31    evm.model.Contig4.691          0
 61-  8: evm.model.Contig13.36    evm.model.Contig4.692          0
 61-  9: evm.model.Contig13.40    evm.model.Contig4.701     2e-136
 61- 10: evm.model.Contig13.44    evm.model.Contig4.705     1e-135
 61- 11: evm.model.Contig13.45    evm.model.Contig4.711          0
 61- 12: evm.model.Contig13.46    evm.model.Contig4.712          0
 61- 13: evm.model.Contig13.70    evm.model.Contig4.730          0
## Alignment 62: score=568.0 e_value=2.9e-27 N=12 ew13&ew4 plus
 62-  0: evm.model.Contig13.668   evm.model.Contig4.741          0
 62-  1: evm.model.Contig13.672   evm.model.Contig4.743          0
 62-  2: evm.model.Contig13.673   evm.model.Contig4.745      5e-84
 62-  3: evm.model.Contig13.682   evm.model.Contig4.754     5e-129
 62-  4: evm.model.Contig13.683   evm.model.Contig4.755      1e-91
 62-  5: evm.model.Contig13.687   evm.model.Contig4.757      4e-99
 62-  6: evm.model.Contig13.690   evm.model.Contig4.765      8e-53
 62-  7: evm.model.Contig13.691   evm.model.Contig4.766      7e-66
 62-  8: evm.model.Contig13.692   evm.model.Contig4.767          0
 62-  9: evm.model.Contig13.697   evm.model.Contig4.773      2e-96
 62- 10: evm.model.Contig13.698   evm.model.Contig4.777          0
 62- 11: evm.model.Contig13.700   evm.model.Contig4.780      2e-81
## Alignment 63: score=536.0 e_value=8.3e-28 N=12 ew13&ew4 plus
 63-  0: evm.model.Contig13.415   evm.model.Contig4.251      1e-12
 63-  1: evm.model.Contig13.420   evm.model.Contig4.252     7e-166
 63-  2: evm.model.Contig13.428   evm.model.Contig4.261      2e-48
 63-  3: evm.model.Contig13.431   evm.model.Contig4.269      6e-83
 63-  4: evm.model.Contig13.432   evm.model.Contig4.271          0
 63-  5: evm.model.Contig13.436   evm.model.Contig4.273      2e-81
 63-  6: evm.model.Contig13.438   evm.model.Contig4.278      4e-57
 63-  7: evm.model.Contig13.443   evm.model.Contig4.282      2e-43
 63-  8: evm.model.Contig13.444   evm.model.Contig4.284      4e-51
 63-  9: evm.model.Contig13.449   evm.model.Contig4.287      3e-15
 63- 10: evm.model.Contig13.450   evm.model.Contig4.294     1e-101
 63- 11: evm.model.Contig13.471   evm.model.Contig4.317          0
## Alignment 64: score=361.0 e_value=1.1e-13 N=8 ew13&ew4 plus
 64-  0: evm.model.Contig13.596   evm.model.Contig4.861      1e-61
```

```
 64-  1: evm.model.Contig13.600   evm.model.Contig4.865      1e-12
 64-  2: evm.model.Contig13.603   evm.model.Contig4.875     1e-100
 64-  3: evm.model.Contig13.605   evm.model.Contig4.878          0
 64-  4: evm.model.Contig13.606   evm.model.Contig4.880          0
 64-  5: evm.model.Contig13.614   evm.model.Contig4.893          0
 64-  6: evm.model.Contig13.615   evm.model.Contig4.903      1e-74
 64-  7: evm.model.Contig13.619   evm.model.Contig4.906      3e-20
## Alignment 65: score=315.0 e_value=9.3e-13 N=7 ew13&ew4 plus
 65-  0: evm.model.Contig13.451   evm.model.Contig4.328          0
 65-  1: evm.model.Contig13.455   evm.model.Contig4.334      3e-34
 65-  2: evm.model.Contig13.458   evm.model.Contig4.339     7e-144
 65-  3: evm.model.Contig13.465   evm.model.Contig4.340     4e-120
 65-  4: evm.model.Contig13.467   evm.model.Contig4.341     1e-136
 65-  5: evm.model.Contig13.468   evm.model.Contig4.342     1e-113
 65-  6: evm.model.Contig13.473   evm.model.Contig4.362     2e-118
## Alignment 66: score=255.0 e_value=3.4e-09 N=6 ew13&ew4 plus
 66-  0: evm.model.Contig13.416   evm.model.Contig4.246      1e-57
 66-  1: evm.model.Contig13.429   evm.model.Contig4.257      3e-30
 66-  2: evm.model.Contig13.432   evm.model.Contig4.262          0
 66-  3: evm.model.Contig13.436   evm.model.Contig4.264      4e-82
 66-  4: evm.model.Contig13.438   evm.model.Contig4.268      1e-53
 66-  5: evm.model.Contig13.443   evm.model.Contig4.292      7e-43
## Alignment 67: score=1291.0 e_value=1.7e-95 N=29 ew13&ew4 minus
 67-  0: evm.model.Contig13.286   evm.model.Contig4.453      2e-43
 67-  1: evm.model.Contig13.289   evm.model.Contig4.450      3e-19
 67-  2: evm.model.Contig13.312   evm.model.Contig4.436          0
 67-  3: evm.model.Contig13.319   evm.model.Contig4.419          0
 67-  4: evm.model.Contig13.320   evm.model.Contig4.418          0
 67-  5: evm.model.Contig13.324   evm.model.Contig4.415      7e-30
 67-  6: evm.model.Contig13.326   evm.model.Contig4.407          0
 67-  7: evm.model.Contig13.331   evm.model.Contig4.406     3e-113
 67-  8: evm.model.Contig13.333   evm.model.Contig4.405          0
 67-  9: evm.model.Contig13.334   evm.model.Contig4.401      9e-94
 67- 10: evm.model.Contig13.342   evm.model.Contig4.396          0
 67- 11: evm.model.Contig13.343   evm.model.Contig4.393     3e-111
 67- 12: evm.model.Contig13.344   evm.model.Contig4.392          0
 67- 13: evm.model.Contig13.351   evm.model.Contig4.391      1e-54
 67- 14: evm.model.Contig13.361   evm.model.Contig4.386      3e-21
 67- 15: evm.model.Contig13.369   evm.model.Contig4.377     5e-133
 67- 16: evm.model.Contig13.371   evm.model.Contig4.373     9e-138
 67- 17: evm.model.Contig13.373   evm.model.Contig4.369      4e-34
 67- 18: evm.model.Contig13.382   evm.model.Contig4.358      1e-75
 67- 19: evm.model.Contig13.389   evm.model.Contig4.353      8e-80
 67- 20: evm.model.Contig13.390   evm.model.Contig4.352      3e-59
 67- 21: evm.model.Contig13.397   evm.model.Contig4.338      2e-95
 67- 22: evm.model.Contig13.400   evm.model.Contig4.333          0
 67- 23: evm.model.Contig13.402   evm.model.Contig4.327     1e-143
 67- 24: evm.model.Contig13.403   evm.model.Contig4.326     3e-174
 67- 25: evm.model.Contig13.404   evm.model.Contig4.325          0
 67- 26: evm.model.Contig13.405   evm.model.Contig4.317     4e-109
 67- 27: evm.model.Contig13.407   evm.model.Contig4.313     2e-117
 67- 28: evm.model.Contig13.412   evm.model.Contig4.297     6e-118
## Alignment 68: score=664.0 e_value=1.2e-38 N=15 ew13&ew4 minus
 68-  0: evm.model.Contig13.230   evm.model.Contig4.539     5e-114
```

```
 68-  1: evm.model.Contig13.234   evm.model.Contig4.536     1e-111
 68-  2: evm.model.Contig13.245   evm.model.Contig4.529     7e-160
 68-  3: evm.model.Contig13.246   evm.model.Contig4.528      7e-26
 68-  4: evm.model.Contig13.248   evm.model.Contig4.525     9e-126
 68-  5: evm.model.Contig13.251   evm.model.Contig4.518      3e-85
 68-  6: evm.model.Contig13.252   evm.model.Contig4.517      4e-32
 68-  7: evm.model.Contig13.253   evm.model.Contig4.514      4e-56
 68-  8: evm.model.Contig13.261   evm.model.Contig4.508      5e-72
 68-  9: evm.model.Contig13.271   evm.model.Contig4.499     2e-142
 68- 10: evm.model.Contig13.278   evm.model.Contig4.498      2e-50
 68- 11: evm.model.Contig13.279   evm.model.Contig4.492      5e-37
 68- 12: evm.model.Contig13.282   evm.model.Contig4.487          0
 68- 13: evm.model.Contig13.296   evm.model.Contig4.477     7e-110
 68- 14: evm.model.Contig13.316   evm.model.Contig4.460      1e-63
## Alignment 69: score=626.0 e_value=1.9e-39 N=15 ew13&ew4 minus
 69-  0: evm.model.Contig13.497   evm.model.Contig4.209     3e-172
 69-  1: evm.model.Contig13.503   evm.model.Contig4.208     1e-174
 69-  2: evm.model.Contig13.513   evm.model.Contig4.188      1e-28
 69-  3: evm.model.Contig13.523   evm.model.Contig4.173          0
 69-  4: evm.model.Contig13.525   evm.model.Contig4.163          0
 69-  5: evm.model.Contig13.533   evm.model.Contig4.150      1e-60
 69-  6: evm.model.Contig13.535   evm.model.Contig4.148      1e-45
 69-  7: evm.model.Contig13.542   evm.model.Contig4.143          0
 69-  8: evm.model.Contig13.543   evm.model.Contig4.142          0
 69-  9: evm.model.Contig13.554   evm.model.Contig4.128     6e-114
 69- 10: evm.model.Contig13.555   evm.model.Contig4.127      2e-31
 69- 11: evm.model.Contig13.569   evm.model.Contig4.122          0
 69- 12: evm.model.Contig13.572   evm.model.Contig4.118      4e-49
 69- 13: evm.model.Contig13.574   evm.model.Contig4.100      7e-99
 69- 14: evm.model.Contig13.584   evm.model.Contig4.87       8e-60
## Alignment 70: score=586.0 e_value=3.2e-29 N=13 ew13&ew4 minus
 70-  0: evm.model.Contig13.596   evm.model.Contig4.868      3e-97
 70-  1: evm.model.Contig13.597   evm.model.Contig4.866          0
 70-  2: evm.model.Contig13.620   evm.model.Contig4.858      4e-59
 70-  3: evm.model.Contig13.625   evm.model.Contig4.853     1e-108
 70-  4: evm.model.Contig13.631   evm.model.Contig4.847          0
 70-  5: evm.model.Contig13.643   evm.model.Contig4.842     1e-121
 70-  6: evm.model.Contig13.646   evm.model.Contig4.836     1e-152
 70-  7: evm.model.Contig13.648   evm.model.Contig4.831     1e-124
 70-  8: evm.model.Contig13.649   evm.model.Contig4.829      6e-92
 70-  9: evm.model.Contig13.652   evm.model.Contig4.828     5e-111
 70- 10: evm.model.Contig13.659   evm.model.Contig4.824     3e-170
 70- 11: evm.model.Contig13.661   evm.model.Contig4.821     3e-154
 70- 12: evm.model.Contig13.662   evm.model.Contig4.819     4e-132
## Alignment 71: score=371.0 e_value=1.4e-14 N=8 ew13&ew4 minus
 71-  0: evm.model.Contig13.77    evm.model.Contig4.812          0
 71-  1: evm.model.Contig13.86    evm.model.Contig4.809      4e-12
 71-  2: evm.model.Contig13.92    evm.model.Contig4.805          0
 71-  3: evm.model.Contig13.96    evm.model.Contig4.798          0
 71-  4: evm.model.Contig13.98    evm.model.Contig4.797      2e-69
 71-  5: evm.model.Contig13.99    evm.model.Contig4.795     1e-126
 71-  6: evm.model.Contig13.101   evm.model.Contig4.788      5e-35
 71-  7: evm.model.Contig13.104   evm.model.Contig4.785     6e-115
## Alignment 72: score=283.0 e_value=4.8e-10 N=6 ew13&ew4 minus
```

```
 72-  0: evm.model.Contig13.451   evm.model.Contig4.340          0
 72-  1: evm.model.Contig13.458   evm.model.Contig4.339     7e-144
 72-  2: evm.model.Contig13.465   evm.model.Contig4.333     1e-179
 72-  3: evm.model.Contig13.467   evm.model.Contig4.327     3e-144
 72-  4: evm.model.Contig13.468   evm.model.Contig4.326     5e-173
 72-  5: evm.model.Contig13.469   evm.model.Contig4.325          0
## Alignment 73: score=308.0 e_value=1e-14 N=7 ew14&ew14 plus
 73-  0: evm.model.Contig14.480   evm.model.Contig14.681      2e-56
 73-  1: evm.model.Contig14.481   evm.model.Contig14.682      7e-66
 73-  2: evm.model.Contig14.482   evm.model.Contig14.683      2e-74
 73-  3: evm.model.Contig14.493   evm.model.Contig14.689          0
 73-  4: evm.model.Contig14.499   evm.model.Contig14.693      5e-93
 73-  5: evm.model.Contig14.510   evm.model.Contig14.694     5e-119
 73-  6: evm.model.Contig14.528   evm.model.Contig14.712     2e-143
## Alignment 74: score=1646.0 e_value=8.1e-130 N=37 ew14&ew27 plus
 74-  0: evm.model.Contig14.271   evm.model.Contig27.239          0
 74-  1: evm.model.Contig14.279   evm.model.Contig27.245      3e-54
 74-  2: evm.model.Contig14.304   evm.model.Contig27.259          0
 74-  3: evm.model.Contig14.305   evm.model.Contig27.268     7e-144
 74-  4: evm.model.Contig14.308   evm.model.Contig27.276      6e-14
 74-  5: evm.model.Contig14.313   evm.model.Contig27.288     5e-142
 74-  6: evm.model.Contig14.314   evm.model.Contig27.289          0
 74-  7: evm.model.Contig14.319   evm.model.Contig27.292          0
 74-  8: evm.model.Contig14.322   evm.model.Contig27.296          0
 74-  9: evm.model.Contig14.324   evm.model.Contig27.299      3e-62
 74- 10: evm.model.Contig14.325   evm.model.Contig27.303     2e-125
 74- 11: evm.model.Contig14.326   evm.model.Contig27.305      6e-95
 74- 12: evm.model.Contig14.328   evm.model.Contig27.307     7e-142
 74- 13: evm.model.Contig14.329   evm.model.Contig27.308      1e-22
 74- 14: evm.model.Contig14.330   evm.model.Contig27.313          0
 74- 15: evm.model.Contig14.342   evm.model.Contig27.318          0
 74- 16: evm.model.Contig14.347   evm.model.Contig27.326      7e-21
 74- 17: evm.model.Contig14.355   evm.model.Contig27.335      4e-48
 74- 18: evm.model.Contig14.372   evm.model.Contig27.336     1e-164
 74- 19: evm.model.Contig14.374   evm.model.Contig27.338          0
 74- 20: evm.model.Contig14.376   evm.model.Contig27.339      6e-19
 74- 21: evm.model.Contig14.382   evm.model.Contig27.349      6e-25
 74- 22: evm.model.Contig14.383   evm.model.Contig27.350      4e-42
 74- 23: evm.model.Contig14.384   evm.model.Contig27.351      3e-60
 74- 24: evm.model.Contig14.386   evm.model.Contig27.355      3e-73
 74- 25: evm.model.Contig14.398   evm.model.Contig27.359      6e-35
 74- 26: evm.model.Contig14.400   evm.model.Contig27.360      3e-43
 74- 27: evm.model.Contig14.407   evm.model.Contig27.363          0
 74- 28: evm.model.Contig14.408   evm.model.Contig27.369      4e-65
 74- 29: evm.model.Contig14.410   evm.model.Contig27.370      1e-65
 74- 30: evm.model.Contig14.415   evm.model.Contig27.372      4e-38
 74- 31: evm.model.Contig14.417   evm.model.Contig27.374      3e-67
 74- 32: evm.model.Contig14.423   evm.model.Contig27.377      9e-39
 74- 33: evm.model.Contig14.426   evm.model.Contig27.378      2e-47
 74- 34: evm.model.Contig14.433   evm.model.Contig27.383          0
 74- 35: evm.model.Contig14.437   evm.model.Contig27.393     6e-110
 74- 36: evm.model.Contig14.455   evm.model.Contig27.416      1e-84
## Alignment 75: score=806.0 e_value=8.6e-49 N=18 ew14&ew27 plus
 75-  0: evm.model.Contig14.113   evm.model.Contig27.68       2e-25
```

```
 75-  1: evm.model.Contig14.118   evm.model.Contig27.72      1e-20
 75-  2: evm.model.Contig14.124   evm.model.Contig27.77     2e-155
 75-  3: evm.model.Contig14.125   evm.model.Contig27.79          0
 75-  4: evm.model.Contig14.132   evm.model.Contig27.81     2e-158
 75-  5: evm.model.Contig14.133   evm.model.Contig27.84          0
 75-  6: evm.model.Contig14.141   evm.model.Contig27.86      2e-80
 75-  7: evm.model.Contig14.145   evm.model.Contig27.88     2e-106
 75-  8: evm.model.Contig14.154   evm.model.Contig27.92      2e-93
 75-  9: evm.model.Contig14.170   evm.model.Contig27.96          0
 75- 10: evm.model.Contig14.172   evm.model.Contig27.97      1e-67
 75- 11: evm.model.Contig14.185   evm.model.Contig27.112    8e-125
 75- 12: evm.model.Contig14.190   evm.model.Contig27.116         0
 75- 13: evm.model.Contig14.192   evm.model.Contig27.124     3e-56
 75- 14: evm.model.Contig14.195   evm.model.Contig27.127         0
 75- 15: evm.model.Contig14.196   evm.model.Contig27.129         0
 75- 16: evm.model.Contig14.202   evm.model.Contig27.137     2e-66
 75- 17: evm.model.Contig14.210   evm.model.Contig27.143         0
## Alignment 76: score=386.0 e_value=4.5e-14 N=8 ew14&ew27 plus
 76-  0: evm.model.Contig14.3     evm.model.Contig27.161         0
 76-  1: evm.model.Contig14.4     evm.model.Contig27.167     4e-69
 76-  2: evm.model.Contig14.6     evm.model.Contig27.175     8e-42
 76-  3: evm.model.Contig14.7     evm.model.Contig27.176         0
 76-  4: evm.model.Contig14.8     evm.model.Contig27.178         0
 76-  5: evm.model.Contig14.9     evm.model.Contig27.179     4e-67
 76-  6: evm.model.Contig14.10    evm.model.Contig27.180     1e-87
 76-  7: evm.model.Contig14.11    evm.model.Contig27.182         0
## Alignment 77: score=371.0 e_value=4.3e-17 N=8 ew14&ew27 plus
 77-  0: evm.model.Contig14.24    evm.model.Contig27.38      7e-47
 77-  1: evm.model.Contig14.37    evm.model.Contig27.47     3e-117
 77-  2: evm.model.Contig14.43    evm.model.Contig27.52          0
 77-  3: evm.model.Contig14.50    evm.model.Contig27.56      5e-20
 77-  4: evm.model.Contig14.51    evm.model.Contig27.57          0
 77-  5: evm.model.Contig14.52    evm.model.Contig27.58     2e-125
 77-  6: evm.model.Contig14.58    evm.model.Contig27.63     6e-152
 77-  7: evm.model.Contig14.59    evm.model.Contig27.65     2e-100
## Alignment 78: score=326.0 e_value=4.5e-16 N=8 ew14&ew27 plus
 78-  0: evm.model.Contig14.516   evm.model.Contig27.504         0
 78-  1: evm.model.Contig14.518   evm.model.Contig27.506         0
 78-  2: evm.model.Contig14.522   evm.model.Contig27.518    6e-112
 78-  3: evm.model.Contig14.523   evm.model.Contig27.519     1e-84
 78-  4: evm.model.Contig14.524   evm.model.Contig27.530     2e-56
 78-  5: evm.model.Contig14.531   evm.model.Contig27.543         0
 78-  6: evm.model.Contig14.542   evm.model.Contig27.560         0
 78-  7: evm.model.Contig14.567   evm.model.Contig27.564     1e-35
## Alignment 79: score=321.0 e_value=4.5e-15 N=8 ew14&ew27 plus
 79-  0: evm.model.Contig14.335   evm.model.Contig27.322     4e-43
 79-  1: evm.model.Contig14.360   evm.model.Contig27.337     4e-65
 79-  2: evm.model.Contig14.375   evm.model.Contig27.346     2e-69
 79-  3: evm.model.Contig14.393   evm.model.Contig27.349     3e-25
 79-  4: evm.model.Contig14.396   evm.model.Contig27.351         0
 79-  5: evm.model.Contig14.397   evm.model.Contig27.352    3e-104
 79-  6: evm.model.Contig14.420   evm.model.Contig27.358     3e-14
 79-  7: evm.model.Contig14.421   evm.model.Contig27.359     2e-35
## Alignment 80: score=409.0 e_value=1.3e-17 N=9 ew14&ew27 minus
```

```
 80-  0: evm.model.Contig14.258   evm.model.Contig27.255        0
 80-  1: evm.model.Contig14.263   evm.model.Contig27.247   2e-125
 80-  2: evm.model.Contig14.264   evm.model.Contig27.246   1e-159
 80-  3: evm.model.Contig14.268   evm.model.Contig27.245    3e-52
 80-  4: evm.model.Contig14.270   evm.model.Contig27.241   1e-107
 80-  5: evm.model.Contig14.281   evm.model.Contig27.233    1e-51
 80-  6: evm.model.Contig14.283   evm.model.Contig27.232    3e-19
 80-  7: evm.model.Contig14.284   evm.model.Contig27.229        0
 80-  8: evm.model.Contig14.300   evm.model.Contig27.225    6e-34
## Alignment 81: score=321.0 e_value=3.1e-11 N=7 ew14&ew27 minus
 81-  0: evm.model.Contig14.724   evm.model.Contig27.484        0
 81-  1: evm.model.Contig14.726   evm.model.Contig27.483   4e-147
 81-  2: evm.model.Contig14.730   evm.model.Contig27.480    2e-34
 81-  3: evm.model.Contig14.733   evm.model.Contig27.478    3e-21
 81-  4: evm.model.Contig14.739   evm.model.Contig27.464        0
 81-  5: evm.model.Contig14.741   evm.model.Contig27.463        0
 81-  6: evm.model.Contig14.751   evm.model.Contig27.455   3e-115
## Alignment 82: score=318.0 e_value=8e-17 N=8 ew14&ew27 minus
 82-  0: evm.model.Contig14.34    evm.model.Contig27.52         0
 82-  1: evm.model.Contig14.38    evm.model.Contig27.45         0
 82-  2: evm.model.Contig14.50    evm.model.Contig27.25     8e-21
 82-  3: evm.model.Contig14.51    evm.model.Contig27.24    1e-169
 82-  4: evm.model.Contig14.52    evm.model.Contig27.21     3e-87
 82-  5: evm.model.Contig14.71    evm.model.Contig27.19     3e-81
 82-  6: evm.model.Contig14.94    evm.model.Contig27.4      1e-24
 82-  7: evm.model.Contig14.110   evm.model.Contig27.2      7e-42
## Alignment 83: score=266.0 e_value=6.6e-08 N=6 ew14&ew27 minus
 83-  0: evm.model.Contig14.229   evm.model.Contig27.220    2e-64
 83-  1: evm.model.Contig14.231   evm.model.Contig27.215    3e-76
 83-  2: evm.model.Contig14.236   evm.model.Contig27.210    6e-60
 83-  3: evm.model.Contig14.238   evm.model.Contig27.200   1e-119
 83-  4: evm.model.Contig14.246   evm.model.Contig27.193    3e-47
 83-  5: evm.model.Contig14.251   evm.model.Contig27.182    2e-98
## Alignment 84: score=302.0 e_value=2.1e-13 N=7 ew15&ew15 plus
 84-  0: evm.model.Contig15.246   evm.model.Contig15.290    8e-92
 84-  1: evm.model.Contig15.248   evm.model.Contig15.311    8e-28
 84-  2: evm.model.Contig15.249   evm.model.Contig15.316    2e-50
 84-  3: evm.model.Contig15.261   evm.model.Contig15.320        0
 84-  4: evm.model.Contig15.268   evm.model.Contig15.321        0
 84-  5: evm.model.Contig15.274   evm.model.Contig15.329    3e-15
 84-  6: evm.model.Contig15.275   evm.model.Contig15.330    8e-18
## Alignment 85: score=478.0 e_value=2.5e-26 N=11 ew15&ew26 plus
 85-  0: evm.model.Contig15.147   evm.model.Contig26.79    7e-111
 85-  1: evm.model.Contig15.158   evm.model.Contig26.102    1e-16
 85-  2: evm.model.Contig15.165   evm.model.Contig26.118        0
 85-  3: evm.model.Contig15.170   evm.model.Contig26.122    7e-66
 85-  4: evm.model.Contig15.172   evm.model.Contig26.123        0
 85-  5: evm.model.Contig15.175   evm.model.Contig26.124        0
 85-  6: evm.model.Contig15.189   evm.model.Contig26.127   4e-121
 85-  7: evm.model.Contig15.190   evm.model.Contig26.129    4e-57
 85-  8: evm.model.Contig15.193   evm.model.Contig26.133    1e-49
 85-  9: evm.model.Contig15.194   evm.model.Contig26.134   2e-140
 85- 10: evm.model.Contig15.199   evm.model.Contig26.146    1e-51
## Alignment 86: score=589.0 e_value=1.4e-33 N=14 ew15&ew26 minus
```

```
 86-  0: evm.model.Contig15.343   evm.model.Contig26.65      2e-61
 86-  1: evm.model.Contig15.344   evm.model.Contig26.63     5e-113
 86-  2: evm.model.Contig15.347   evm.model.Contig26.59      4e-45
 86-  3: evm.model.Contig15.352   evm.model.Contig26.57     2e-125
 86-  4: evm.model.Contig15.370   evm.model.Contig26.54      4e-45
 86-  5: evm.model.Contig15.395   evm.model.Contig26.46      3e-54
 86-  6: evm.model.Contig15.400   evm.model.Contig26.40          0
 86-  7: evm.model.Contig15.401   evm.model.Contig26.37          0
 86-  8: evm.model.Contig15.407   evm.model.Contig26.32          0
 86-  9: evm.model.Contig15.417   evm.model.Contig26.28     1e-173
 86- 10: evm.model.Contig15.437   evm.model.Contig26.23          0
 86- 11: evm.model.Contig15.450   evm.model.Contig26.15     2e-122
 86- 12: evm.model.Contig15.455   evm.model.Contig26.8       8e-16
 86- 13: evm.model.Contig15.459   evm.model.Contig26.3      2e-135
## Alignment 87: score=313.0 e_value=1.3e-12 N=7 ew15&ew26 minus
 87-  0: evm.model.Contig15.206   evm.model.Contig26.277         0
 87-  1: evm.model.Contig15.207   evm.model.Contig26.275         0
 87-  2: evm.model.Contig15.212   evm.model.Contig26.268    2e-127
 87-  3: evm.model.Contig15.215   evm.model.Contig26.265     2e-47
 87-  4: evm.model.Contig15.227   evm.model.Contig26.264         0
 87-  5: evm.model.Contig15.230   evm.model.Contig26.252         0
 87-  6: evm.model.Contig15.234   evm.model.Contig26.245     1e-44
## Alignment 88: score=312.0 e_value=2.3e-12 N=7 ew15&ew26 minus
 88-  0: evm.model.Contig15.76    evm.model.Contig26.225    1e-102
 88-  1: evm.model.Contig15.82    evm.model.Contig26.219     2e-34
 88-  2: evm.model.Contig15.90    evm.model.Contig26.211     4e-16
 88-  3: evm.model.Contig15.92    evm.model.Contig26.210    3e-155
 88-  4: evm.model.Contig15.110   evm.model.Contig26.200     2e-49
 88-  5: evm.model.Contig15.111   evm.model.Contig26.194         0
 88-  6: evm.model.Contig15.115   evm.model.Contig26.193     2e-37
## Alignment 89: score=269.0 e_value=5.7e-15 N=8 ew15&ew26 minus
 89-  0: evm.model.Contig15.66    evm.model.Contig26.172     2e-46
 89-  1: evm.model.Contig15.87    evm.model.Contig26.171         0
 89-  2: evm.model.Contig15.107   evm.model.Contig26.169     3e-83
 89-  3: evm.model.Contig15.108   evm.model.Contig26.161    2e-102
 89-  4: evm.model.Contig15.132   evm.model.Contig26.160     1e-34
 89-  5: evm.model.Contig15.157   evm.model.Contig26.150     7e-44
 89-  6: evm.model.Contig15.175   evm.model.Contig26.124         0
 89-  7: evm.model.Contig15.188   evm.model.Contig26.110     2e-34
## Alignment 90: score=1250.0 e_value=3.4e-93 N=28 ew16&ew25 plus
 90-  0: evm.model.Contig16.122   evm.model.Contig25.156     8e-36
 90-  1: evm.model.Contig16.127   evm.model.Contig25.165     8e-69
 90-  2: evm.model.Contig16.130   evm.model.Contig25.173    2e-159
 90-  3: evm.model.Contig16.139   evm.model.Contig25.174         0
 90-  4: evm.model.Contig16.143   evm.model.Contig25.177     1e-82
 90-  5: evm.model.Contig16.147   evm.model.Contig25.181     5e-33
 90-  6: evm.model.Contig16.149   evm.model.Contig25.186    4e-174
 90-  7: evm.model.Contig16.150   evm.model.Contig25.187     2e-86
 90-  8: evm.model.Contig16.156   evm.model.Contig25.193     6e-39
 90-  9: evm.model.Contig16.159   evm.model.Contig25.198     1e-68
 90- 10: evm.model.Contig16.160   evm.model.Contig25.200     1e-37
 90- 11: evm.model.Contig16.161   evm.model.Contig25.201     8e-85
 90- 12: evm.model.Contig16.163   evm.model.Contig25.202    2e-131
 90- 13: evm.model.Contig16.170   evm.model.Contig25.203         0
```

```
 90- 14: evm.model.Contig16.179   evm.model.Contig25.207        0
 90- 15: evm.model.Contig16.181   evm.model.Contig25.210    1e-75
 90- 16: evm.model.Contig16.189   evm.model.Contig25.211   2e-126
 90- 17: evm.model.Contig16.205   evm.model.Contig25.229    3e-78
 90- 18: evm.model.Contig16.206   evm.model.Contig25.237    1e-93
 90- 19: evm.model.Contig16.209   evm.model.Contig25.239    2e-76
 90- 20: evm.model.Contig16.210   evm.model.Contig25.241    7e-43
 90- 21: evm.model.Contig16.211   evm.model.Contig25.242        0
 90- 22: evm.model.Contig16.212   evm.model.Contig25.245        0
 90- 23: evm.model.Contig16.227   evm.model.Contig25.268   2e-176
 90- 24: evm.model.Contig16.232   evm.model.Contig25.270    7e-53
 90- 25: evm.model.Contig16.236   evm.model.Contig25.272    2e-41
 90- 26: evm.model.Contig16.262   evm.model.Contig25.294    3e-11
 90- 27: evm.model.Contig16.263   evm.model.Contig25.295    1e-34
## Alignment 91: score=575.0 e_value=1.1e-30 N=13 ew16&ew25 plus
 91-  0: evm.model.Contig16.14    evm.model.Contig25.100   1e-148
 91-  1: evm.model.Contig16.31    evm.model.Contig25.101   3e-110
 91-  2: evm.model.Contig16.32    evm.model.Contig25.108        0
 91-  3: evm.model.Contig16.34    evm.model.Contig25.109    6e-52
 91-  4: evm.model.Contig16.38    evm.model.Contig25.119   9e-156
 91-  5: evm.model.Contig16.40    evm.model.Contig25.129    3e-37
 91-  6: evm.model.Contig16.41    evm.model.Contig25.130   7e-103
 91-  7: evm.model.Contig16.46    evm.model.Contig25.135    7e-46
 91-  8: evm.model.Contig16.48    evm.model.Contig25.140   4e-120
 91-  9: evm.model.Contig16.49    evm.model.Contig25.141        0
 91- 10: evm.model.Contig16.51    evm.model.Contig25.144   2e-151
 91- 11: evm.model.Contig16.53    evm.model.Contig25.145    1e-85
 91- 12: evm.model.Contig16.77    evm.model.Contig25.154        0
## Alignment 92: score=367.0 e_value=5.4e-15 N=8 ew16&ew25 plus
 92-  0: evm.model.Contig16.357   evm.model.Contig25.663   1e-104
 92-  1: evm.model.Contig16.359   evm.model.Contig25.665    3e-36
 92-  2: evm.model.Contig16.360   evm.model.Contig25.666    6e-54
 92-  3: evm.model.Contig16.365   evm.model.Contig25.673    1e-21
 92-  4: evm.model.Contig16.367   evm.model.Contig25.676        0
 92-  5: evm.model.Contig16.372   evm.model.Contig25.698   1e-168
 92-  6: evm.model.Contig16.374   evm.model.Contig25.702    6e-24
 92-  7: evm.model.Contig16.375   evm.model.Contig25.703   7e-125
## Alignment 93: score=325.0 e_value=3.5e-17 N=8 ew16&ew25 plus
 93-  0: evm.model.Contig16.173   evm.model.Contig25.222    9e-42
 93-  1: evm.model.Contig16.174   evm.model.Contig25.224    6e-26
 93-  2: evm.model.Contig16.189   evm.model.Contig25.226    2e-65
 93-  3: evm.model.Contig16.200   evm.model.Contig25.250        0
 93-  4: evm.model.Contig16.211   evm.model.Contig25.274        0
 93-  5: evm.model.Contig16.217   evm.model.Contig25.275    6e-59
 93-  6: evm.model.Contig16.218   evm.model.Contig25.277    8e-63
 93-  7: evm.model.Contig16.221   evm.model.Contig25.286        0
## Alignment 94: score=923.0 e_value=8.5e-62 N=21 ew16&ew25 minus
 94-  0: evm.model.Contig16.220   evm.model.Contig25.644        0
 94-  1: evm.model.Contig16.244   evm.model.Contig25.631   2e-140
 94-  2: evm.model.Contig16.270   evm.model.Contig25.626        0
 94-  3: evm.model.Contig16.281   evm.model.Contig25.617   4e-109
 94-  4: evm.model.Contig16.288   evm.model.Contig25.615    2e-90
 94-  5: evm.model.Contig16.291   evm.model.Contig25.613        0
 94-  6: evm.model.Contig16.292   evm.model.Contig25.611    7e-49
```

```
 94-  7: evm.model.Contig16.298   evm.model.Contig25.607         0
 94-  8: evm.model.Contig16.303   evm.model.Contig25.605         0
 94-  9: evm.model.Contig16.310   evm.model.Contig25.602    2e-133
 94- 10: evm.model.Contig16.316   evm.model.Contig25.592    2e-148
 94- 11: evm.model.Contig16.320   evm.model.Contig25.590         0
 94- 12: evm.model.Contig16.322   evm.model.Contig25.588    1e-139
 94- 13: evm.model.Contig16.326   evm.model.Contig25.582    5e-116
 94- 14: evm.model.Contig16.331   evm.model.Contig25.578         0
 94- 15: evm.model.Contig16.354   evm.model.Contig25.575     8e-85
 94- 16: evm.model.Contig16.355   evm.model.Contig25.574         0
 94- 17: evm.model.Contig16.357   evm.model.Contig25.572    3e-102
 94- 18: evm.model.Contig16.358   evm.model.Contig25.571     1e-87
 94- 19: evm.model.Contig16.359   evm.model.Contig25.570     1e-36
 94- 20: evm.model.Contig16.360   evm.model.Contig25.569     2e-44
## Alignment 95: score=441.0 e_value=9.3e-20 N=10 ew16&ew25 minus
 95-  0: evm.model.Contig16.71    evm.model.Contig25.64      7e-53
 95-  1: evm.model.Contig16.79    evm.model.Contig25.50     6e-112
 95-  2: evm.model.Contig16.89    evm.model.Contig25.47          0
 95-  3: evm.model.Contig16.93    evm.model.Contig25.45      4e-29
 95-  4: evm.model.Contig16.98    evm.model.Contig25.37      6e-16
 95-  5: evm.model.Contig16.104   evm.model.Contig25.23      7e-16
 95-  6: evm.model.Contig16.107   evm.model.Contig25.19          0
 95-  7: evm.model.Contig16.111   evm.model.Contig25.12     3e-174
 95-  8: evm.model.Contig16.112   evm.model.Contig25.11      8e-52
 95-  9: evm.model.Contig16.118   evm.model.Contig25.6           0
## Alignment 96: score=437.0 e_value=1.5e-22 N=10 ew16&ew25 minus
 96-  0: evm.model.Contig16.297   evm.model.Contig25.612     8e-55
 96-  1: evm.model.Contig16.309   evm.model.Contig25.605         0
 96-  2: evm.model.Contig16.320   evm.model.Contig25.603         0
 96-  3: evm.model.Contig16.322   evm.model.Contig25.602    6e-132
 96-  4: evm.model.Contig16.329   evm.model.Contig25.586         0
 96-  5: evm.model.Contig16.335   evm.model.Contig25.574    3e-111
 96-  6: evm.model.Contig16.336   evm.model.Contig25.572     5e-99
 96-  7: evm.model.Contig16.337   evm.model.Contig25.571     2e-87
 96-  8: evm.model.Contig16.339   evm.model.Contig25.569     3e-69
 96-  9: evm.model.Contig16.353   evm.model.Contig25.567     5e-12
## Alignment 97: score=324.0 e_value=6.8e-11 N=7 ew16&ew25 minus
 97-  0: evm.model.Contig16.607   evm.model.Contig25.565     5e-33
 97-  1: evm.model.Contig16.609   evm.model.Contig25.563         0
 97-  2: evm.model.Contig16.615   evm.model.Contig25.555     4e-63
 97-  3: evm.model.Contig16.617   evm.model.Contig25.551    3e-151
 97-  4: evm.model.Contig16.620   evm.model.Contig25.545         0
 97-  5: evm.model.Contig16.627   evm.model.Contig25.543     4e-21
 97-  6: evm.model.Contig16.632   evm.model.Contig25.540     2e-83
## Alignment 98: score=426.0 e_value=3.3e-21 N=9 ew17&ew17 minus
 98-  0: evm.model.Contig17.5     evm.model.Contig17.269    3e-136
 98-  1: evm.model.Contig17.6     evm.model.Contig17.268         0
 98-  2: evm.model.Contig17.14    evm.model.Contig17.250    2e-177
 98-  3: evm.model.Contig17.15    evm.model.Contig17.249         0
 98-  4: evm.model.Contig17.16    evm.model.Contig17.247     1e-60
 98-  5: evm.model.Contig17.22    evm.model.Contig17.244         0
 98-  6: evm.model.Contig17.23    evm.model.Contig17.243     1e-55
 98-  7: evm.model.Contig17.24    evm.model.Contig17.242    7e-130
 98-  8: evm.model.Contig17.26    evm.model.Contig17.241    2e-136
```

```
## Alignment 99: score=346.0 e_value=2.1e-10 N=7 ew17&ew17 minus
 99-  0: evm.model.Contig17.670   evm.model.Contig17.689          0
 99-  1: evm.model.Contig17.671   evm.model.Contig17.688          0
 99-  2: evm.model.Contig17.675   evm.model.Contig17.685      3e-88
 99-  3: evm.model.Contig17.676   evm.model.Contig17.683          0
 99-  4: evm.model.Contig17.677   evm.model.Contig17.682          0
 99-  5: evm.model.Contig17.678   evm.model.Contig17.681          0
 99-  6: evm.model.Contig17.679   evm.model.Contig17.680     2e-161
## Alignment 100: score=563.0 e_value=1.4e-28 N=13 ew17&ew22 plus
100-  0: evm.model.Contig17.56    evm.model.Contig22.332          0
100-  1: evm.model.Contig17.62    evm.model.Contig22.338     3e-144
100-  2: evm.model.Contig17.64    evm.model.Contig22.340      1e-20
100-  3: evm.model.Contig17.67    evm.model.Contig22.346     5e-113
100-  4: evm.model.Contig17.68    evm.model.Contig22.356      2e-28
100-  5: evm.model.Contig17.71    evm.model.Contig22.370      4e-77
100-  6: evm.model.Contig17.74    evm.model.Contig22.379     3e-129
100-  7: evm.model.Contig17.75    evm.model.Contig22.381      2e-42
100-  8: evm.model.Contig17.91    evm.model.Contig22.382          0
100-  9: evm.model.Contig17.102   evm.model.Contig22.387      2e-95
100- 10: evm.model.Contig17.104   evm.model.Contig22.394          0
100- 11: evm.model.Contig17.105   evm.model.Contig22.398          0
100- 12: evm.model.Contig17.109   evm.model.Contig22.410      7e-31
## Alignment 101: score=546.0 e_value=9.8e-29 N=12 ew17&ew22 plus
101-  0: evm.model.Contig17.709   evm.model.Contig22.602      4e-62
101-  1: evm.model.Contig17.714   evm.model.Contig22.618      2e-83
101-  2: evm.model.Contig17.717   evm.model.Contig22.632          0
101-  3: evm.model.Contig17.724   evm.model.Contig22.634     1e-130
101-  4: evm.model.Contig17.725   evm.model.Contig22.636      7e-60
101-  5: evm.model.Contig17.726   evm.model.Contig22.637     4e-165
101-  6: evm.model.Contig17.729   evm.model.Contig22.640     7e-166
101-  7: evm.model.Contig17.734   evm.model.Contig22.644      9e-41
101-  8: evm.model.Contig17.740   evm.model.Contig22.651      1e-13
101-  9: evm.model.Contig17.741   evm.model.Contig22.653      8e-24
101- 10: evm.model.Contig17.742   evm.model.Contig22.654      4e-22
101- 11: evm.model.Contig17.749   evm.model.Contig22.660          0
## Alignment 102: score=492.0 e_value=7.3e-29 N=11 ew17&ew22 plus
102-  0: evm.model.Contig17.752   evm.model.Contig22.98           0
102-  1: evm.model.Contig17.753   evm.model.Contig22.99           0
102-  2: evm.model.Contig17.754   evm.model.Contig22.102     5e-150
102-  3: evm.model.Contig17.755   evm.model.Contig22.103          0
102-  4: evm.model.Contig17.763   evm.model.Contig22.106          0
102-  5: evm.model.Contig17.769   evm.model.Contig22.107          0
102-  6: evm.model.Contig17.780   evm.model.Contig22.117      1e-57
102-  7: evm.model.Contig17.790   evm.model.Contig22.124          0
102-  8: evm.model.Contig17.791   evm.model.Contig22.150          0
102-  9: evm.model.Contig17.792   evm.model.Contig22.151     3e-153
102- 10: evm.model.Contig17.793   evm.model.Contig22.152      2e-13
## Alignment 103: score=480.0 e_value=6e-20 N=10 ew17&ew22 plus
103-  0: evm.model.Contig17.32    evm.model.Contig22.673          0
103-  1: evm.model.Contig17.33    evm.model.Contig22.674      5e-69
103-  2: evm.model.Contig17.35    evm.model.Contig22.675          0
103-  3: evm.model.Contig17.36    evm.model.Contig22.676          0
103-  4: evm.model.Contig17.37    evm.model.Contig22.679      1e-66
103-  5: evm.model.Contig17.38    evm.model.Contig22.681      2e-77
```

```
103-  6: evm.model.Contig17.41     evm.model.Contig22.683      1e-77
103-  7: evm.model.Contig17.43     evm.model.Contig22.692     6e-179
103-  8: evm.model.Contig17.48     evm.model.Contig22.695     3e-105
103-  9: evm.model.Contig17.51     evm.model.Contig22.696     1e-116
## Alignment 104: score=356.0 e_value=1.4e-15 N=8 ew17&ew22 plus
104-  0: evm.model.Contig17.244    evm.model.Contig22.7        1e-96
104-  1: evm.model.Contig17.261    evm.model.Contig22.26       7e-85
104-  2: evm.model.Contig17.269    evm.model.Contig22.34       2e-22
104-  3: evm.model.Contig17.273    evm.model.Contig22.37      1e-119
104-  4: evm.model.Contig17.274    evm.model.Contig22.39       5e-31
104-  5: evm.model.Contig17.275    evm.model.Contig22.47      1e-135
104-  6: evm.model.Contig17.282    evm.model.Contig22.54           0
104-  7: evm.model.Contig17.285    evm.model.Contig22.56       3e-52
## Alignment 105: score=332.0 e_value=1.6e-11 N=7 ew17&ew22 plus
105-  0: evm.model.Contig17.569    evm.model.Contig22.173      5e-19
105-  1: evm.model.Contig17.573    evm.model.Contig22.177          0
105-  2: evm.model.Contig17.581    evm.model.Contig22.181     1e-173
105-  3: evm.model.Contig17.582    evm.model.Contig22.182     6e-127
105-  4: evm.model.Contig17.584    evm.model.Contig22.183      6e-76
105-  5: evm.model.Contig17.589    evm.model.Contig22.191      1e-72
105-  6: evm.model.Contig17.590    evm.model.Contig22.192      3e-21
## Alignment 106: score=262.0 e_value=7.5e-08 N=6 ew17&ew22 plus
106-  0: evm.model.Contig17.545    evm.model.Contig22.194          0
106-  1: evm.model.Contig17.548    evm.model.Contig22.200     5e-134
106-  2: evm.model.Contig17.555    evm.model.Contig22.215          0
106-  3: evm.model.Contig17.559    evm.model.Contig22.222      7e-45
106-  4: evm.model.Contig17.563    evm.model.Contig22.229      2e-39
106-  5: evm.model.Contig17.564    evm.model.Contig22.237      7e-63
## Alignment 107: score=253.0 e_value=1.5e-09 N=6 ew17&ew22 plus
107-  0: evm.model.Contig17.186    evm.model.Contig22.412          0
107-  1: evm.model.Contig17.203    evm.model.Contig22.413     6e-123
107-  2: evm.model.Contig17.204    evm.model.Contig22.418      1e-44
107-  3: evm.model.Contig17.226    evm.model.Contig22.425     7e-113
107-  4: evm.model.Contig17.229    evm.model.Contig22.427      1e-15
107-  5: evm.model.Contig17.234    evm.model.Contig22.430      8e-43
## Alignment 108: score=1096.0 e_value=4e-74 N=24 ew17&ew22 minus
108-  0: evm.model.Contig17.611    evm.model.Contig22.532      9e-56
108-  1: evm.model.Contig17.613    evm.model.Contig22.530          0
108-  2: evm.model.Contig17.615    evm.model.Contig22.529     3e-169
108-  3: evm.model.Contig17.616    evm.model.Contig22.528          0
108-  4: evm.model.Contig17.619    evm.model.Contig22.502          0
108-  5: evm.model.Contig17.620    evm.model.Contig22.501     9e-130
108-  6: evm.model.Contig17.621    evm.model.Contig22.500          0
108-  7: evm.model.Contig17.625    evm.model.Contig22.498      2e-43
108-  8: evm.model.Contig17.626    evm.model.Contig22.497          0
108-  9: evm.model.Contig17.628    evm.model.Contig22.486          0
108- 10: evm.model.Contig17.630    evm.model.Contig22.485     2e-103
108- 11: evm.model.Contig17.632    evm.model.Contig22.482      2e-35
108- 12: evm.model.Contig17.634    evm.model.Contig22.480      3e-96
108- 13: evm.model.Contig17.638    evm.model.Contig22.479     2e-180
108- 14: evm.model.Contig17.640    evm.model.Contig22.478      8e-32
108- 15: evm.model.Contig17.646    evm.model.Contig22.476      2e-25
108- 16: evm.model.Contig17.650    evm.model.Contig22.473          0
108- 17: evm.model.Contig17.653    evm.model.Contig22.471      5e-36
```

```
108- 18: evm.model.Contig17.656   evm.model.Contig22.465     2e-24
108- 19: evm.model.Contig17.664   evm.model.Contig22.462         0
108- 20: evm.model.Contig17.668   evm.model.Contig22.460         0
108- 21: evm.model.Contig17.670   evm.model.Contig22.459         0
108- 22: evm.model.Contig17.678   evm.model.Contig22.455    3e-177
108- 23: evm.model.Contig17.702   evm.model.Contig22.446     2e-13
## Alignment 109: score=520.0 e_value=4.9e-24 N=11 ew17&ew22 minus
109-  0: evm.model.Contig17.304   evm.model.Contig22.332         0
109-  1: evm.model.Contig17.309   evm.model.Contig22.331    5e-105
109-  2: evm.model.Contig17.311   evm.model.Contig22.326         0
109-  3: evm.model.Contig17.316   evm.model.Contig22.322     3e-21
109-  4: evm.model.Contig17.317   evm.model.Contig22.319    5e-142
109-  5: evm.model.Contig17.320   evm.model.Contig22.317         0
109-  6: evm.model.Contig17.321   evm.model.Contig22.316    2e-138
109-  7: evm.model.Contig17.322   evm.model.Contig22.315    2e-141
109-  8: evm.model.Contig17.325   evm.model.Contig22.314    8e-156
109-  9: evm.model.Contig17.329   evm.model.Contig22.302     2e-47
109- 10: evm.model.Contig17.331   evm.model.Contig22.300         0
## Alignment 110: score=504.0 e_value=4.3e-25 N=11 ew17&ew22 minus
110-  0: evm.model.Contig17.551   evm.model.Contig22.225     3e-36
110-  1: evm.model.Contig17.552   evm.model.Contig22.223    3e-112
110-  2: evm.model.Contig17.553   evm.model.Contig22.218    5e-134
110-  3: evm.model.Contig17.554   evm.model.Contig22.216     6e-72
110-  4: evm.model.Contig17.555   evm.model.Contig22.215         0
110-  5: evm.model.Contig17.560   evm.model.Contig22.214         0
110-  6: evm.model.Contig17.566   evm.model.Contig22.212         0
110-  7: evm.model.Contig17.576   evm.model.Contig22.187    7e-176
110-  8: evm.model.Contig17.582   evm.model.Contig22.182    6e-127
110-  9: evm.model.Contig17.585   evm.model.Contig22.179     2e-82
110- 10: evm.model.Contig17.586   evm.model.Contig22.178         0
## Alignment 111: score=369.0 e_value=5e-18 N=8 ew17&ew22 minus
111-  0: evm.model.Contig17.619   evm.model.Contig22.532    5e-155
111-  1: evm.model.Contig17.621   evm.model.Contig22.530         0
111-  2: evm.model.Contig17.622   evm.model.Contig22.529    6e-174
111-  3: evm.model.Contig17.623   evm.model.Contig22.528         0
111-  4: evm.model.Contig17.624   evm.model.Contig22.526     6e-47
111-  5: evm.model.Contig17.625   evm.model.Contig22.522     7e-45
111-  6: evm.model.Contig17.626   evm.model.Contig22.520         0
111-  7: evm.model.Contig17.639   evm.model.Contig22.494     4e-39
## Alignment 112: score=271.0 e_value=2.7e-09 N=6 ew17&ew22 minus
112-  0: evm.model.Contig17.598   evm.model.Contig22.510     2e-81
112-  1: evm.model.Contig17.602   evm.model.Contig22.508     6e-14
112-  2: evm.model.Contig17.610   evm.model.Contig22.502     3e-58
112-  3: evm.model.Contig17.612   evm.model.Contig22.501    9e-130
112-  4: evm.model.Contig17.613   evm.model.Contig22.500         0
112-  5: evm.model.Contig17.632   evm.model.Contig22.491     2e-35
## Alignment 113: score=255.0 e_value=4.9e-10 N=6 ew17&ew22 minus
113-  0: evm.model.Contig17.251   evm.model.Contig22.44      4e-81
113-  1: evm.model.Contig17.253   evm.model.Contig22.39      4e-24
113-  2: evm.model.Contig17.255   evm.model.Contig22.37     1e-119
113-  3: evm.model.Contig17.269   evm.model.Contig22.34      2e-22
113-  4: evm.model.Contig17.275   evm.model.Contig22.26     9e-136
113-  5: evm.model.Contig17.296   evm.model.Contig22.12      1e-16
## Alignment 114: score=337.0 e_value=1.1e-10 N=7 ew18&ew4 minus
```

```
114-  0: evm.model.Contig18.278   evm.model.Contig4.432      6e-37
114-  1: evm.model.Contig18.279   evm.model.Contig4.427      2e-35
114-  2: evm.model.Contig18.281   evm.model.Contig4.418          0
114-  3: evm.model.Contig18.282   evm.model.Contig4.417          0
114-  4: evm.model.Contig18.283   evm.model.Contig4.416          0
114-  5: evm.model.Contig18.284   evm.model.Contig4.415      1e-16
114-  6: evm.model.Contig18.286   evm.model.Contig4.414          0
## Alignment 115: score=669.0 e_value=1.9e-32 N=14 ew18&ew41 plus
115-  0: evm.model.Contig18.111   evm.model.Contig41.245         0
115-  1: evm.model.Contig18.113   evm.model.Contig41.246         0
115-  2: evm.model.Contig18.114   evm.model.Contig41.247    1e-153
115-  3: evm.model.Contig18.121   evm.model.Contig41.250     1e-62
115-  4: evm.model.Contig18.124   evm.model.Contig41.251         0
115-  5: evm.model.Contig18.125   evm.model.Contig41.252     6e-80
115-  6: evm.model.Contig18.126   evm.model.Contig41.254         0
115-  7: evm.model.Contig18.128   evm.model.Contig41.257         0
115-  8: evm.model.Contig18.129   evm.model.Contig41.262    6e-154
115-  9: evm.model.Contig18.131   evm.model.Contig41.264     5e-15
115- 10: evm.model.Contig18.132   evm.model.Contig41.267     2e-11
115- 11: evm.model.Contig18.140   evm.model.Contig41.269         0
115- 12: evm.model.Contig18.143   evm.model.Contig41.270     9e-25
115- 13: evm.model.Contig18.146   evm.model.Contig41.274     4e-80
## Alignment 116: score=434.0 e_value=2.9e-22 N=10 ew18&ew41 plus
116-  0: evm.model.Contig18.6     evm.model.Contig41.380     1e-12
116-  1: evm.model.Contig18.31    evm.model.Contig41.387    4e-176
116-  2: evm.model.Contig18.40    evm.model.Contig41.392    1e-103
116-  3: evm.model.Contig18.42    evm.model.Contig41.393     9e-45
116-  4: evm.model.Contig18.43    evm.model.Contig41.395     2e-93
116-  5: evm.model.Contig18.44    evm.model.Contig41.396         0
116-  6: evm.model.Contig18.45    evm.model.Contig41.397         0
116-  7: evm.model.Contig18.63    evm.model.Contig41.402     4e-98
116-  8: evm.model.Contig18.64    evm.model.Contig41.405     1e-32
116-  9: evm.model.Contig18.78    evm.model.Contig41.410     3e-41
## Alignment 117: score=363.0 e_value=1.1e-17 N=9 ew18&ew41 plus
117-  0: evm.model.Contig18.133   evm.model.Contig41.166     3e-26
117-  1: evm.model.Contig18.156   evm.model.Contig41.183         0
117-  2: evm.model.Contig18.161   evm.model.Contig41.184     4e-96
117-  3: evm.model.Contig18.176   evm.model.Contig41.200         0
117-  4: evm.model.Contig18.179   evm.model.Contig41.202         0
117-  5: evm.model.Contig18.205   evm.model.Contig41.214         0
117-  6: evm.model.Contig18.215   evm.model.Contig41.225     3e-17
117-  7: evm.model.Contig18.222   evm.model.Contig41.231    1e-100
117-  8: evm.model.Contig18.226   evm.model.Contig41.234    9e-133
## Alignment 118: score=333.0 e_value=1.3e-15 N=8 ew18&ew41 plus
118-  0: evm.model.Contig18.16    evm.model.Contig41.337    1e-168
118-  1: evm.model.Contig18.23    evm.model.Contig41.341     1e-87
118-  2: evm.model.Contig18.25    evm.model.Contig41.344     8e-78
118-  3: evm.model.Contig18.26    evm.model.Contig41.350    3e-170
118-  4: evm.model.Contig18.28    evm.model.Contig41.353         0
118-  5: evm.model.Contig18.50    evm.model.Contig41.369     1e-76
118-  6: evm.model.Contig18.58    evm.model.Contig41.376     3e-29
118-  7: evm.model.Contig18.65    evm.model.Contig41.401     8e-29
## Alignment 119: score=285.0 e_value=5.1e-09 N=6 ew18&ew41 plus
119-  0: evm.model.Contig18.292   evm.model.Contig41.144         0
```

```
119-  1: evm.model.Contig18.293   evm.model.Contig41.145    5e-173
119-  2: evm.model.Contig18.294   evm.model.Contig41.146     9e-65
119-  3: evm.model.Contig18.300   evm.model.Contig41.148         0
119-  4: evm.model.Contig18.302   evm.model.Contig41.150         0
119-  5: evm.model.Contig18.307   evm.model.Contig41.160         0
## Alignment 120: score=257.0 e_value=1.4e-09 N=6 ew18&ew41 plus
120-  0: evm.model.Contig18.325   evm.model.Contig41.79          0
120-  1: evm.model.Contig18.331   evm.model.Contig41.85     1e-147
120-  2: evm.model.Contig18.334   evm.model.Contig41.87          0
120-  3: evm.model.Contig18.354   evm.model.Contig41.93      1e-89
120-  4: evm.model.Contig18.358   evm.model.Contig41.94          0
120-  5: evm.model.Contig18.370   evm.model.Contig41.109    1e-161
## Alignment 121: score=536.0 e_value=3.6e-29 N=12 ew18&ew41 minus
121-  0: evm.model.Contig18.329   evm.model.Contig41.86      8e-84
121-  1: evm.model.Contig18.331   evm.model.Contig41.85     1e-147
121-  2: evm.model.Contig18.346   evm.model.Contig41.74     5e-156
121-  3: evm.model.Contig18.348   evm.model.Contig41.72      2e-78
121-  4: evm.model.Contig18.352   evm.model.Contig41.70      8e-22
121-  5: evm.model.Contig18.357   evm.model.Contig41.69      2e-91
121-  6: evm.model.Contig18.358   evm.model.Contig41.65          0
121-  7: evm.model.Contig18.364   evm.model.Contig41.62          0
121-  8: evm.model.Contig18.367   evm.model.Contig41.43     5e-171
121-  9: evm.model.Contig18.370   evm.model.Contig41.41     4e-163
121- 10: evm.model.Contig18.382   evm.model.Contig41.36     2e-170
121- 11: evm.model.Contig18.383   evm.model.Contig41.33      3e-52
## Alignment 122: score=363.0 e_value=2e-15 N=8 ew18&ew41 minus
122-  0: evm.model.Contig18.524   evm.model.Contig41.449     1e-64
122-  1: evm.model.Contig18.526   evm.model.Contig41.448     1e-42
122-  2: evm.model.Contig18.535   evm.model.Contig41.443     1e-37
122-  3: evm.model.Contig18.540   evm.model.Contig41.438         0
122-  4: evm.model.Contig18.553   evm.model.Contig41.435         0
122-  5: evm.model.Contig18.558   evm.model.Contig41.426     2e-89
122-  6: evm.model.Contig18.559   evm.model.Contig41.423     6e-57
122-  7: evm.model.Contig18.562   evm.model.Contig41.422     3e-62
## Alignment 123: score=351.0 e_value=5.7e-15 N=8 ew18&ew41 minus
123-  0: evm.model.Contig18.225   evm.model.Contig41.328     1e-47
123-  1: evm.model.Contig18.238   evm.model.Contig41.307     1e-52
123-  2: evm.model.Contig18.245   evm.model.Contig41.296    5e-158
123-  3: evm.model.Contig18.251   evm.model.Contig41.286     8e-52
123-  4: evm.model.Contig18.253   evm.model.Contig41.282    4e-138
123-  5: evm.model.Contig18.254   evm.model.Contig41.281    5e-164
123-  6: evm.model.Contig18.258   evm.model.Contig41.278     6e-55
123-  7: evm.model.Contig18.263   evm.model.Contig41.275    3e-176
## Alignment 124: score=301.0 e_value=1.4e-12 N=7 ew18&ew41 minus
124-  0: evm.model.Contig18.170   evm.model.Contig41.212         0
124-  1: evm.model.Contig18.172   evm.model.Contig41.205     1e-43
124-  2: evm.model.Contig18.183   evm.model.Contig41.198         0
124-  3: evm.model.Contig18.186   evm.model.Contig41.197     4e-25
124-  4: evm.model.Contig18.198   evm.model.Contig41.192         0
124-  5: evm.model.Contig18.202   evm.model.Contig41.191         0
124-  6: evm.model.Contig18.205   evm.model.Contig41.173    3e-174
## Alignment 125: score=278.0 e_value=7.9e-09 N=6 ew18&ew41 minus
125-  0: evm.model.Contig18.293   evm.model.Contig41.156    1e-126
125-  1: evm.model.Contig18.294   evm.model.Contig41.155         0
```

```
125-  2: evm.model.Contig18.302   evm.model.Contig41.150         0
125-  3: evm.model.Contig18.313   evm.model.Contig41.149     7e-58
125-  4: evm.model.Contig18.315   evm.model.Contig41.143     2e-58
125-  5: evm.model.Contig18.316   evm.model.Contig41.142    6e-124
## Alignment 126: score=266.0 e_value=7.1e-08 N=6 ew18&ew41 minus
126-  0: evm.model.Contig18.38    evm.model.Contig41.387    9e-162
126-  1: evm.model.Contig18.46    evm.model.Contig41.380     2e-33
126-  2: evm.model.Contig18.58    evm.model.Contig41.376     3e-29
126-  3: evm.model.Contig18.59    evm.model.Contig41.369     2e-56
126-  4: evm.model.Contig18.65    evm.model.Contig41.367     4e-22
126-  5: evm.model.Contig18.71    evm.model.Contig41.362     2e-34
## Alignment 127: score=739.0 e_value=1.4e-40 N=16 ew19&ew29 plus
127-  0: evm.model.Contig19.435   evm.model.Contig29.16      3e-79
127-  1: evm.model.Contig19.437   evm.model.Contig29.24      9e-93
127-  2: evm.model.Contig19.440   evm.model.Contig29.39      6e-74
127-  3: evm.model.Contig19.442   evm.model.Contig29.40     9e-125
127-  4: evm.model.Contig19.443   evm.model.Contig29.45      2e-11
127-  5: evm.model.Contig19.447   evm.model.Contig29.49      1e-55
127-  6: evm.model.Contig19.450   evm.model.Contig29.51          0
127-  7: evm.model.Contig19.452   evm.model.Contig29.52          0
127-  8: evm.model.Contig19.459   evm.model.Contig29.54      2e-36
127-  9: evm.model.Contig19.460   evm.model.Contig29.56     5e-146
127- 10: evm.model.Contig19.461   evm.model.Contig29.58          0
127- 11: evm.model.Contig19.463   evm.model.Contig29.61      4e-72
127- 12: evm.model.Contig19.469   evm.model.Contig29.67      4e-27
127- 13: evm.model.Contig19.470   evm.model.Contig29.72          0
127- 14: evm.model.Contig19.471   evm.model.Contig29.74      8e-53
127- 15: evm.model.Contig19.481   evm.model.Contig29.84      8e-28
## Alignment 128: score=462.0 e_value=7e-28 N=11 ew19&ew29 plus
128-  0: evm.model.Contig19.32    evm.model.Contig29.440         0
128-  1: evm.model.Contig19.34    evm.model.Contig29.441         0
128-  2: evm.model.Contig19.35    evm.model.Contig29.442         0
128-  3: evm.model.Contig19.50    evm.model.Contig29.451     4e-57
128-  4: evm.model.Contig19.53    evm.model.Contig29.454    3e-177
128-  5: evm.model.Contig19.76    evm.model.Contig29.458    3e-151
128-  6: evm.model.Contig19.91    evm.model.Contig29.465         0
128-  7: evm.model.Contig19.100   evm.model.Contig29.479    6e-147
128-  8: evm.model.Contig19.105   evm.model.Contig29.480         0
128-  9: evm.model.Contig19.106   evm.model.Contig29.481     5e-64
128- 10: evm.model.Contig19.107   evm.model.Contig29.500     5e-87
## Alignment 129: score=2012.0 e_value=4.2e-173 N=47 ew19&ew29 minus
129-  0: evm.model.Contig19.174   evm.model.Contig29.417     2e-17
129-  1: evm.model.Contig19.179   evm.model.Contig29.401         0
129-  2: evm.model.Contig19.186   evm.model.Contig29.397    8e-113
129-  3: evm.model.Contig19.192   evm.model.Contig29.395    2e-154
129-  4: evm.model.Contig19.205   evm.model.Contig29.382     8e-65
129-  5: evm.model.Contig19.216   evm.model.Contig29.356    2e-104
129-  6: evm.model.Contig19.226   evm.model.Contig29.345         0
129-  7: evm.model.Contig19.227   evm.model.Contig29.337     3e-45
129-  8: evm.model.Contig19.230   evm.model.Contig29.335     2e-64
129-  9: evm.model.Contig19.231   evm.model.Contig29.330         0
129- 10: evm.model.Contig19.233   evm.model.Contig29.326     3e-72
129- 11: evm.model.Contig19.236   evm.model.Contig29.323     2e-27
129- 12: evm.model.Contig19.240   evm.model.Contig29.321     3e-19
```

```
129- 13: evm.model.Contig19.241   evm.model.Contig29.320    9e-106
129- 14: evm.model.Contig19.242   evm.model.Contig29.319     1e-49
129- 15: evm.model.Contig19.243   evm.model.Contig29.318     9e-16
129- 16: evm.model.Contig19.250   evm.model.Contig29.317         0
129- 17: evm.model.Contig19.253   evm.model.Contig29.316     1e-40
129- 18: evm.model.Contig19.261   evm.model.Contig29.308     2e-61
129- 19: evm.model.Contig19.262   evm.model.Contig29.306     4e-21
129- 20: evm.model.Contig19.268   evm.model.Contig29.302         0
129- 21: evm.model.Contig19.278   evm.model.Contig29.294     2e-73
129- 22: evm.model.Contig19.281   evm.model.Contig29.270     9e-41
129- 23: evm.model.Contig19.282   evm.model.Contig29.262     7e-46
129- 24: evm.model.Contig19.286   evm.model.Contig29.255     1e-30
129- 25: evm.model.Contig19.288   evm.model.Contig29.248    1e-113
129- 26: evm.model.Contig19.297   evm.model.Contig29.240    5e-107
129- 27: evm.model.Contig19.307   evm.model.Contig29.218    6e-152
129- 28: evm.model.Contig19.309   evm.model.Contig29.215    4e-109
129- 29: evm.model.Contig19.313   evm.model.Contig29.211         0
129- 30: evm.model.Contig19.316   evm.model.Contig29.208     3e-86
129- 31: evm.model.Contig19.317   evm.model.Contig29.206     4e-17
129- 32: evm.model.Contig19.319   evm.model.Contig29.201         0
129- 33: evm.model.Contig19.325   evm.model.Contig29.196     4e-93
129- 34: evm.model.Contig19.328   evm.model.Contig29.192         0
129- 35: evm.model.Contig19.329   evm.model.Contig29.191         0
129- 36: evm.model.Contig19.331   evm.model.Contig29.182    3e-122
129- 37: evm.model.Contig19.336   evm.model.Contig29.172     4e-62
129- 38: evm.model.Contig19.339   evm.model.Contig29.165         0
129- 39: evm.model.Contig19.342   evm.model.Contig29.162     1e-54
129- 40: evm.model.Contig19.361   evm.model.Contig29.152    3e-141
129- 41: evm.model.Contig19.362   evm.model.Contig29.132     3e-76
129- 42: evm.model.Contig19.364   evm.model.Contig29.124     4e-25
129- 43: evm.model.Contig19.376   evm.model.Contig29.120     9e-98
129- 44: evm.model.Contig19.394   evm.model.Contig29.113         0
129- 45: evm.model.Contig19.407   evm.model.Contig29.112     1e-88
129- 46: evm.model.Contig19.422   evm.model.Contig29.110     2e-12
## Alignment 130: score=363.0 e_value=2.7e-16 N=8 ew19&ew29 minus
130-  0: evm.model.Contig19.154   evm.model.Contig29.377    1e-103
130-  1: evm.model.Contig19.155   evm.model.Contig29.372         0
130-  2: evm.model.Contig19.160   evm.model.Contig29.370     3e-90
130-  3: evm.model.Contig19.167   evm.model.Contig29.368     9e-32
130-  4: evm.model.Contig19.168   evm.model.Contig29.365     4e-13
130-  5: evm.model.Contig19.169   evm.model.Contig29.363     4e-94
130-  6: evm.model.Contig19.170   evm.model.Contig29.361     1e-26
130-  7: evm.model.Contig19.190   evm.model.Contig29.355     9e-52
## Alignment 131: score=311.0 e_value=2.3e-13 N=7 ew19&ew29 minus
131-  0: evm.model.Contig19.314   evm.model.Contig29.218         0
131-  1: evm.model.Contig19.315   evm.model.Contig29.217         0
131-  2: evm.model.Contig19.316   evm.model.Contig29.215     5e-86
131-  3: evm.model.Contig19.317   evm.model.Contig29.213     4e-17
131-  4: evm.model.Contig19.319   evm.model.Contig29.210         0
131-  5: evm.model.Contig19.326   evm.model.Contig29.197     2e-88
131-  6: evm.model.Contig19.339   evm.model.Contig29.173         0
## Alignment 132: score=295.0 e_value=1.1e-11 N=7 ew19&ew29 minus
132-  0: evm.model.Contig19.28    evm.model.Contig29.474         0
132-  1: evm.model.Contig19.31    evm.model.Contig29.472    2e-153
```

```
132-  2: evm.model.Contig19.44    evm.model.Contig29.469     3e-14
132-  3: evm.model.Contig19.67    evm.model.Contig29.462         0
132-  4: evm.model.Contig19.71    evm.model.Contig29.461    4e-100
132-  5: evm.model.Contig19.82    evm.model.Contig29.445     4e-20
132-  6: evm.model.Contig19.84    evm.model.Contig29.444     2e-44
## Alignment 133: score=391.0 e_value=8.3e-19 N=9 ew2&ew2 minus
133-  0: evm.model.Contig2.58     evm.model.Contig2.173      2e-95
133-  1: evm.model.Contig2.59     evm.model.Contig2.172      4e-74
133-  2: evm.model.Contig2.60     evm.model.Contig2.171     6e-175
133-  3: evm.model.Contig2.63     evm.model.Contig2.169      1e-46
133-  4: evm.model.Contig2.82     evm.model.Contig2.146          0
133-  5: evm.model.Contig2.89     evm.model.Contig2.139     2e-112
133-  6: evm.model.Contig2.92     evm.model.Contig2.137      2e-42
133-  7: evm.model.Contig2.95     evm.model.Contig2.120     7e-150
133-  8: evm.model.Contig2.99     evm.model.Contig2.108      6e-75
## Alignment 134: score=281.0 e_value=8.4e-10 N=6 ew2&ew2 minus
134-  0: evm.model.Contig2.340    evm.model.Contig2.385      9e-51
134-  1: evm.model.Contig2.341    evm.model.Contig2.383     1e-167
134-  2: evm.model.Contig2.354    evm.model.Contig2.369     6e-109
134-  3: evm.model.Contig2.355    evm.model.Contig2.368          0
134-  4: evm.model.Contig2.359    evm.model.Contig2.362     1e-123
134-  5: evm.model.Contig2.360    evm.model.Contig2.361          0
## Alignment 135: score=464.0 e_value=6.3e-23 N=10 ew20&ew20 minus
135-  0: evm.model.Contig20.673   evm.model.Contig20.756         0
135-  1: evm.model.Contig20.684   evm.model.Contig20.753         0
135-  2: evm.model.Contig20.688   evm.model.Contig20.751         0
135-  3: evm.model.Contig20.689   evm.model.Contig20.750         0
135-  4: evm.model.Contig20.690   evm.model.Contig20.748     6e-44
135-  5: evm.model.Contig20.710   evm.model.Contig20.727         0
135-  6: evm.model.Contig20.711   evm.model.Contig20.726         0
135-  7: evm.model.Contig20.712   evm.model.Contig20.725         0
135-  8: evm.model.Contig20.713   evm.model.Contig20.724         0
135-  9: evm.model.Contig20.716   evm.model.Contig20.723         0
## Alignment 136: score=743.0 e_value=4.3e-41 N=16 ew20&ew28 plus
136-  0: evm.model.Contig20.108   evm.model.Contig28.591     2e-62
136-  1: evm.model.Contig20.110   evm.model.Contig28.592         0
136-  2: evm.model.Contig20.112   evm.model.Contig28.596         0
136-  3: evm.model.Contig20.113   evm.model.Contig28.597     2e-16
136-  4: evm.model.Contig20.125   evm.model.Contig28.600     2e-26
136-  5: evm.model.Contig20.126   evm.model.Contig28.603    1e-122
136-  6: evm.model.Contig20.130   evm.model.Contig28.604     7e-39
136-  7: evm.model.Contig20.131   evm.model.Contig28.605    2e-180
136-  8: evm.model.Contig20.134   evm.model.Contig28.611     6e-56
136-  9: evm.model.Contig20.139   evm.model.Contig28.612     1e-78
136- 10: evm.model.Contig20.142   evm.model.Contig28.614     5e-48
136- 11: evm.model.Contig20.146   evm.model.Contig28.615     2e-90
136- 12: evm.model.Contig20.149   evm.model.Contig28.617         0
136- 13: evm.model.Contig20.151   evm.model.Contig28.620     3e-62
136- 14: evm.model.Contig20.158   evm.model.Contig28.634         0
136- 15: evm.model.Contig20.165   evm.model.Contig28.641    2e-180
## Alignment 137: score=442.0 e_value=2.4e-21 N=10 ew20&ew28 plus
137-  0: evm.model.Contig20.703   evm.model.Contig28.9           0
137-  1: evm.model.Contig20.722   evm.model.Contig28.10     4e-115
137-  2: evm.model.Contig20.730   evm.model.Contig28.18     1e-180
```

```
137-  3: evm.model.Contig20.736   evm.model.Contig28.23     1e-171
137-  4: evm.model.Contig20.739   evm.model.Contig28.25      2e-60
137-  5: evm.model.Contig20.741   evm.model.Contig28.27          0
137-  6: evm.model.Contig20.742   evm.model.Contig28.28      8e-75
137-  7: evm.model.Contig20.752   evm.model.Contig28.31     9e-174
137-  8: evm.model.Contig20.753   evm.model.Contig28.38          0
137-  9: evm.model.Contig20.755   evm.model.Contig28.49          0
## Alignment 138: score=427.0 e_value=4.5e-17 N=9 ew20&ew28 plus
138-  0: evm.model.Contig20.465   evm.model.Contig28.311     8e-17
138-  1: evm.model.Contig20.466   evm.model.Contig28.316         0
138-  2: evm.model.Contig20.467   evm.model.Contig28.317     2e-53
138-  3: evm.model.Contig20.479   evm.model.Contig28.323     1e-11
138-  4: evm.model.Contig20.482   evm.model.Contig28.327    2e-130
138-  5: evm.model.Contig20.484   evm.model.Contig28.329    1e-161
138-  6: evm.model.Contig20.487   evm.model.Contig28.330         0
138-  7: evm.model.Contig20.488   evm.model.Contig28.331    2e-134
138-  8: evm.model.Contig20.490   evm.model.Contig28.334    7e-124
## Alignment 139: score=291.0 e_value=1.4e-08 N=6 ew20&ew28 plus
139-  0: evm.model.Contig20.265   evm.model.Contig28.476         0
139-  1: evm.model.Contig20.268   evm.model.Contig28.477     1e-24
139-  2: evm.model.Contig20.269   evm.model.Contig28.478         0
139-  3: evm.model.Contig20.270   evm.model.Contig28.479         0
139-  4: evm.model.Contig20.271   evm.model.Contig28.481    2e-180
139-  5: evm.model.Contig20.274   evm.model.Contig28.488     4e-42
## Alignment 140: score=1222.0 e_value=1.6e-88 N=28 ew20&ew28 minus
140-  0: evm.model.Contig20.404   evm.model.Contig28.363         0
140-  1: evm.model.Contig20.407   evm.model.Contig28.356         0
140-  2: evm.model.Contig20.414   evm.model.Contig28.354    2e-102
140-  3: evm.model.Contig20.417   evm.model.Contig28.353     2e-12
140-  4: evm.model.Contig20.423   evm.model.Contig28.349         0
140-  5: evm.model.Contig20.426   evm.model.Contig28.344     4e-73
140-  6: evm.model.Contig20.429   evm.model.Contig28.342         0
140-  7: evm.model.Contig20.430   evm.model.Contig28.341     3e-26
140-  8: evm.model.Contig20.445   evm.model.Contig28.339     2e-57
140-  9: evm.model.Contig20.458   evm.model.Contig28.338     2e-60
140- 10: evm.model.Contig20.476   evm.model.Contig28.317     2e-56
140- 11: evm.model.Contig20.478   evm.model.Contig28.316         0
140- 12: evm.model.Contig20.479   evm.model.Contig28.311     1e-25
140- 13: evm.model.Contig20.499   evm.model.Contig28.301    2e-111
140- 14: evm.model.Contig20.502   evm.model.Contig28.300    4e-173
140- 15: evm.model.Contig20.505   evm.model.Contig28.295         0
140- 16: evm.model.Contig20.509   evm.model.Contig28.272     9e-56
140- 17: evm.model.Contig20.513   evm.model.Contig28.265         0
140- 18: evm.model.Contig20.516   evm.model.Contig28.258         0
140- 19: evm.model.Contig20.524   evm.model.Contig28.254     4e-35
140- 20: evm.model.Contig20.529   evm.model.Contig28.250     5e-49
140- 21: evm.model.Contig20.532   evm.model.Contig28.249    5e-103
140- 22: evm.model.Contig20.533   evm.model.Contig28.244     4e-76
140- 23: evm.model.Contig20.538   evm.model.Contig28.243     3e-93
140- 24: evm.model.Contig20.545   evm.model.Contig28.235    2e-114
140- 25: evm.model.Contig20.547   evm.model.Contig28.230     4e-50
140- 26: evm.model.Contig20.549   evm.model.Contig28.229    6e-158
140- 27: evm.model.Contig20.560   evm.model.Contig28.226    3e-149
## Alignment 141: score=1181.0 e_value=3.7e-79 N=26 ew20&ew28 minus
```

```
141-  0: evm.model.Contig20.561   evm.model.Contig28.123    8e-153
141-  1: evm.model.Contig20.563   evm.model.Contig28.121    3e-142
141-  2: evm.model.Contig20.576   evm.model.Contig28.119         0
141-  3: evm.model.Contig20.579   evm.model.Contig28.116     3e-91
141-  4: evm.model.Contig20.580   evm.model.Contig28.112     2e-54
141-  5: evm.model.Contig20.586   evm.model.Contig28.111    8e-131
141-  6: evm.model.Contig20.587   evm.model.Contig28.109         0
141-  7: evm.model.Contig20.588   evm.model.Contig28.108         0
141-  8: evm.model.Contig20.594   evm.model.Contig28.107         0
141-  9: evm.model.Contig20.595   evm.model.Contig28.106     7e-56
141- 10: evm.model.Contig20.602   evm.model.Contig28.101         0
141- 11: evm.model.Contig20.609   evm.model.Contig28.100         0
141- 12: evm.model.Contig20.614   evm.model.Contig28.97      3e-84
141- 13: evm.model.Contig20.639   evm.model.Contig28.71      1e-36
141- 14: evm.model.Contig20.644   evm.model.Contig28.59      3e-26
141- 15: evm.model.Contig20.654   evm.model.Contig28.55      3e-20
141- 16: evm.model.Contig20.655   evm.model.Contig28.53          0
141- 17: evm.model.Contig20.661   evm.model.Contig28.52          0
141- 18: evm.model.Contig20.664   evm.model.Contig28.51     3e-121
141- 19: evm.model.Contig20.669   evm.model.Contig28.49      2e-27
141- 20: evm.model.Contig20.671   evm.model.Contig28.47      2e-76
141- 21: evm.model.Contig20.678   evm.model.Contig28.43      8e-65
141- 22: evm.model.Contig20.679   evm.model.Contig28.42          0
141- 23: evm.model.Contig20.684   evm.model.Contig28.38          0
141- 24: evm.model.Contig20.687   evm.model.Contig28.36     5e-168
141- 25: evm.model.Contig20.688   evm.model.Contig28.31     3e-164
## Alignment 142: score=713.0 e_value=1.9e-43 N=16 ew20&ew28 minus
142-  0: evm.model.Contig20.375   evm.model.Contig28.191     3e-66
142-  1: evm.model.Contig20.381   evm.model.Contig28.183     2e-26
142-  2: evm.model.Contig20.386   evm.model.Contig28.181     5e-14
142-  3: evm.model.Contig20.387   evm.model.Contig28.179     8e-52
142-  4: evm.model.Contig20.391   evm.model.Contig28.177         0
142-  5: evm.model.Contig20.402   evm.model.Contig28.170     1e-46
142-  6: evm.model.Contig20.426   evm.model.Contig28.156     6e-40
142-  7: evm.model.Contig20.440   evm.model.Contig28.150         0
142-  8: evm.model.Contig20.445   evm.model.Contig28.148     2e-67
142-  9: evm.model.Contig20.453   evm.model.Contig28.147     7e-44
142- 10: evm.model.Contig20.459   evm.model.Contig28.143    4e-171
142- 11: evm.model.Contig20.462   evm.model.Contig28.140     6e-50
142- 12: evm.model.Contig20.464   evm.model.Contig28.139         0
142- 13: evm.model.Contig20.465   evm.model.Contig28.135     1e-17
142- 14: evm.model.Contig20.467   evm.model.Contig28.130     9e-32
142- 15: evm.model.Contig20.468   evm.model.Contig28.129    5e-153
## Alignment 143: score=641.0 e_value=7.4e-33 N=14 ew20&ew28 minus
143-  0: evm.model.Contig20.308   evm.model.Contig28.566     4e-85
143-  1: evm.model.Contig20.310   evm.model.Contig28.563     4e-46
143-  2: evm.model.Contig20.314   evm.model.Contig28.558     8e-97
143-  3: evm.model.Contig20.321   evm.model.Contig28.552         0
143-  4: evm.model.Contig20.331   evm.model.Contig28.546         0
143-  5: evm.model.Contig20.333   evm.model.Contig28.544         0
143-  6: evm.model.Contig20.337   evm.model.Contig28.541     1e-68
143-  7: evm.model.Contig20.340   evm.model.Contig28.538     2e-28
143-  8: evm.model.Contig20.344   evm.model.Contig28.533     1e-80
143-  9: evm.model.Contig20.345   evm.model.Contig28.531    4e-128
```

```
143- 10: evm.model.Contig20.352   evm.model.Contig28.525     2e-47
143- 11: evm.model.Contig20.357   evm.model.Contig28.523     8e-39
143- 12: evm.model.Contig20.363   evm.model.Contig28.505     2e-53
143- 13: evm.model.Contig20.364   evm.model.Contig28.504     2e-32
## Alignment 144: score=414.0 e_value=1.9e-17 N=9 ew20&ew28 minus
144-  0: evm.model.Contig20.441   evm.model.Contig28.162     4e-20
144-  1: evm.model.Contig20.443   evm.model.Contig28.161         0
144-  2: evm.model.Contig20.445   evm.model.Contig28.158     9e-68
144-  3: evm.model.Contig20.448   evm.model.Contig28.155     8e-34
144-  4: evm.model.Contig20.449   evm.model.Contig28.152    2e-122
144-  5: evm.model.Contig20.455   evm.model.Contig28.149     1e-83
144-  6: evm.model.Contig20.461   evm.model.Contig28.138    2e-170
144-  7: evm.model.Contig20.471   evm.model.Contig28.133         0
144-  8: evm.model.Contig20.477   evm.model.Contig28.130     3e-33
## Alignment 145: score=361.0 e_value=2.1e-15 N=8 ew20&ew28 minus
145-  0: evm.model.Contig20.271   evm.model.Contig28.492     2e-98
145-  1: evm.model.Contig20.272   evm.model.Contig28.491     2e-84
145-  2: evm.model.Contig20.277   evm.model.Contig28.479         0
145-  3: evm.model.Contig20.278   evm.model.Contig28.478    3e-112
145-  4: evm.model.Contig20.285   evm.model.Contig28.460         0
145-  5: evm.model.Contig20.289   evm.model.Contig28.453     5e-42
145-  6: evm.model.Contig20.290   evm.model.Contig28.452    2e-174
145-  7: evm.model.Contig20.296   evm.model.Contig28.446         0
## Alignment 146: score=1138.0 e_value=1.9e-74 N=24 ew21&ew36 plus
146-  0: evm.model.Contig21.7     evm.model.Contig36.442     1e-28
146-  1: evm.model.Contig21.10    evm.model.Contig36.450         0
146-  2: evm.model.Contig21.13    evm.model.Contig36.456     3e-18
146-  3: evm.model.Contig21.17    evm.model.Contig36.465     3e-66
146-  4: evm.model.Contig21.19    evm.model.Contig36.467     2e-66
146-  5: evm.model.Contig21.20    evm.model.Contig36.471    9e-104
146-  6: evm.model.Contig21.21    evm.model.Contig36.474         0
146-  7: evm.model.Contig21.22    evm.model.Contig36.475         0
146-  8: evm.model.Contig21.23    evm.model.Contig36.476         0
146-  9: evm.model.Contig21.24    evm.model.Contig36.477    2e-171
146- 10: evm.model.Contig21.29    evm.model.Contig36.479         0
146- 11: evm.model.Contig21.30    evm.model.Contig36.480     6e-57
146- 12: evm.model.Contig21.34    evm.model.Contig36.486     4e-31
146- 13: evm.model.Contig21.35    evm.model.Contig36.490     3e-66
146- 14: evm.model.Contig21.46    evm.model.Contig36.491     3e-73
146- 15: evm.model.Contig21.49    evm.model.Contig36.494         0
146- 16: evm.model.Contig21.50    evm.model.Contig36.495    7e-147
146- 17: evm.model.Contig21.56    evm.model.Contig36.498     7e-87
146- 18: evm.model.Contig21.57    evm.model.Contig36.499     2e-15
146- 19: evm.model.Contig21.60    evm.model.Contig36.501     5e-44
146- 20: evm.model.Contig21.61    evm.model.Contig36.502     1e-75
146- 21: evm.model.Contig21.66    evm.model.Contig36.504     5e-88
146- 22: evm.model.Contig21.68    evm.model.Contig36.505         0
146- 23: evm.model.Contig21.69    evm.model.Contig36.506    2e-169
## Alignment 147: score=302.0 e_value=2.7e-13 N=7 ew21&ew36 plus
147-  0: evm.model.Contig21.220   evm.model.Contig36.318     8e-19
147-  1: evm.model.Contig21.226   evm.model.Contig36.320         0
147-  2: evm.model.Contig21.237   evm.model.Contig36.323     1e-66
147-  3: evm.model.Contig21.238   evm.model.Contig36.327         0
147-  4: evm.model.Contig21.239   evm.model.Contig36.353     3e-60
```

```
147-  5: evm.model.Contig21.240   evm.model.Contig36.355      3e-74
147-  6: evm.model.Contig21.245   evm.model.Contig36.357      2e-93
## Alignment 148: score=281.0 e_value=7e-09 N=6 ew21&ew36 plus
148-  0: evm.model.Contig21.239   evm.model.Contig36.27       2e-78
148-  1: evm.model.Contig21.240   evm.model.Contig36.28       5e-72
148-  2: evm.model.Contig21.245   evm.model.Contig36.30       2e-95
148-  3: evm.model.Contig21.249   evm.model.Contig36.37           0
148-  4: evm.model.Contig21.258   evm.model.Contig36.41      3e-131
148-  5: evm.model.Contig21.260   evm.model.Contig36.42      9e-175
## Alignment 149: score=552.0 e_value=2.1e-27 N=12 ew21&ew36 minus
149-  0: evm.model.Contig21.299   evm.model.Contig36.307          0
149-  1: evm.model.Contig21.307   evm.model.Contig36.300          0
149-  2: evm.model.Contig21.308   evm.model.Contig36.299          0
149-  3: evm.model.Contig21.311   evm.model.Contig36.297      2e-30
149-  4: evm.model.Contig21.323   evm.model.Contig36.292     8e-117
149-  5: evm.model.Contig21.328   evm.model.Contig36.291      2e-49
149-  6: evm.model.Contig21.332   evm.model.Contig36.280      2e-99
149-  7: evm.model.Contig21.338   evm.model.Contig36.278      3e-95
149-  8: evm.model.Contig21.342   evm.model.Contig36.274     4e-167
149-  9: evm.model.Contig21.343   evm.model.Contig36.273     1e-152
149- 10: evm.model.Contig21.345   evm.model.Contig36.270     7e-121
149- 11: evm.model.Contig21.350   evm.model.Contig36.265          0
## Alignment 150: score=502.0 e_value=5.2e-27 N=12 ew21&ew36 minus
150-  0: evm.model.Contig21.487   evm.model.Contig36.144          0
150-  1: evm.model.Contig21.501   evm.model.Contig36.130      4e-60
150-  2: evm.model.Contig21.510   evm.model.Contig36.110      1e-15
150-  3: evm.model.Contig21.511   evm.model.Contig36.100     2e-159
150-  4: evm.model.Contig21.523   evm.model.Contig36.94      8e-108
150-  5: evm.model.Contig21.526   evm.model.Contig36.79      3e-165
150-  6: evm.model.Contig21.533   evm.model.Contig36.75      2e-160
150-  7: evm.model.Contig21.540   evm.model.Contig36.69       1e-93
150-  8: evm.model.Contig21.543   evm.model.Contig36.67      5e-129
150-  9: evm.model.Contig21.545   evm.model.Contig36.65           0
150- 10: evm.model.Contig21.550   evm.model.Contig36.59       6e-89
150- 11: evm.model.Contig21.559   evm.model.Contig36.46           0
## Alignment 151: score=259.0 e_value=9.5e-12 N=6 ew21&ew36 minus
151-  0: evm.model.Contig21.201   evm.model.Contig36.378     5e-119
151-  1: evm.model.Contig21.203   evm.model.Contig36.371      3e-15
151-  2: evm.model.Contig21.205   evm.model.Contig36.370      1e-99
151-  3: evm.model.Contig21.206   evm.model.Contig36.366      2e-56
151-  4: evm.model.Contig21.214   evm.model.Contig36.365      1e-51
151-  5: evm.model.Contig21.239   evm.model.Contig36.353      3e-60
## Alignment 152: score=294.0 e_value=2.1e-09 N=6 ew22&ew22 plus
152-  0: evm.model.Contig22.540   evm.model.Contig22.575          0
152-  1: evm.model.Contig22.541   evm.model.Contig22.576          0
152-  2: evm.model.Contig22.542   evm.model.Contig22.577          0
152-  3: evm.model.Contig22.543   evm.model.Contig22.579          0
152-  4: evm.model.Contig22.547   evm.model.Contig22.585          0
152-  5: evm.model.Contig22.548   evm.model.Contig22.586          0
## Alignment 153: score=411.0 e_value=6.8e-19 N=9 ew22&ew22 minus
153-  0: evm.model.Contig22.816   evm.model.Contig22.892     2e-122
153-  1: evm.model.Contig22.817   evm.model.Contig22.891      3e-44
153-  2: evm.model.Contig22.824   evm.model.Contig22.890      3e-15
153-  3: evm.model.Contig22.848   evm.model.Contig22.887      8e-83
```

```
153-  4: evm.model.Contig22.850   evm.model.Contig22.882     9e-46
153-  5: evm.model.Contig22.851   evm.model.Contig22.881     5e-55
153-  6: evm.model.Contig22.853   evm.model.Contig22.880     2e-26
153-  7: evm.model.Contig22.857   evm.model.Contig22.875         0
153-  8: evm.model.Contig22.859   evm.model.Contig22.874    3e-122
## Alignment 154: score=367.0 e_value=6.6e-15 N=8 ew22&ew22 minus
154-  0: evm.model.Contig22.119   evm.model.Contig22.176    6e-163
154-  1: evm.model.Contig22.120   evm.model.Contig22.174    2e-117
154-  2: evm.model.Contig22.121   evm.model.Contig22.169     5e-58
154-  3: evm.model.Contig22.122   evm.model.Contig22.168    1e-106
154-  4: evm.model.Contig22.123   evm.model.Contig22.153         0
154-  5: evm.model.Contig22.124   evm.model.Contig22.150         0
154-  6: evm.model.Contig22.125   evm.model.Contig22.148         0
154-  7: evm.model.Contig22.129   evm.model.Contig22.136         0
## Alignment 155: score=346.0 e_value=1.4e-11 N=7 ew22&ew22 minus
155-  0: evm.model.Contig22.708   evm.model.Contig22.727         0
155-  1: evm.model.Contig22.709   evm.model.Contig22.726    6e-171
155-  2: evm.model.Contig22.711   evm.model.Contig22.725     6e-44
155-  3: evm.model.Contig22.712   evm.model.Contig22.723     2e-50
155-  4: evm.model.Contig22.713   evm.model.Contig22.722    2e-140
155-  5: evm.model.Contig22.715   evm.model.Contig22.720    2e-105
155-  6: evm.model.Contig22.716   evm.model.Contig22.718    1e-118
## Alignment 156: score=298.0 e_value=8.3e-08 N=6 ew22&ew22 minus
156-  0: evm.model.Contig22.690   evm.model.Contig22.705    4e-128
156-  1: evm.model.Contig22.691   evm.model.Contig22.704         0
156-  2: evm.model.Contig22.692   evm.model.Contig22.703         0
156-  3: evm.model.Contig22.693   evm.model.Contig22.702         0
156-  4: evm.model.Contig22.695   evm.model.Contig22.699         0
156-  5: evm.model.Contig22.696   evm.model.Contig22.698         0
## Alignment 157: score=296.0 e_value=4.4e-09 N=6 ew22&ew22 minus
157-  0: evm.model.Contig22.762   evm.model.Contig22.788    1e-140
157-  1: evm.model.Contig22.763   evm.model.Contig22.787    6e-156
157-  2: evm.model.Contig22.764   evm.model.Contig22.782     1e-94
157-  3: evm.model.Contig22.765   evm.model.Contig22.781         0
157-  4: evm.model.Contig22.766   evm.model.Contig22.780         0
157-  5: evm.model.Contig22.767   evm.model.Contig22.779    7e-108
## Alignment 158: score=283.0 e_value=1.8e-12 N=6 ew22&ew22 minus
158-  0: evm.model.Contig22.22    evm.model.Contig22.65          0
158-  1: evm.model.Contig22.23    evm.model.Contig22.64          0
158-  2: evm.model.Contig22.24    evm.model.Contig22.63          0
158-  3: evm.model.Contig22.25    evm.model.Contig22.62          0
158-  4: evm.model.Contig22.41    evm.model.Contig22.45      4e-65
158-  5: evm.model.Contig22.42    evm.model.Contig22.43          0
## Alignment 159: score=250.0 e_value=9.1e-06 N=5 ew22&ew22 minus
159-  0: evm.model.Contig22.388   evm.model.Contig22.407         0
159-  1: evm.model.Contig22.389   evm.model.Contig22.406     8e-69
159-  2: evm.model.Contig22.390   evm.model.Contig22.405     4e-92
159-  3: evm.model.Contig22.391   evm.model.Contig22.404         0
159-  4: evm.model.Contig22.392   evm.model.Contig22.403     1e-50
## Alignment 160: score=347.0 e_value=6.8e-11 N=7 ew23&ew23 plus
160-  0: evm.model.Contig23.1     evm.model.Contig23.168    1e-129
160-  1: evm.model.Contig23.2     evm.model.Contig23.169         0
160-  2: evm.model.Contig23.3     evm.model.Contig23.172     5e-42
160-  3: evm.model.Contig23.4     evm.model.Contig23.174         0
```

```
160-  4: evm.model.Contig23.5     evm.model.Contig23.175        0
160-  5: evm.model.Contig23.6     evm.model.Contig23.176        0
160-  6: evm.model.Contig23.7     evm.model.Contig23.177        0
## Alignment 161: score=348.0 e_value=1.7e-10 N=7 ew23&ew23 minus
161-  0: evm.model.Contig23.301   evm.model.Contig23.319    9e-65
161-  1: evm.model.Contig23.302   evm.model.Contig23.318        0
161-  2: evm.model.Contig23.303   evm.model.Contig23.317    1e-48
161-  3: evm.model.Contig23.304   evm.model.Contig23.316        0
161-  4: evm.model.Contig23.305   evm.model.Contig23.315   1e-180
161-  5: evm.model.Contig23.306   evm.model.Contig23.314        0
161-  6: evm.model.Contig23.309   evm.model.Contig23.312    9e-50
## Alignment 162: score=287.0 e_value=1.9e-12 N=6 ew23&ew23 minus
162-  0: evm.model.Contig23.492   evm.model.Contig23.527        0
162-  1: evm.model.Contig23.501   evm.model.Contig23.513    3e-22
162-  2: evm.model.Contig23.502   evm.model.Contig23.512    1e-69
162-  3: evm.model.Contig23.503   evm.model.Contig23.511        0
162-  4: evm.model.Contig23.504   evm.model.Contig23.510        0
162-  5: evm.model.Contig23.505   evm.model.Contig23.509        0
## Alignment 163: score=418.0 e_value=3.2e-19 N=9 ew24&ew24 minus
163-  0: evm.model.Contig24.326   evm.model.Contig24.569   6e-141
163-  1: evm.model.Contig24.327   evm.model.Contig24.568   5e-148
163-  2: evm.model.Contig24.328   evm.model.Contig24.564        0
163-  3: evm.model.Contig24.331   evm.model.Contig24.563    2e-90
163-  4: evm.model.Contig24.332   evm.model.Contig24.562   3e-127
163-  5: evm.model.Contig24.336   evm.model.Contig24.560   6e-156
163-  6: evm.model.Contig24.338   evm.model.Contig24.559        0
163-  7: evm.model.Contig24.339   evm.model.Contig24.558    5e-90
163-  8: evm.model.Contig24.363   evm.model.Contig24.545        0
## Alignment 164: score=270.0 e_value=9.1e-10 N=6 ew24&ew24 minus
164-  0: evm.model.Contig24.371   evm.model.Contig24.503   1e-125
164-  1: evm.model.Contig24.372   evm.model.Contig24.499    4e-22
164-  2: evm.model.Contig24.388   evm.model.Contig24.497        0
164-  3: evm.model.Contig24.391   evm.model.Contig24.496        0
164-  4: evm.model.Contig24.401   evm.model.Contig24.494    2e-26
164-  5: evm.model.Contig24.403   evm.model.Contig24.492    5e-76
## Alignment 165: score=379.0 e_value=1.2e-17 N=9 ew24&ew33 plus
165-  0: evm.model.Contig24.31    evm.model.Contig33.409   2e-154
165-  1: evm.model.Contig24.32    evm.model.Contig33.410    2e-64
165-  2: evm.model.Contig24.45    evm.model.Contig33.429        0
165-  3: evm.model.Contig24.48    evm.model.Contig33.430        0
165-  4: evm.model.Contig24.52    evm.model.Contig33.431   4e-114
165-  5: evm.model.Contig24.56    evm.model.Contig33.448    6e-30
165-  6: evm.model.Contig24.60    evm.model.Contig33.468   3e-113
165-  7: evm.model.Contig24.66    evm.model.Contig33.471    1e-82
165-  8: evm.model.Contig24.68    evm.model.Contig33.480    2e-79
## Alignment 166: score=304.0 e_value=6.9e-13 N=7 ew24&ew33 plus
166-  0: evm.model.Contig24.242   evm.model.Contig33.214    5e-18
166-  1: evm.model.Contig24.247   evm.model.Contig33.216    3e-68
166-  2: evm.model.Contig24.248   evm.model.Contig33.217        0
166-  3: evm.model.Contig24.259   evm.model.Contig33.227    4e-51
166-  4: evm.model.Contig24.266   evm.model.Contig33.230    3e-36
166-  5: evm.model.Contig24.285   evm.model.Contig33.246        0
166-  6: evm.model.Contig24.294   evm.model.Contig33.254    4e-47
## Alignment 167: score=272.0 e_value=4.1e-09 N=6 ew24&ew33 plus
```

```
167-  0: evm.model.Contig24.188   evm.model.Contig33.339        0
167-  1: evm.model.Contig24.190   evm.model.Contig33.348    5e-43
167-  2: evm.model.Contig24.192   evm.model.Contig33.353        0
167-  3: evm.model.Contig24.197   evm.model.Contig33.356    4e-90
167-  4: evm.model.Contig24.204   evm.model.Contig33.366        0
167-  5: evm.model.Contig24.208   evm.model.Contig33.368        0
## Alignment 168: score=582.0 e_value=8.4e-32 N=13 ew24&ew33 minus
168-  0: evm.model.Contig24.135   evm.model.Contig33.334        0
168-  1: evm.model.Contig24.137   evm.model.Contig33.333   2e-131
168-  2: evm.model.Contig24.138   evm.model.Contig33.331        0
168-  3: evm.model.Contig24.142   evm.model.Contig33.327    8e-20
168-  4: evm.model.Contig24.145   evm.model.Contig33.325        0
168-  5: evm.model.Contig24.151   evm.model.Contig33.315    6e-89
168-  6: evm.model.Contig24.152   evm.model.Contig33.304    4e-66
168-  7: evm.model.Contig24.153   evm.model.Contig33.303   9e-122
168-  8: evm.model.Contig24.154   evm.model.Contig33.299    4e-41
168-  9: evm.model.Contig24.158   evm.model.Contig33.296        0
168- 10: evm.model.Contig24.165   evm.model.Contig33.292        0
168- 11: evm.model.Contig24.169   evm.model.Contig33.284   1e-150
168- 12: evm.model.Contig24.193   evm.model.Contig33.280    1e-35
## Alignment 169: score=487.0 e_value=1.5e-28 N=12 ew24&ew33 minus
169-  0: evm.model.Contig24.374   evm.model.Contig33.160    3e-32
169-  1: evm.model.Contig24.390   evm.model.Contig33.135    9e-42
169-  2: evm.model.Contig24.392   evm.model.Contig33.134        0
169-  3: evm.model.Contig24.413   evm.model.Contig33.110    2e-18
169-  4: evm.model.Contig24.418   evm.model.Contig33.107    2e-50
169-  5: evm.model.Contig24.427   evm.model.Contig33.98    1e-105
169-  6: evm.model.Contig24.431   evm.model.Contig33.95     6e-96
169-  7: evm.model.Contig24.452   evm.model.Contig33.89     5e-75
169-  8: evm.model.Contig24.461   evm.model.Contig33.77     8e-51
169-  9: evm.model.Contig24.463   evm.model.Contig33.73     2e-19
169- 10: evm.model.Contig24.474   evm.model.Contig33.63         0
169- 11: evm.model.Contig24.481   evm.model.Contig33.57         0
## Alignment 170: score=408.0 e_value=1.4e-16 N=9 ew24&ew33 minus
170-  0: evm.model.Contig24.91    evm.model.Contig33.400    2e-50
170-  1: evm.model.Contig24.92    evm.model.Contig33.395        0
170-  2: evm.model.Contig24.98    evm.model.Contig33.393        0
170-  3: evm.model.Contig24.103   evm.model.Contig33.388    2e-24
170-  4: evm.model.Contig24.109   evm.model.Contig33.384    1e-78
170-  5: evm.model.Contig24.112   evm.model.Contig33.376    1e-31
170-  6: evm.model.Contig24.121   evm.model.Contig33.368        0
170-  7: evm.model.Contig24.128   evm.model.Contig33.367   2e-131
170-  8: evm.model.Contig24.132   evm.model.Contig33.365        0
## Alignment 171: score=321.0 e_value=1.5e-11 N=7 ew24&ew33 minus
171-  0: evm.model.Contig24.37    evm.model.Contig33.438    1e-34
171-  1: evm.model.Contig24.41    evm.model.Contig33.436        0
171-  2: evm.model.Contig24.43    evm.model.Contig33.434    6e-93
171-  3: evm.model.Contig24.49    evm.model.Contig33.428        0
171-  4: evm.model.Contig24.50    evm.model.Contig33.423        0
171-  5: evm.model.Contig24.53    evm.model.Contig33.418    6e-96
171-  6: evm.model.Contig24.54    evm.model.Contig33.405    6e-26
## Alignment 172: score=280.0 e_value=1.7e-12 N=7 ew24&ew33 minus
172-  0: evm.model.Contig24.243   evm.model.Contig33.210        0
172-  1: evm.model.Contig24.264   evm.model.Contig33.192    2e-37
```

```
172-  2: evm.model.Contig24.277   evm.model.Contig33.186     2e-94
172-  3: evm.model.Contig24.285   evm.model.Contig33.181         0
172-  4: evm.model.Contig24.286   evm.model.Contig33.180    3e-106
172-  5: evm.model.Contig24.292   evm.model.Contig33.171         0
172-  6: evm.model.Contig24.316   evm.model.Contig33.156         0
## Alignment 173: score=261.0 e_value=1.4e-08 N=6 ew24&ew33 minus
173-  0: evm.model.Contig24.515   evm.model.Contig33.289     2e-82
173-  1: evm.model.Contig24.528   evm.model.Contig33.275         0
173-  2: evm.model.Contig24.529   evm.model.Contig33.274         0
173-  3: evm.model.Contig24.538   evm.model.Contig33.270    1e-133
173-  4: evm.model.Contig24.544   evm.model.Contig33.260         0
173-  5: evm.model.Contig24.552   evm.model.Contig33.250     4e-30
## Alignment 174: score=343.0 e_value=2.8e-12 N=7 ew25&ew25 minus
174-  0: evm.model.Contig25.202   evm.model.Contig25.225    2e-159
174-  1: evm.model.Contig25.203   evm.model.Contig25.223         0
174-  2: evm.model.Contig25.204   evm.model.Contig25.222         0
174-  3: evm.model.Contig25.207   evm.model.Contig25.221         0
174-  4: evm.model.Contig25.209   evm.model.Contig25.220         0
174-  5: evm.model.Contig25.210   evm.model.Contig25.219         0
174-  6: evm.model.Contig25.212   evm.model.Contig25.215     2e-95
## Alignment 175: score=265.0 e_value=2.3e-11 N=6 ew25&ew25 minus
175-  0: evm.model.Contig25.240   evm.model.Contig25.299         0
175-  1: evm.model.Contig25.241   evm.model.Contig25.275     1e-84
175-  2: evm.model.Contig25.242   evm.model.Contig25.274         0
175-  3: evm.model.Contig25.243   evm.model.Contig25.273     7e-56
175-  4: evm.model.Contig25.249   evm.model.Contig25.260     3e-28
175-  5: evm.model.Contig25.250   evm.model.Contig25.259         0
## Alignment 176: score=290.0 e_value=7.8e-12 N=6 ew26&ew26 minus
176-  0: evm.model.Contig26.369   evm.model.Contig26.395         0
176-  1: evm.model.Contig26.370   evm.model.Contig26.393     6e-48
176-  2: evm.model.Contig26.371   evm.model.Contig26.392         0
176-  3: evm.model.Contig26.372   evm.model.Contig26.391         0
176-  4: evm.model.Contig26.373   evm.model.Contig26.390         0
176-  5: evm.model.Contig26.375   evm.model.Contig26.380    2e-144
## Alignment 177: score=571.0 e_value=1.6e-33 N=12 ew27&ew27 plus
177-  0: evm.model.Contig27.252   evm.model.Contig27.589     5e-71
177-  1: evm.model.Contig27.259   evm.model.Contig27.597         0
177-  2: evm.model.Contig27.260   evm.model.Contig27.598    4e-123
177-  3: evm.model.Contig27.261   evm.model.Contig27.599    6e-112
177-  4: evm.model.Contig27.262   evm.model.Contig27.600    3e-175
177-  5: evm.model.Contig27.263   evm.model.Contig27.601         0
177-  6: evm.model.Contig27.264   evm.model.Contig27.602     5e-80
177-  7: evm.model.Contig27.265   evm.model.Contig27.603         0
177-  8: evm.model.Contig27.266   evm.model.Contig27.604         0
177-  9: evm.model.Contig27.286   evm.model.Contig27.606    3e-135
177- 10: evm.model.Contig27.287   evm.model.Contig27.607         0
177- 11: evm.model.Contig27.291   evm.model.Contig27.611         0
## Alignment 178: score=382.0 e_value=1e-14 N=8 ew27&ew27 plus
178-  0: evm.model.Contig27.2     evm.model.Contig27.67      4e-87
178-  1: evm.model.Contig27.4     evm.model.Contig27.74          0
178-  2: evm.model.Contig27.5     evm.model.Contig27.75          0
178-  3: evm.model.Contig27.6     evm.model.Contig27.76     3e-119
178-  4: evm.model.Contig27.8     evm.model.Contig27.88     3e-168
178-  5: evm.model.Contig27.9     evm.model.Contig27.89          0
```

```
178-  6: evm.model.Contig27.10    evm.model.Contig27.90      1e-47
178-  7: evm.model.Contig27.11    evm.model.Contig27.92     1e-162
## Alignment 179: score=586.0 e_value=2.5e-28 N=12 ew27&ew27 minus
179-  0: evm.model.Contig27.612   evm.model.Contig27.654     1e-53
179-  1: evm.model.Contig27.613   evm.model.Contig27.653    7e-113
179-  2: evm.model.Contig27.616   evm.model.Contig27.651         0
179-  3: evm.model.Contig27.617   evm.model.Contig27.650         0
179-  4: evm.model.Contig27.618   evm.model.Contig27.644     6e-45
179-  5: evm.model.Contig27.621   evm.model.Contig27.643    2e-150
179-  6: evm.model.Contig27.622   evm.model.Contig27.639    6e-155
179-  7: evm.model.Contig27.623   evm.model.Contig27.638     1e-63
179-  8: evm.model.Contig27.624   evm.model.Contig27.637     5e-50
179-  9: evm.model.Contig27.626   evm.model.Contig27.634         0
179- 10: evm.model.Contig27.627   evm.model.Contig27.633         0
179- 11: evm.model.Contig27.628   evm.model.Contig27.632         0
## Alignment 180: score=299.0 e_value=3.7e-08 N=6 ew27&ew27 minus
180-  0: evm.model.Contig27.245   evm.model.Contig27.596     1e-35
180-  1: evm.model.Contig27.246   evm.model.Contig27.595         0
180-  2: evm.model.Contig27.247   evm.model.Contig27.594         0
180-  3: evm.model.Contig27.248   evm.model.Contig27.593     2e-66
180-  4: evm.model.Contig27.249   evm.model.Contig27.592         0
180-  5: evm.model.Contig27.251   evm.model.Contig27.591         0
## Alignment 181: score=296.0 e_value=4.5e-08 N=6 ew28&ew28 plus
181-  0: evm.model.Contig28.516   evm.model.Contig28.567     7e-38
181-  1: evm.model.Contig28.517   evm.model.Contig28.568         0
181-  2: evm.model.Contig28.518   evm.model.Contig28.570         0
181-  3: evm.model.Contig28.521   evm.model.Contig28.571     7e-97
181-  4: evm.model.Contig28.523   evm.model.Contig28.572         0
181-  5: evm.model.Contig28.524   evm.model.Contig28.573         0
## Alignment 182: score=291.0 e_value=2.6e-08 N=6 ew28&ew28 plus
182-  0: evm.model.Contig28.37    evm.model.Contig28.647         0
182-  1: evm.model.Contig28.39    evm.model.Contig28.648         0
182-  2: evm.model.Contig28.47    evm.model.Contig28.649         0
182-  3: evm.model.Contig28.48    evm.model.Contig28.650     4e-59
182-  4: evm.model.Contig28.49    evm.model.Contig28.651         0
182-  5: evm.model.Contig28.51    evm.model.Contig28.652     5e-66
## Alignment 183: score=320.0 e_value=4.2e-15 N=7 ew28&ew28 minus
183-  0: evm.model.Contig28.376   evm.model.Contig28.449     8e-27
183-  1: evm.model.Contig28.381   evm.model.Contig28.440    2e-169
183-  2: evm.model.Contig28.382   evm.model.Contig28.439         0
183-  3: evm.model.Contig28.403   evm.model.Contig28.419         0
183-  4: evm.model.Contig28.404   evm.model.Contig28.418    4e-147
183-  5: evm.model.Contig28.405   evm.model.Contig28.415    2e-134
183-  6: evm.model.Contig28.406   evm.model.Contig28.414         0
## Alignment 184: score=332.0 e_value=4.2e-12 N=7 ew29&ew29 plus
184-  0: evm.model.Contig29.221   evm.model.Contig29.522     9e-70
184-  1: evm.model.Contig29.224   evm.model.Contig29.527     2e-81
184-  2: evm.model.Contig29.225   evm.model.Contig29.528    1e-107
184-  3: evm.model.Contig29.232   evm.model.Contig29.529         0
184-  4: evm.model.Contig29.235   evm.model.Contig29.532     7e-34
184-  5: evm.model.Contig29.241   evm.model.Contig29.533         0
184-  6: evm.model.Contig29.243   evm.model.Contig29.534     9e-96
## Alignment 185: score=1363.0 e_value=1.7e-92 N=28 ew3&ew3 minus
185-  0: evm.model.Contig3.1180   evm.model.Contig3.1288    2e-129
```

```
185-  1: evm.model.Contig3.1181   evm.model.Contig3.1287         0
185-  2: evm.model.Contig3.1182   evm.model.Contig3.1286         0
185-  3: evm.model.Contig3.1183   evm.model.Contig3.1283         0
185-  4: evm.model.Contig3.1184   evm.model.Contig3.1282         0
185-  5: evm.model.Contig3.1185   evm.model.Contig3.1281    5e-144
185-  6: evm.model.Contig3.1186   evm.model.Contig3.1279         0
185-  7: evm.model.Contig3.1187   evm.model.Contig3.1277         0
185-  8: evm.model.Contig3.1188   evm.model.Contig3.1276         0
185-  9: evm.model.Contig3.1191   evm.model.Contig3.1275    1e-168
185- 10: evm.model.Contig3.1192   evm.model.Contig3.1273         0
185- 11: evm.model.Contig3.1193   evm.model.Contig3.1272         0
185- 12: evm.model.Contig3.1196   evm.model.Contig3.1271         0
185- 13: evm.model.Contig3.1197   evm.model.Contig3.1270         0
185- 14: evm.model.Contig3.1198   evm.model.Contig3.1267         0
185- 15: evm.model.Contig3.1199   evm.model.Contig3.1266    3e-139
185- 16: evm.model.Contig3.1200   evm.model.Contig3.1264         0
185- 17: evm.model.Contig3.1202   evm.model.Contig3.1263         0
185- 18: evm.model.Contig3.1203   evm.model.Contig3.1262    1e-151
185- 19: evm.model.Contig3.1204   evm.model.Contig3.1261         0
185- 20: evm.model.Contig3.1206   evm.model.Contig3.1260         0
185- 21: evm.model.Contig3.1207   evm.model.Contig3.1258         0
185- 22: evm.model.Contig3.1208   evm.model.Contig3.1253     1e-75
185- 23: evm.model.Contig3.1209   evm.model.Contig3.1251    1e-100
185- 24: evm.model.Contig3.1210   evm.model.Contig3.1250    9e-144
185- 25: evm.model.Contig3.1211   evm.model.Contig3.1249         0
185- 26: evm.model.Contig3.1212   evm.model.Contig3.1248         0
185- 27: evm.model.Contig3.1226   evm.model.Contig3.1230         0
## Alignment 186: score=493.0 e_value=5.6e-20 N=10 ew3&ew3 minus
186-  0: evm.model.Contig3.744    evm.model.Contig3.778     2e-155
186-  1: evm.model.Contig3.746    evm.model.Contig3.777      6e-63
186-  2: evm.model.Contig3.748    evm.model.Contig3.776     3e-121
186-  3: evm.model.Contig3.749    evm.model.Contig3.775      5e-48
186-  4: evm.model.Contig3.750    evm.model.Contig3.772          0
186-  5: evm.model.Contig3.751    evm.model.Contig3.771      2e-79
186-  6: evm.model.Contig3.752    evm.model.Contig3.770          0
186-  7: evm.model.Contig3.753    evm.model.Contig3.769      4e-85
186-  8: evm.model.Contig3.754    evm.model.Contig3.765     1e-143
186-  9: evm.model.Contig3.755    evm.model.Contig3.764          0
## Alignment 187: score=300.0 e_value=9.7e-10 N=6 ew3&ew3 minus
187-  0: evm.model.Contig3.564    evm.model.Contig3.583          0
187-  1: evm.model.Contig3.565    evm.model.Contig3.582     9e-151
187-  2: evm.model.Contig3.566    evm.model.Contig3.581      6e-32
187-  3: evm.model.Contig3.567    evm.model.Contig3.580          0
187-  4: evm.model.Contig3.568    evm.model.Contig3.579          0
187-  5: evm.model.Contig3.569    evm.model.Contig3.578     5e-133
## Alignment 188: score=297.0 e_value=7.1e-08 N=6 ew3&ew3 minus
188-  0: evm.model.Contig3.282    evm.model.Contig3.658     6e-141
188-  1: evm.model.Contig3.283    evm.model.Contig3.655          0
188-  2: evm.model.Contig3.284    evm.model.Contig3.653      1e-74
188-  3: evm.model.Contig3.285    evm.model.Contig3.652      3e-96
188-  4: evm.model.Contig3.286    evm.model.Contig3.651          0
188-  5: evm.model.Contig3.287    evm.model.Contig3.650          0
## Alignment 189: score=523.0 e_value=4.1e-31 N=13 ew3&ew32 plus
189-  0: evm.model.Contig3.14     evm.model.Contig32.296         0
```

```
189-  1: evm.model.Contig3.31     evm.model.Contig32.306    1e-113
189-  2: evm.model.Contig3.34     evm.model.Contig32.313     3e-21
189-  3: evm.model.Contig3.38     evm.model.Contig32.333    2e-177
189-  4: evm.model.Contig3.51     evm.model.Contig32.354         0
189-  5: evm.model.Contig3.52     evm.model.Contig32.355     7e-11
189-  6: evm.model.Contig3.54     evm.model.Contig32.360     6e-37
189-  7: evm.model.Contig3.68     evm.model.Contig32.372     1e-29
189-  8: evm.model.Contig3.84     evm.model.Contig32.381     1e-18
189-  9: evm.model.Contig3.88     evm.model.Contig32.389    4e-111
189- 10: evm.model.Contig3.90     evm.model.Contig32.390         0
189- 11: evm.model.Contig3.104    evm.model.Contig32.415         0
189- 12: evm.model.Contig3.107    evm.model.Contig32.417     1e-81
## Alignment 190: score=463.0 e_value=4e-27 N=11 ew3&ew32 plus
190-  0: evm.model.Contig3.210    evm.model.Contig32.74      2e-17
190-  1: evm.model.Contig3.236    evm.model.Contig32.82      2e-71
190-  2: evm.model.Contig3.243    evm.model.Contig32.106         0
190-  3: evm.model.Contig3.253    evm.model.Contig32.118         0
190-  4: evm.model.Contig3.258    evm.model.Contig32.129         0
190-  5: evm.model.Contig3.259    evm.model.Contig32.130     1e-60
190-  6: evm.model.Contig3.262    evm.model.Contig32.134    1e-110
190-  7: evm.model.Contig3.264    evm.model.Contig32.135         0
190-  8: evm.model.Contig3.267    evm.model.Contig32.137     5e-63
190-  9: evm.model.Contig3.272    evm.model.Contig32.149         0
190- 10: evm.model.Contig3.274    evm.model.Contig32.150     9e-44
## Alignment 191: score=322.0 e_value=4.5e-14 N=8 ew3&ew32 minus
191-  0: evm.model.Contig3.444    evm.model.Contig32.221         0
191-  1: evm.model.Contig3.457    evm.model.Contig32.219     8e-19
191-  2: evm.model.Contig3.461    evm.model.Contig32.200    1e-170
191-  3: evm.model.Contig3.471    evm.model.Contig32.194     2e-83
191-  4: evm.model.Contig3.475    evm.model.Contig32.192     6e-12
191-  5: evm.model.Contig3.493    evm.model.Contig32.177     2e-15
191-  6: evm.model.Contig3.502    evm.model.Contig32.163     6e-61
191-  7: evm.model.Contig3.505    evm.model.Contig32.156    3e-173
## Alignment 192: score=1127.0 e_value=5.5e-78 N=25 ew3&ew6 plus
192-  0: evm.model.Contig3.1145   evm.model.Contig6.21           0
192-  1: evm.model.Contig3.1148   evm.model.Contig6.30       1e-45
192-  2: evm.model.Contig3.1149   evm.model.Contig6.31       2e-28
192-  3: evm.model.Contig3.1150   evm.model.Contig6.36       9e-68
192-  4: evm.model.Contig3.1151   evm.model.Contig6.39           0
192-  5: evm.model.Contig3.1152   evm.model.Contig6.42           0
192-  6: evm.model.Contig3.1153   evm.model.Contig6.46           0
192-  7: evm.model.Contig3.1154   evm.model.Contig6.47           0
192-  8: evm.model.Contig3.1156   evm.model.Contig6.57           0
192-  9: evm.model.Contig3.1160   evm.model.Contig6.59       6e-44
192- 10: evm.model.Contig3.1174   evm.model.Contig6.64           0
192- 11: evm.model.Contig3.1177   evm.model.Contig6.68      5e-162
192- 12: evm.model.Contig3.1180   evm.model.Contig6.71      1e-164
192- 13: evm.model.Contig3.1181   evm.model.Contig6.88           0
192- 14: evm.model.Contig3.1183   evm.model.Contig6.90      2e-149
192- 15: evm.model.Contig3.1184   evm.model.Contig6.91           0
192- 16: evm.model.Contig3.1189   evm.model.Contig6.97       1e-17
192- 17: evm.model.Contig3.1190   evm.model.Contig6.98       3e-68
192- 18: evm.model.Contig3.1191   evm.model.Contig6.99      9e-166
192- 19: evm.model.Contig3.1193   evm.model.Contig6.102          0
```

```
192- 20: evm.model.Contig3.1200   evm.model.Contig6.117     3e-152
192- 21: evm.model.Contig3.1203   evm.model.Contig6.120      5e-23
192- 22: evm.model.Contig3.1206   evm.model.Contig6.129      5e-82
192- 23: evm.model.Contig3.1212   evm.model.Contig6.132      3e-61
192- 24: evm.model.Contig3.1234   evm.model.Contig6.153      8e-37
## Alignment 193: score=510.0 e_value=3.3e-23 N=11 ew3&ew6 plus
193-  0: evm.model.Contig3.1034   evm.model.Contig6.315     2e-168
193-  1: evm.model.Contig3.1038   evm.model.Contig6.321     1e-140
193-  2: evm.model.Contig3.1039   evm.model.Contig6.324     1e-121
193-  3: evm.model.Contig3.1043   evm.model.Contig6.325     5e-109
193-  4: evm.model.Contig3.1044   evm.model.Contig6.327      3e-87
193-  5: evm.model.Contig3.1045   evm.model.Contig6.334          0
193-  6: evm.model.Contig3.1051   evm.model.Contig6.354     9e-100
193-  7: evm.model.Contig3.1053   evm.model.Contig6.355          0
193-  8: evm.model.Contig3.1056   evm.model.Contig6.356      2e-35
193-  9: evm.model.Contig3.1057   evm.model.Contig6.358          0
193- 10: evm.model.Contig3.1058   evm.model.Contig6.359     4e-153
## Alignment 194: score=421.0 e_value=9.9e-17 N=9 ew3&ew6 plus
194-  0: evm.model.Contig3.680    evm.model.Contig6.719     2e-115
194-  1: evm.model.Contig3.686    evm.model.Contig6.724      3e-26
194-  2: evm.model.Contig3.687    evm.model.Contig6.725      1e-24
194-  3: evm.model.Contig3.688    evm.model.Contig6.726     3e-177
194-  4: evm.model.Contig3.690    evm.model.Contig6.728      5e-73
194-  5: evm.model.Contig3.691    evm.model.Contig6.732          0
194-  6: evm.model.Contig3.694    evm.model.Contig6.734      7e-39
194-  7: evm.model.Contig3.699    evm.model.Contig6.749     8e-165
194-  8: evm.model.Contig3.703    evm.model.Contig6.754      4e-40
## Alignment 195: score=336.0 e_value=7.8e-15 N=8 ew3&ew6 plus
195-  0: evm.model.Contig3.308    evm.model.Contig6.988          0
195-  1: evm.model.Contig3.311    evm.model.Contig6.991      1e-84
195-  2: evm.model.Contig3.331    evm.model.Contig6.995     1e-139
195-  3: evm.model.Contig3.334    evm.model.Contig6.1000    9e-162
195-  4: evm.model.Contig3.355    evm.model.Contig6.1009    9e-122
195-  5: evm.model.Contig3.360    evm.model.Contig6.1012    1e-159
195-  6: evm.model.Contig3.361    evm.model.Contig6.1014     2e-80
195-  7: evm.model.Contig3.376    evm.model.Contig6.1018     6e-32
## Alignment 196: score=282.0 e_value=2.2e-08 N=6 ew3&ew6 plus
196-  0: evm.model.Contig3.757    evm.model.Contig6.661          0
196-  1: evm.model.Contig3.759    evm.model.Contig6.662     2e-116
196-  2: evm.model.Contig3.761    evm.model.Contig6.667     6e-127
196-  3: evm.model.Contig3.769    evm.model.Contig6.674      8e-81
196-  4: evm.model.Contig3.771    evm.model.Contig6.676      1e-57
196-  5: evm.model.Contig3.777    evm.model.Contig6.680      4e-53
## Alignment 197: score=257.0 e_value=3.6e-13 N=6 ew3&ew6 plus
197-  0: evm.model.Contig3.928    evm.model.Contig6.449     3e-113
197-  1: evm.model.Contig3.929    evm.model.Contig6.450      7e-74
197-  2: evm.model.Contig3.930    evm.model.Contig6.451      6e-17
197-  3: evm.model.Contig3.931    evm.model.Contig6.454      5e-33
197-  4: evm.model.Contig3.938    evm.model.Contig6.478      2e-47
197-  5: evm.model.Contig3.957    evm.model.Contig6.497      6e-83
## Alignment 198: score=2166.0 e_value=2.7e-172 N=48 ew3&ew6 minus
198-  0: evm.model.Contig3.774    evm.model.Contig6.682      3e-56
198-  1: evm.model.Contig3.782    evm.model.Contig6.667     1e-126
198-  2: evm.model.Contig3.783    evm.model.Contig6.661          0
```

```
198-  3: evm.model.Contig3.785    evm.model.Contig6.651     5e-105
198-  4: evm.model.Contig3.786    evm.model.Contig6.649          0
198-  5: evm.model.Contig3.788    evm.model.Contig6.647      2e-80
198-  6: evm.model.Contig3.789    evm.model.Contig6.646      8e-79
198-  7: evm.model.Contig3.795    evm.model.Contig6.643          0
198-  8: evm.model.Contig3.798    evm.model.Contig6.640     3e-149
198-  9: evm.model.Contig3.799    evm.model.Contig6.637      2e-73
198- 10: evm.model.Contig3.801    evm.model.Contig6.628          0
198- 11: evm.model.Contig3.808    evm.model.Contig6.626      2e-42
198- 12: evm.model.Contig3.814    evm.model.Contig6.620     2e-100
198- 13: evm.model.Contig3.819    evm.model.Contig6.610          0
198- 14: evm.model.Contig3.828    evm.model.Contig6.598      5e-13
198- 15: evm.model.Contig3.829    evm.model.Contig6.597     1e-123
198- 16: evm.model.Contig3.836    evm.model.Contig6.586      2e-54
198- 17: evm.model.Contig3.837    evm.model.Contig6.573      7e-39
198- 18: evm.model.Contig3.839    evm.model.Contig6.572          0
198- 19: evm.model.Contig3.842    evm.model.Contig6.571     2e-146
198- 20: evm.model.Contig3.847    evm.model.Contig6.564     2e-105
198- 21: evm.model.Contig3.849    evm.model.Contig6.559          0
198- 22: evm.model.Contig3.852    evm.model.Contig6.553          0
198- 23: evm.model.Contig3.855    evm.model.Contig6.550          0
198- 24: evm.model.Contig3.862    evm.model.Contig6.543          0
198- 25: evm.model.Contig3.865    evm.model.Contig6.538      2e-87
198- 26: evm.model.Contig3.873    evm.model.Contig6.529     1e-172
198- 27: evm.model.Contig3.875    evm.model.Contig6.524          0
198- 28: evm.model.Contig3.886    evm.model.Contig6.522     2e-156
198- 29: evm.model.Contig3.888    evm.model.Contig6.518      9e-22
198- 30: evm.model.Contig3.890    evm.model.Contig6.517          0
198- 31: evm.model.Contig3.892    evm.model.Contig6.515          0
198- 32: evm.model.Contig3.893    evm.model.Contig6.514      1e-48
198- 33: evm.model.Contig3.904    evm.model.Contig6.513      9e-43
198- 34: evm.model.Contig3.905    evm.model.Contig6.511     2e-138
198- 35: evm.model.Contig3.909    evm.model.Contig6.508     6e-137
198- 36: evm.model.Contig3.913    evm.model.Contig6.504     2e-116
198- 37: evm.model.Contig3.914    evm.model.Contig6.503          0
198- 38: evm.model.Contig3.917    evm.model.Contig6.501     1e-114
198- 39: evm.model.Contig3.922    evm.model.Contig6.499          0
198- 40: evm.model.Contig3.924    evm.model.Contig6.496      5e-83
198- 41: evm.model.Contig3.932    evm.model.Contig6.484      1e-60
198- 42: evm.model.Contig3.958    evm.model.Contig6.474      4e-22
198- 43: evm.model.Contig3.964    evm.model.Contig6.468          0
198- 44: evm.model.Contig3.965    evm.model.Contig6.466      7e-39
198- 45: evm.model.Contig3.970    evm.model.Contig6.464      5e-12
198- 46: evm.model.Contig3.974    evm.model.Contig6.461      5e-59
198- 47: evm.model.Contig3.978    evm.model.Contig6.459          0
## Alignment 199: score=1640.0 e_value=1.2e-122 N=36 ew3&ew6 minus
199-  0: evm.model.Contig3.631    evm.model.Contig6.846          0
199-  1: evm.model.Contig3.632    evm.model.Contig6.845      5e-52
199-  2: evm.model.Contig3.634    evm.model.Contig6.844          0
199-  3: evm.model.Contig3.635    evm.model.Contig6.843      2e-87
199-  4: evm.model.Contig3.636    evm.model.Contig6.842          0
199-  5: evm.model.Contig3.642    evm.model.Contig6.833          0
199-  6: evm.model.Contig3.647    evm.model.Contig6.829      5e-67
199-  7: evm.model.Contig3.650    evm.model.Contig6.828          0
```

```
199-  8: evm.model.Contig3.651    evm.model.Contig6.826      9e-37
199-  9: evm.model.Contig3.653    evm.model.Contig6.821      9e-22
199- 10: evm.model.Contig3.664    evm.model.Contig6.799      8e-80
199- 11: evm.model.Contig3.666    evm.model.Contig6.795          0
199- 12: evm.model.Contig3.668    evm.model.Contig6.793          0
199- 13: evm.model.Contig3.681    evm.model.Contig6.789          0
199- 14: evm.model.Contig3.682    evm.model.Contig6.787      5e-88
199- 15: evm.model.Contig3.683    evm.model.Contig6.786     2e-114
199- 16: evm.model.Contig3.685    evm.model.Contig6.784      3e-45
199- 17: evm.model.Contig3.686    evm.model.Contig6.780      3e-24
199- 18: evm.model.Contig3.687    evm.model.Contig6.778      2e-55
199- 19: evm.model.Contig3.688    evm.model.Contig6.775          0
199- 20: evm.model.Contig3.691    evm.model.Contig6.765      2e-49
199- 21: evm.model.Contig3.694    evm.model.Contig6.760      6e-70
199- 22: evm.model.Contig3.698    evm.model.Contig6.752     2e-151
199- 23: evm.model.Contig3.699    evm.model.Contig6.742          0
199- 24: evm.model.Contig3.709    evm.model.Contig6.716          0
199- 25: evm.model.Contig3.714    evm.model.Contig6.704          0
199- 26: evm.model.Contig3.717    evm.model.Contig6.702          0
199- 27: evm.model.Contig3.723    evm.model.Contig6.701          0
199- 28: evm.model.Contig3.724    evm.model.Contig6.700      2e-47
199- 29: evm.model.Contig3.735    evm.model.Contig6.690     4e-158
199- 30: evm.model.Contig3.738    evm.model.Contig6.686      6e-94
199- 31: evm.model.Contig3.739    evm.model.Contig6.685      2e-88
199- 32: evm.model.Contig3.746    evm.model.Contig6.680      9e-55
199- 33: evm.model.Contig3.750    evm.model.Contig6.677          0
199- 34: evm.model.Contig3.751    evm.model.Contig6.676          0
199- 35: evm.model.Contig3.753    evm.model.Contig6.674      8e-81
## Alignment 200: score=923.0 e_value=6.4e-56 N=20 ew3&ew6 minus
200-  0: evm.model.Contig3.1214   evm.model.Contig6.163          0
200-  1: evm.model.Contig3.1219   evm.model.Contig6.162      5e-41
200-  2: evm.model.Contig3.1231   evm.model.Contig6.158     6e-155
200-  3: evm.model.Contig3.1239   evm.model.Contig6.149     6e-133
200-  4: evm.model.Contig3.1244   evm.model.Contig6.142      2e-84
200-  5: evm.model.Contig3.1246   evm.model.Contig6.141          0
200-  6: evm.model.Contig3.1247   evm.model.Contig6.136      6e-32
200-  7: evm.model.Contig3.1248   evm.model.Contig6.133      3e-60
200-  8: evm.model.Contig3.1252   evm.model.Contig6.126      6e-61
200-  9: evm.model.Contig3.1260   evm.model.Contig6.120     1e-113
200- 10: evm.model.Contig3.1263   evm.model.Contig6.117      2e-74
200- 11: evm.model.Contig3.1264   evm.model.Contig6.108     2e-141
200- 12: evm.model.Contig3.1272   evm.model.Contig6.102          0
200- 13: evm.model.Contig3.1275   evm.model.Contig6.99           0
200- 14: evm.model.Contig3.1276   evm.model.Contig6.98       5e-82
200- 15: evm.model.Contig3.1282   evm.model.Contig6.91           0
200- 16: evm.model.Contig3.1283   evm.model.Contig6.90           0
200- 17: evm.model.Contig3.1286   evm.model.Contig6.89      2e-171
200- 18: evm.model.Contig3.1287   evm.model.Contig6.88           0
200- 19: evm.model.Contig3.1289   evm.model.Contig6.87       1e-13
## Alignment 201: score=799.0 e_value=9.9e-47 N=17 ew3&ew6 minus
201-  0: evm.model.Contig3.1290   evm.model.Contig6.300      9e-58
201-  1: evm.model.Contig3.1291   evm.model.Contig6.299      3e-19
201-  2: evm.model.Contig3.1293   evm.model.Contig6.296          0
201-  3: evm.model.Contig3.1295   evm.model.Contig6.295     1e-137
```

```
201-  4: evm.model.Contig3.1297   evm.model.Contig6.294      2e-98
201-  5: evm.model.Contig3.1300   evm.model.Contig6.288     2e-155
201-  6: evm.model.Contig3.1306   evm.model.Contig6.286          0
201-  7: evm.model.Contig3.1307   evm.model.Contig6.283      1e-50
201-  8: evm.model.Contig3.1308   evm.model.Contig6.282     2e-166
201-  9: evm.model.Contig3.1310   evm.model.Contig6.281     1e-101
201- 10: evm.model.Contig3.1314   evm.model.Contig6.278          0
201- 11: evm.model.Contig3.1317   evm.model.Contig6.276      9e-80
201- 12: evm.model.Contig3.1318   evm.model.Contig6.275          0
201- 13: evm.model.Contig3.1321   evm.model.Contig6.270      2e-24
201- 14: evm.model.Contig3.1328   evm.model.Contig6.266      5e-31
201- 15: evm.model.Contig3.1343   evm.model.Contig6.263      5e-57
201- 16: evm.model.Contig3.1348   evm.model.Contig6.257          0
## Alignment 202: score=738.0 e_value=8.1e-45 N=17 ew3&ew6 minus
202-  0: evm.model.Contig3.928    evm.model.Contig6.458     2e-109
202-  1: evm.model.Contig3.945    evm.model.Contig6.438      2e-48
202-  2: evm.model.Contig3.952    evm.model.Contig6.432     3e-121
202-  3: evm.model.Contig3.972    evm.model.Contig6.427      5e-17
202-  4: evm.model.Contig3.977    evm.model.Contig6.426      6e-33
202-  5: evm.model.Contig3.983    evm.model.Contig6.423     2e-113
202-  6: evm.model.Contig3.988    evm.model.Contig6.420      2e-26
202-  7: evm.model.Contig3.997    evm.model.Contig6.414          0
202-  8: evm.model.Contig3.1001   evm.model.Contig6.411      3e-16
202-  9: evm.model.Contig3.1003   evm.model.Contig6.407          0
202- 10: evm.model.Contig3.1004   evm.model.Contig6.398      1e-50
202- 11: evm.model.Contig3.1005   evm.model.Contig6.394          0
202- 12: evm.model.Contig3.1010   evm.model.Contig6.380          0
202- 13: evm.model.Contig3.1011   evm.model.Contig6.379          0
202- 14: evm.model.Contig3.1017   evm.model.Contig6.367      2e-33
202- 15: evm.model.Contig3.1020   evm.model.Contig6.366      4e-74
202- 16: evm.model.Contig3.1022   evm.model.Contig6.361          0
## Alignment 203: score=620.0 e_value=6.8e-37 N=14 ew3&ew6 minus
203-  0: evm.model.Contig3.328    evm.model.Contig6.990      1e-13
203-  1: evm.model.Contig3.335    evm.model.Contig6.986          0
203-  2: evm.model.Contig3.345    evm.model.Contig6.979     2e-158
203-  3: evm.model.Contig3.348    evm.model.Contig6.977      6e-37
203-  4: evm.model.Contig3.360    evm.model.Contig6.970     3e-164
203-  5: evm.model.Contig3.364    evm.model.Contig6.969          0
203-  6: evm.model.Contig3.371    evm.model.Contig6.963     1e-131
203-  7: evm.model.Contig3.372    evm.model.Contig6.962      9e-14
203-  8: evm.model.Contig3.373    evm.model.Contig6.956     2e-176
203-  9: evm.model.Contig3.378    evm.model.Contig6.950      1e-53
203- 10: evm.model.Contig3.379    evm.model.Contig6.949     4e-160
203- 11: evm.model.Contig3.382    evm.model.Contig6.947     2e-139
203- 12: evm.model.Contig3.383    evm.model.Contig6.939      8e-97
203- 13: evm.model.Contig3.408    evm.model.Contig6.914     2e-116
## Alignment 204: score=506.0 e_value=1.4e-24 N=11 ew3&ew6 minus
204-  0: evm.model.Contig3.1077   evm.model.Contig6.222          0
204-  1: evm.model.Contig3.1090   evm.model.Contig6.213      3e-77
204-  2: evm.model.Contig3.1092   evm.model.Contig6.212          0
204-  3: evm.model.Contig3.1093   evm.model.Contig6.204      9e-59
204-  4: evm.model.Contig3.1094   evm.model.Contig6.203      7e-86
204-  5: evm.model.Contig3.1097   evm.model.Contig6.202      6e-33
204-  6: evm.model.Contig3.1099   evm.model.Contig6.198      2e-84
```

```
204-  7: evm.model.Contig3.1104   evm.model.Contig6.194     5e-150
204-  8: evm.model.Contig3.1107   evm.model.Contig6.189      5e-21
204-  9: evm.model.Contig3.1116   evm.model.Contig6.180      3e-27
204- 10: evm.model.Contig3.1119   evm.model.Contig6.176          0
## Alignment 205: score=261.0 e_value=6.6e-11 N=6 ew3&ew6 minus
205-  0: evm.model.Contig3.1031   evm.model.Contig6.334          0
205-  1: evm.model.Contig3.1032   evm.model.Contig6.327      1e-82
205-  2: evm.model.Contig3.1033   evm.model.Contig6.325      1e-98
205-  3: evm.model.Contig3.1040   evm.model.Contig6.319     3e-176
205-  4: evm.model.Contig3.1065   evm.model.Contig6.307          0
205-  5: evm.model.Contig3.1068   evm.model.Contig6.306      6e-60
## Alignment 206: score=269.0 e_value=4.8e-08 N=6 ew30&ew30 plus
206-  0: evm.model.Contig30.452   evm.model.Contig30.531    4e-141
206-  1: evm.model.Contig30.455   evm.model.Contig30.532         0
206-  2: evm.model.Contig30.461   evm.model.Contig30.534         0
206-  3: evm.model.Contig30.475   evm.model.Contig30.540     1e-68
206-  4: evm.model.Contig30.484   evm.model.Contig30.548         0
206-  5: evm.model.Contig30.488   evm.model.Contig30.552         0
## Alignment 207: score=320.0 e_value=3.6e-15 N=7 ew30&ew30 minus
207-  0: evm.model.Contig30.474   evm.model.Contig30.543         0
207-  1: evm.model.Contig30.476   evm.model.Contig30.539         0
207-  2: evm.model.Contig30.477   evm.model.Contig30.538    5e-137
207-  3: evm.model.Contig30.478   evm.model.Contig30.537         0
207-  4: evm.model.Contig30.504   evm.model.Contig30.513    9e-115
207-  5: evm.model.Contig30.506   evm.model.Contig30.512         0
207-  6: evm.model.Contig30.508   evm.model.Contig30.511         0
## Alignment 208: score=746.0 e_value=7.5e-50 N=18 ew30&ew40 plus
208-  0: evm.model.Contig30.182   evm.model.Contig40.269     5e-37
208-  1: evm.model.Contig30.194   evm.model.Contig40.275     8e-13
208-  2: evm.model.Contig30.195   evm.model.Contig40.277         0
208-  3: evm.model.Contig30.201   evm.model.Contig40.288     7e-40
208-  4: evm.model.Contig30.205   evm.model.Contig40.298     1e-38
208-  5: evm.model.Contig30.207   evm.model.Contig40.300     3e-18
208-  6: evm.model.Contig30.208   evm.model.Contig40.302     2e-76
208-  7: evm.model.Contig30.228   evm.model.Contig40.327    7e-131
208-  8: evm.model.Contig30.240   evm.model.Contig40.333     9e-24
208-  9: evm.model.Contig30.253   evm.model.Contig40.340    4e-144
208- 10: evm.model.Contig30.260   evm.model.Contig40.344         0
208- 11: evm.model.Contig30.263   evm.model.Contig40.347     6e-58
208- 12: evm.model.Contig30.270   evm.model.Contig40.352         0
208- 13: evm.model.Contig30.274   evm.model.Contig40.353     2e-45
208- 14: evm.model.Contig30.298   evm.model.Contig40.356     1e-47
208- 15: evm.model.Contig30.308   evm.model.Contig40.358     8e-75
208- 16: evm.model.Contig30.313   evm.model.Contig40.359         0
208- 17: evm.model.Contig30.335   evm.model.Contig40.370         0
## Alignment 209: score=278.0 e_value=6.6e-10 N=6 ew30&ew40 plus
209-  0: evm.model.Contig30.136   evm.model.Contig40.179     4e-46
209-  1: evm.model.Contig30.147   evm.model.Contig40.185         0
209-  2: evm.model.Contig30.158   evm.model.Contig40.190     8e-81
209-  3: evm.model.Contig30.160   evm.model.Contig40.192     3e-85
209-  4: evm.model.Contig30.161   evm.model.Contig40.193    3e-154
209-  5: evm.model.Contig30.162   evm.model.Contig40.195         0
## Alignment 210: score=289.0 e_value=1.1e-10 N=7 ew30&ew40 minus
210-  0: evm.model.Contig30.384   evm.model.Contig40.227     2e-39
```

```
210-  1: evm.model.Contig30.390   evm.model.Contig40.226     1e-39
210-  2: evm.model.Contig30.409   evm.model.Contig40.225    3e-113
210-  3: evm.model.Contig30.419   evm.model.Contig40.221     6e-33
210-  4: evm.model.Contig30.424   evm.model.Contig40.218    7e-133
210-  5: evm.model.Contig30.443   evm.model.Contig40.205     2e-38
210-  6: evm.model.Contig30.451   evm.model.Contig40.202     3e-31
## Alignment 211: score=278.0 e_value=3.5e-08 N=6 ew30&ew40 minus
211-  0: evm.model.Contig30.104   evm.model.Contig40.131     7e-88
211-  1: evm.model.Contig30.106   evm.model.Contig40.124    1e-149
211-  2: evm.model.Contig30.107   evm.model.Contig40.121    3e-127
211-  3: evm.model.Contig30.116   evm.model.Contig40.112         0
211-  4: evm.model.Contig30.119   evm.model.Contig40.111     1e-71
211-  5: evm.model.Contig30.122   evm.model.Contig40.106     3e-29
## Alignment 212: score=299.0 e_value=1.1e-07 N=6 ew31&ew31 plus
212-  0: evm.model.Contig31.563   evm.model.Contig31.630     2e-85
212-  1: evm.model.Contig31.564   evm.model.Contig31.631         0
212-  2: evm.model.Contig31.565   evm.model.Contig31.632    7e-140
212-  3: evm.model.Contig31.566   evm.model.Contig31.633         0
212-  4: evm.model.Contig31.568   evm.model.Contig31.634    2e-163
212-  5: evm.model.Contig31.569   evm.model.Contig31.635         0
## Alignment 213: score=282.0 e_value=3.4e-08 N=6 ew31&ew31 plus
213-  0: evm.model.Contig31.481   evm.model.Contig31.616     2e-87
213-  1: evm.model.Contig31.489   evm.model.Contig31.621         0
213-  2: evm.model.Contig31.490   evm.model.Contig31.622         0
213-  3: evm.model.Contig31.494   evm.model.Contig31.623         0
213-  4: evm.model.Contig31.497   evm.model.Contig31.625         0
213-  5: evm.model.Contig31.504   evm.model.Contig31.629         0
## Alignment 214: score=989.0 e_value=1.2e-58 N=21 ew31&ew31 minus
214-  0: evm.model.Contig31.240   evm.model.Contig31.368     6e-33
214-  1: evm.model.Contig31.242   evm.model.Contig31.367         0
214-  2: evm.model.Contig31.245   evm.model.Contig31.366    3e-159
214-  3: evm.model.Contig31.246   evm.model.Contig31.365    7e-174
214-  4: evm.model.Contig31.248   evm.model.Contig31.364         0
214-  5: evm.model.Contig31.249   evm.model.Contig31.361    2e-108
214-  6: evm.model.Contig31.253   evm.model.Contig31.360    6e-109
214-  7: evm.model.Contig31.260   evm.model.Contig31.358     6e-93
214-  8: evm.model.Contig31.270   evm.model.Contig31.353         0
214-  9: evm.model.Contig31.271   evm.model.Contig31.352    1e-162
214- 10: evm.model.Contig31.286   evm.model.Contig31.350         0
214- 11: evm.model.Contig31.291   evm.model.Contig31.348         0
214- 12: evm.model.Contig31.297   evm.model.Contig31.343         0
214- 13: evm.model.Contig31.298   evm.model.Contig31.341         0
214- 14: evm.model.Contig31.307   evm.model.Contig31.338    2e-162
214- 15: evm.model.Contig31.308   evm.model.Contig31.337         0
214- 16: evm.model.Contig31.309   evm.model.Contig31.336     3e-66
214- 17: evm.model.Contig31.312   evm.model.Contig31.334         0
214- 18: evm.model.Contig31.314   evm.model.Contig31.332         0
214- 19: evm.model.Contig31.317   evm.model.Contig31.331         0
214- 20: evm.model.Contig31.318   evm.model.Contig31.330     3e-83
## Alignment 215: score=543.0 e_value=4.9e-24 N=11 ew31&ew31 minus
215-  0: evm.model.Contig31.187   evm.model.Contig31.219         0
215-  1: evm.model.Contig31.188   evm.model.Contig31.218     1e-73
215-  2: evm.model.Contig31.189   evm.model.Contig31.217         0
215-  3: evm.model.Contig31.193   evm.model.Contig31.215         0
```

```
215-  4: evm.model.Contig31.194   evm.model.Contig31.214        0
215-  5: evm.model.Contig31.195   evm.model.Contig31.213        0
215-  6: evm.model.Contig31.196   evm.model.Contig31.211        0
215-  7: evm.model.Contig31.197   evm.model.Contig31.210        0
215-  8: evm.model.Contig31.198   evm.model.Contig31.208        0
215-  9: evm.model.Contig31.199   evm.model.Contig31.207        0
215- 10: evm.model.Contig31.200   evm.model.Contig31.204        0
## Alignment 216: score=359.0 e_value=2.7e-17 N=8 ew31&ew31 minus
216-  0: evm.model.Contig31.116   evm.model.Contig31.185    6e-61
216-  1: evm.model.Contig31.134   evm.model.Contig31.178        0
216-  2: evm.model.Contig31.135   evm.model.Contig31.175        0
216-  3: evm.model.Contig31.137   evm.model.Contig31.170   1e-139
216-  4: evm.model.Contig31.141   evm.model.Contig31.153    1e-53
216-  5: evm.model.Contig31.142   evm.model.Contig31.150   1e-168
216-  6: evm.model.Contig31.143   evm.model.Contig31.149        0
216-  7: evm.model.Contig31.144   evm.model.Contig31.148    9e-77
## Alignment 217: score=270.0 e_value=4.4e-09 N=6 ew31&ew31 minus
217-  0: evm.model.Contig31.272   evm.model.Contig31.355    2e-92
217-  1: evm.model.Contig31.284   evm.model.Contig31.348    1e-58
217-  2: evm.model.Contig31.290   evm.model.Contig31.346   1e-151
217-  3: evm.model.Contig31.293   evm.model.Contig31.342        0
217-  4: evm.model.Contig31.294   evm.model.Contig31.340        0
217-  5: evm.model.Contig31.305   evm.model.Contig31.335   9e-128
## Alignment 218: score=780.0 e_value=3.9e-51 N=18 ew31&ew7 plus
218-  0: evm.model.Contig31.525   evm.model.Contig7.12      2e-72
218-  1: evm.model.Contig31.527   evm.model.Contig7.14     1e-115
218-  2: evm.model.Contig31.536   evm.model.Contig7.24      7e-67
218-  3: evm.model.Contig31.562   evm.model.Contig7.33      1e-14
218-  4: evm.model.Contig31.563   evm.model.Contig7.34          0
218-  5: evm.model.Contig31.570   evm.model.Contig7.38          0
218-  6: evm.model.Contig31.572   evm.model.Contig7.40          0
218-  7: evm.model.Contig31.578   evm.model.Contig7.44      9e-65
218-  8: evm.model.Contig31.583   evm.model.Contig7.47          0
218-  9: evm.model.Contig31.585   evm.model.Contig7.50     6e-143
218- 10: evm.model.Contig31.586   evm.model.Contig7.51      6e-33
218- 11: evm.model.Contig31.587   evm.model.Contig7.52          0
218- 12: evm.model.Contig31.589   evm.model.Contig7.54     2e-108
218- 13: evm.model.Contig31.592   evm.model.Contig7.60      2e-49
218- 14: evm.model.Contig31.595   evm.model.Contig7.61      7e-81
218- 15: evm.model.Contig31.603   evm.model.Contig7.71          0
218- 16: evm.model.Contig31.608   evm.model.Contig7.97      2e-77
218- 17: evm.model.Contig31.624   evm.model.Contig7.123     2e-44
## Alignment 219: score=557.0 e_value=1.1e-29 N=13 ew31&ew7 plus
219-  0: evm.model.Contig31.9     evm.model.Contig7.146     2e-99
219-  1: evm.model.Contig31.13    evm.model.Contig7.156    2e-101
219-  2: evm.model.Contig31.15    evm.model.Contig7.167    2e-151
219-  3: evm.model.Contig31.23    evm.model.Contig7.176     9e-24
219-  4: evm.model.Contig31.30    evm.model.Contig7.177     4e-85
219-  5: evm.model.Contig31.32    evm.model.Contig7.185     3e-11
219-  6: evm.model.Contig31.33    evm.model.Contig7.186    1e-135
219-  7: evm.model.Contig31.37    evm.model.Contig7.195         0
219-  8: evm.model.Contig31.45    evm.model.Contig7.213     2e-40
219-  9: evm.model.Contig31.55    evm.model.Contig7.223    6e-122
219- 10: evm.model.Contig31.59    evm.model.Contig7.226         0
```

```
219- 11: evm.model.Contig31.65    evm.model.Contig7.232      1e-62
219- 12: evm.model.Contig31.77    evm.model.Contig7.238     2e-137
## Alignment 220: score=345.0 e_value=3e-15 N=8 ew31&ew7 plus
220-  0: evm.model.Contig31.319   evm.model.Contig7.398      1e-73
220-  1: evm.model.Contig31.329   evm.model.Contig7.404      3e-11
220-  2: evm.model.Contig31.332   evm.model.Contig7.407     4e-160
220-  3: evm.model.Contig31.334   evm.model.Contig7.410      3e-61
220-  4: evm.model.Contig31.335   evm.model.Contig7.413      8e-18
220-  5: evm.model.Contig31.336   evm.model.Contig7.417      1e-47
220-  6: evm.model.Contig31.338   evm.model.Contig7.432      7e-50
220-  7: evm.model.Contig31.362   evm.model.Contig7.439      4e-76
## Alignment 221: score=331.0 e_value=7.2e-11 N=7 ew31&ew7 plus
221-  0: evm.model.Contig31.444   evm.model.Contig7.270      9e-39
221-  1: evm.model.Contig31.445   evm.model.Contig7.273      8e-37
221-  2: evm.model.Contig31.446   evm.model.Contig7.274     4e-114
221-  3: evm.model.Contig31.452   evm.model.Contig7.279          0
221-  4: evm.model.Contig31.453   evm.model.Contig7.280      4e-24
221-  5: evm.model.Contig31.455   evm.model.Contig7.285     2e-152
221-  6: evm.model.Contig31.459   evm.model.Contig7.294      4e-26
## Alignment 222: score=328.0 e_value=8.5e-12 N=7 ew31&ew7 plus
222-  0: evm.model.Contig31.374   evm.model.Contig7.246     2e-166
222-  1: evm.model.Contig31.381   evm.model.Contig7.251          0
222-  2: evm.model.Contig31.388   evm.model.Contig7.252      2e-40
222-  3: evm.model.Contig31.391   evm.model.Contig7.254      5e-76
222-  4: evm.model.Contig31.392   evm.model.Contig7.259      2e-34
222-  5: evm.model.Contig31.393   evm.model.Contig7.260          0
222-  6: evm.model.Contig31.394   evm.model.Contig7.265      3e-20
## Alignment 223: score=758.0 e_value=2e-45 N=17 ew31&ew7 minus
223-  0: evm.model.Contig31.396   evm.model.Contig7.381          0
223-  1: evm.model.Contig31.398   evm.model.Contig7.379          0
223-  2: evm.model.Contig31.405   evm.model.Contig7.377      2e-15
223-  3: evm.model.Contig31.408   evm.model.Contig7.370     4e-130
223-  4: evm.model.Contig31.410   evm.model.Contig7.363      4e-36
223-  5: evm.model.Contig31.411   evm.model.Contig7.356      2e-21
223-  6: evm.model.Contig31.415   evm.model.Contig7.334      1e-29
223-  7: evm.model.Contig31.416   evm.model.Contig7.330     5e-114
223-  8: evm.model.Contig31.417   evm.model.Contig7.329          0
223-  9: evm.model.Contig31.419   evm.model.Contig7.328      1e-38
223- 10: evm.model.Contig31.420   evm.model.Contig7.326      1e-24
223- 11: evm.model.Contig31.422   evm.model.Contig7.321      3e-36
223- 12: evm.model.Contig31.426   evm.model.Contig7.313          0
223- 13: evm.model.Contig31.430   evm.model.Contig7.303      2e-16
223- 14: evm.model.Contig31.431   evm.model.Contig7.302          0
223- 15: evm.model.Contig31.433   evm.model.Contig7.300      2e-72
223- 16: evm.model.Contig31.454   evm.model.Contig7.291      2e-75
## Alignment 224: score=677.0 e_value=1.7e-37 N=15 ew31&ew7 minus
224-  0: evm.model.Contig31.130   evm.model.Contig7.769     1e-135
224-  1: evm.model.Contig31.147   evm.model.Contig7.750      1e-72
224-  2: evm.model.Contig31.148   evm.model.Contig7.747     5e-143
224-  3: evm.model.Contig31.151   evm.model.Contig7.746     2e-169
224-  4: evm.model.Contig31.154   evm.model.Contig7.744      2e-45
224-  5: evm.model.Contig31.155   evm.model.Contig7.742      5e-60
224-  6: evm.model.Contig31.156   evm.model.Contig7.740          0
224-  7: evm.model.Contig31.159   evm.model.Contig7.738      1e-29
```

```
224-  8: evm.model.Contig31.160   evm.model.Contig7.737          0
224-  9: evm.model.Contig31.162   evm.model.Contig7.736     1e-141
224- 10: evm.model.Contig31.166   evm.model.Contig7.727          0
224- 11: evm.model.Contig31.172   evm.model.Contig7.718     8e-118
224- 12: evm.model.Contig31.181   evm.model.Contig7.713      1e-54
224- 13: evm.model.Contig31.184   evm.model.Contig7.710     4e-112
224- 14: evm.model.Contig31.203   evm.model.Contig7.702     2e-166
## Alignment 225: score=366.0 e_value=5.4e-14 N=8 ew31&ew7 minus
225-  0: evm.model.Contig31.307   evm.model.Contig7.432      7e-50
225-  1: evm.model.Contig31.309   evm.model.Contig7.417      2e-44
225-  2: evm.model.Contig31.312   evm.model.Contig7.411     8e-131
225-  3: evm.model.Contig31.313   evm.model.Contig7.408      2e-33
225-  4: evm.model.Contig31.314   evm.model.Contig7.407     3e-161
225-  5: evm.model.Contig31.315   evm.model.Contig7.405      4e-80
225-  6: evm.model.Contig31.317   evm.model.Contig7.401      5e-73
225-  7: evm.model.Contig31.323   evm.model.Contig7.391          0
## Alignment 226: score=280.0 e_value=5.9e-10 N=6 ew31&ew7 minus
226-  0: evm.model.Contig31.479   evm.model.Contig7.140      2e-19
226-  1: evm.model.Contig31.480   evm.model.Contig7.139     8e-139
226-  2: evm.model.Contig31.484   evm.model.Contig7.134      7e-14
226-  3: evm.model.Contig31.491   evm.model.Contig7.126     2e-135
226-  4: evm.model.Contig31.493   evm.model.Contig7.123      3e-43
226-  5: evm.model.Contig31.501   evm.model.Contig7.119      2e-11
## Alignment 227: score=269.0 e_value=5.1e-10 N=6 ew31&ew7 minus
227-  0: evm.model.Contig31.463   evm.model.Contig7.586      5e-25
227-  1: evm.model.Contig31.465   evm.model.Contig7.583     7e-161
227-  2: evm.model.Contig31.466   evm.model.Contig7.577          0
227-  3: evm.model.Contig31.467   evm.model.Contig7.576      1e-65
227-  4: evm.model.Contig31.469   evm.model.Contig7.575          0
227-  5: evm.model.Contig31.493   evm.model.Contig7.568      3e-69
## Alignment 228: score=267.0 e_value=8.3e-09 N=6 ew31&ew7 minus
228-  0: evm.model.Contig31.93    evm.model.Contig7.630      2e-61
228-  1: evm.model.Contig31.96    evm.model.Contig7.629     2e-134
228-  2: evm.model.Contig31.102   evm.model.Contig7.627      1e-98
228-  3: evm.model.Contig31.103   evm.model.Contig7.621     6e-113
228-  4: evm.model.Contig31.104   evm.model.Contig7.602     8e-157
228-  5: evm.model.Contig31.108   evm.model.Contig7.598          0
## Alignment 229: score=266.0 e_value=2.4e-11 N=7 ew31&ew7 minus
229-  0: evm.model.Contig31.396   evm.model.Contig7.374          0
229-  1: evm.model.Contig31.408   evm.model.Contig7.355     9e-140
229-  2: evm.model.Contig31.410   evm.model.Contig7.345      1e-58
229-  3: evm.model.Contig31.418   evm.model.Contig7.333      3e-63
229-  4: evm.model.Contig31.420   evm.model.Contig7.319      6e-94
229-  5: evm.model.Contig31.433   evm.model.Contig7.313      6e-91
229-  6: evm.model.Contig31.455   evm.model.Contig7.296     8e-155
## Alignment 230: score=491.0 e_value=3.6e-21 N=10 ew33&ew33 plus
230-  0: evm.model.Contig33.8     evm.model.Contig33.149    9e-172
230-  1: evm.model.Contig33.9     evm.model.Contig33.150         0
230-  2: evm.model.Contig33.12    evm.model.Contig33.156         0
230-  3: evm.model.Contig33.13    evm.model.Contig33.157    3e-139
230-  4: evm.model.Contig33.14    evm.model.Contig33.158         0
230-  5: evm.model.Contig33.15    evm.model.Contig33.159    1e-157
230-  6: evm.model.Contig33.16    evm.model.Contig33.160         0
230-  7: evm.model.Contig33.17    evm.model.Contig33.161         0
```

```
230-  8: evm.model.Contig33.18    evm.model.Contig33.166    4e-175
230-  9: evm.model.Contig33.19    evm.model.Contig33.167         0
## Alignment 231: score=260.0 e_value=1.5e-09 N=6 ew33&ew33 plus
231-  0: evm.model.Contig33.188   evm.model.Contig33.231     4e-22
231-  1: evm.model.Contig33.192   evm.model.Contig33.240     3e-45
231-  2: evm.model.Contig33.195   evm.model.Contig33.257         0
231-  3: evm.model.Contig33.196   evm.model.Contig33.258     2e-87
231-  4: evm.model.Contig33.199   evm.model.Contig33.259     9e-29
231-  5: evm.model.Contig33.201   evm.model.Contig33.274         0
## Alignment 232: score=392.0 e_value=4.2e-15 N=8 ew33&ew33 minus
232-  0: evm.model.Contig33.518   evm.model.Contig33.547     5e-89
232-  1: evm.model.Contig33.523   evm.model.Contig33.546    2e-138
232-  2: evm.model.Contig33.524   evm.model.Contig33.545    8e-137
232-  3: evm.model.Contig33.526   evm.model.Contig33.544         0
232-  4: evm.model.Contig33.527   evm.model.Contig33.543    1e-125
232-  5: evm.model.Contig33.528   evm.model.Contig33.542         0
232-  6: evm.model.Contig33.529   evm.model.Contig33.541         0
232-  7: evm.model.Contig33.530   evm.model.Contig33.537         0
## Alignment 233: score=374.0 e_value=1.5e-16 N=8 ew33&ew33 minus
233-  0: evm.model.Contig33.199   evm.model.Contig33.259     9e-29
233-  1: evm.model.Contig33.206   evm.model.Contig33.251    4e-161
233-  2: evm.model.Contig33.218   evm.model.Contig33.240     1e-68
233-  3: evm.model.Contig33.219   evm.model.Contig33.239     1e-96
233-  4: evm.model.Contig33.220   evm.model.Contig33.237    2e-106
233-  5: evm.model.Contig33.221   evm.model.Contig33.234         0
233-  6: evm.model.Contig33.222   evm.model.Contig33.233         0
233-  7: evm.model.Contig33.228   evm.model.Contig33.229     3e-44
## Alignment 234: score=314.0 e_value=2.3e-12 N=7 ew33&ew33 minus
234-  0: evm.model.Contig33.177   evm.model.Contig33.249         0
234-  1: evm.model.Contig33.179   evm.model.Contig33.248         0
234-  2: evm.model.Contig33.181   evm.model.Contig33.246         0
234-  3: evm.model.Contig33.182   evm.model.Contig33.245     2e-28
234-  4: evm.model.Contig33.188   evm.model.Contig33.231     4e-22
234-  5: evm.model.Contig33.192   evm.model.Contig33.218     4e-16
234-  6: evm.model.Contig33.202   evm.model.Contig33.214     6e-15
## Alignment 235: score=295.0 e_value=4.6e-09 N=6 ew33&ew33 minus
235-  0: evm.model.Contig33.309   evm.model.Contig33.332         0
235-  1: evm.model.Contig33.311   evm.model.Contig33.329     3e-80
235-  2: evm.model.Contig33.312   evm.model.Contig33.328     4e-18
235-  3: evm.model.Contig33.313   evm.model.Contig33.327     6e-15
235-  4: evm.model.Contig33.314   evm.model.Contig33.325         0
235-  5: evm.model.Contig33.315   evm.model.Contig33.322    3e-121
## Alignment 236: score=585.0 e_value=3.5e-33 N=13 ew34&ew5 plus
236-  0: evm.model.Contig34.427   evm.model.Contig5.946      7e-36
236-  1: evm.model.Contig34.430   evm.model.Contig5.947          0
236-  2: evm.model.Contig34.451   evm.model.Contig5.953          0
236-  3: evm.model.Contig34.452   evm.model.Contig5.954      8e-55
236-  4: evm.model.Contig34.460   evm.model.Contig5.955     6e-174
236-  5: evm.model.Contig34.462   evm.model.Contig5.965          0
236-  6: evm.model.Contig34.463   evm.model.Contig5.968          0
236-  7: evm.model.Contig34.470   evm.model.Contig5.970          0
236-  8: evm.model.Contig34.471   evm.model.Contig5.971          0
236-  9: evm.model.Contig34.484   evm.model.Contig5.972     1e-137
236- 10: evm.model.Contig34.487   evm.model.Contig5.973      4e-81
```

```
236- 11: evm.model.Contig34.489   evm.model.Contig5.977         0
236- 12: evm.model.Contig34.492   evm.model.Contig5.978     2e-29
## Alignment 237: score=495.0 e_value=1.9e-23 N=11 ew34&ew5 plus
237-  0: evm.model.Contig34.215   evm.model.Contig5.821     7e-52
237-  1: evm.model.Contig34.237   evm.model.Contig5.822         0
237-  2: evm.model.Contig34.243   evm.model.Contig5.825    1e-172
237-  3: evm.model.Contig34.245   evm.model.Contig5.827         0
237-  4: evm.model.Contig34.247   evm.model.Contig5.832         0
237-  5: evm.model.Contig34.253   evm.model.Contig5.838     2e-80
237-  6: evm.model.Contig34.255   evm.model.Contig5.839     4e-88
237-  7: evm.model.Contig34.256   evm.model.Contig5.841    1e-136
237-  8: evm.model.Contig34.257   evm.model.Contig5.843     6e-72
237-  9: evm.model.Contig34.265   evm.model.Contig5.847         0
237- 10: evm.model.Contig34.271   evm.model.Contig5.857         0
## Alignment 238: score=440.0 e_value=1.1e-20 N=10 ew34&ew5 plus
238-  0: evm.model.Contig34.368   evm.model.Contig5.766     3e-67
238-  1: evm.model.Contig34.373   evm.model.Contig5.768     3e-69
238-  2: evm.model.Contig34.374   evm.model.Contig5.770     2e-59
238-  3: evm.model.Contig34.378   evm.model.Contig5.777         0
238-  4: evm.model.Contig34.380   evm.model.Contig5.778     2e-25
238-  5: evm.model.Contig34.385   evm.model.Contig5.780     6e-14
238-  6: evm.model.Contig34.392   evm.model.Contig5.784     3e-63
238-  7: evm.model.Contig34.398   evm.model.Contig5.790     1e-50
238-  8: evm.model.Contig34.414   evm.model.Contig5.793    2e-122
238-  9: evm.model.Contig34.416   evm.model.Contig5.812    1e-122
## Alignment 239: score=393.0 e_value=2.7e-18 N=9 ew34&ew5 plus
239-  0: evm.model.Contig34.84    evm.model.Contig5.672         0
239-  1: evm.model.Contig34.88    evm.model.Contig5.678    2e-131
239-  2: evm.model.Contig34.89    evm.model.Contig5.682         0
239-  3: evm.model.Contig34.107   evm.model.Contig5.696         0
239-  4: evm.model.Contig34.115   evm.model.Contig5.698     3e-72
239-  5: evm.model.Contig34.116   evm.model.Contig5.702     1e-54
239-  6: evm.model.Contig34.120   evm.model.Contig5.710     2e-95
239-  7: evm.model.Contig34.125   evm.model.Contig5.722         0
239-  8: evm.model.Contig34.126   evm.model.Contig5.727         0
## Alignment 240: score=384.0 e_value=9.1e-19 N=9 ew34&ew5 plus
240-  0: evm.model.Contig34.29    evm.model.Contig5.575     8e-92
240-  1: evm.model.Contig34.31    evm.model.Contig5.578         0
240-  2: evm.model.Contig34.35    evm.model.Contig5.581     3e-58
240-  3: evm.model.Contig34.50    evm.model.Contig5.583         0
240-  4: evm.model.Contig34.55    evm.model.Contig5.586     2e-44
240-  5: evm.model.Contig34.62    evm.model.Contig5.597         0
240-  6: evm.model.Contig34.63    evm.model.Contig5.598    5e-157
240-  7: evm.model.Contig34.75    evm.model.Contig5.620     3e-31
240-  8: evm.model.Contig34.88    evm.model.Contig5.623    6e-120
## Alignment 241: score=362.0 e_value=3.9e-14 N=8 ew34&ew5 minus
241-  0: evm.model.Contig34.103   evm.model.Contig5.1030   4e-155
241-  1: evm.model.Contig34.104   evm.model.Contig5.1029    4e-60
241-  2: evm.model.Contig34.107   evm.model.Contig5.1026        0
241-  3: evm.model.Contig34.120   evm.model.Contig5.1016    5e-96
241-  4: evm.model.Contig34.124   evm.model.Contig5.1008        0
241-  5: evm.model.Contig34.132   evm.model.Contig5.994         0
241-  6: evm.model.Contig34.136   evm.model.Contig5.993     3e-18
241-  7: evm.model.Contig34.138   evm.model.Contig5.992     3e-57
```

```
## Alignment 242: score=250.0 e_value=2e-12 N=7 ew34&ew5 minus
242-  0: evm.model.Contig34.285   evm.model.Contig5.959      3e-56
242-  1: evm.model.Contig34.286   evm.model.Contig5.940      2e-53
242-  2: evm.model.Contig34.306   evm.model.Contig5.914          0
242-  3: evm.model.Contig34.312   evm.model.Contig5.913      5e-78
242-  4: evm.model.Contig34.330   evm.model.Contig5.908     5e-177
242-  5: evm.model.Contig34.334   evm.model.Contig5.895          0
242-  6: evm.model.Contig34.358   evm.model.Contig5.872      1e-96
## Alignment 243: score=757.0 e_value=3.3e-50 N=17 ew35&ew5 plus
243-  0: evm.model.Contig35.356   evm.model.Contig5.255      1e-46
243-  1: evm.model.Contig35.357   evm.model.Contig5.256          0
243-  2: evm.model.Contig35.358   evm.model.Contig5.259      2e-92
243-  3: evm.model.Contig35.359   evm.model.Contig5.261          0
243-  4: evm.model.Contig35.361   evm.model.Contig5.263     7e-130
243-  5: evm.model.Contig35.367   evm.model.Contig5.264     8e-139
243-  6: evm.model.Contig35.389   evm.model.Contig5.266          0
243-  7: evm.model.Contig35.399   evm.model.Contig5.289      1e-75
243-  8: evm.model.Contig35.403   evm.model.Contig5.292      9e-81
243-  9: evm.model.Contig35.409   evm.model.Contig5.306      6e-33
243- 10: evm.model.Contig35.411   evm.model.Contig5.310      6e-58
243- 11: evm.model.Contig35.412   evm.model.Contig5.314      1e-24
243- 12: evm.model.Contig35.431   evm.model.Contig5.332      4e-37
243- 13: evm.model.Contig35.432   evm.model.Contig5.333     4e-105
243- 14: evm.model.Contig35.433   evm.model.Contig5.334          0
243- 15: evm.model.Contig35.434   evm.model.Contig5.335          0
243- 16: evm.model.Contig35.436   evm.model.Contig5.336      1e-20
## Alignment 244: score=484.0 e_value=4.4e-24 N=11 ew35&ew5 plus
244-  0: evm.model.Contig35.417   evm.model.Contig5.383      3e-14
244-  1: evm.model.Contig35.437   evm.model.Contig5.394     3e-177
244-  2: evm.model.Contig35.439   evm.model.Contig5.395          0
244-  3: evm.model.Contig35.444   evm.model.Contig5.396      9e-24
244-  4: evm.model.Contig35.450   evm.model.Contig5.407      1e-92
244-  5: evm.model.Contig35.453   evm.model.Contig5.408      7e-15
244-  6: evm.model.Contig35.454   evm.model.Contig5.409     1e-124
244-  7: evm.model.Contig35.456   evm.model.Contig5.416     2e-117
244-  8: evm.model.Contig35.459   evm.model.Contig5.417      9e-27
244-  9: evm.model.Contig35.470   evm.model.Contig5.423     3e-148
244- 10: evm.model.Contig35.483   evm.model.Contig5.430      2e-65
## Alignment 245: score=277.0 e_value=5.6e-10 N=6 ew35&ew5 plus
245-  0: evm.model.Contig35.310   evm.model.Contig5.101          0
245-  1: evm.model.Contig35.326   evm.model.Contig5.106      9e-78
245-  2: evm.model.Contig35.329   evm.model.Contig5.108      2e-13
245-  3: evm.model.Contig35.331   evm.model.Contig5.109      1e-84
245-  4: evm.model.Contig35.336   evm.model.Contig5.114      6e-53
245-  5: evm.model.Contig35.337   evm.model.Contig5.116      6e-30
## Alignment 246: score=253.0 e_value=1e-09 N=6 ew35&ew5 plus
246-  0: evm.model.Contig35.500   evm.model.Contig5.368      3e-50
246-  1: evm.model.Contig35.503   evm.model.Contig5.379      4e-32
246-  2: evm.model.Contig35.505   evm.model.Contig5.383     2e-111
246-  3: evm.model.Contig35.512   evm.model.Contig5.388          0
246-  4: evm.model.Contig35.515   evm.model.Contig5.392          0
246-  5: evm.model.Contig35.524   evm.model.Contig5.418      3e-27
## Alignment 247: score=1360.0 e_value=1.5e-100 N=30 ew35&ew5 minus
247-  0: evm.model.Contig35.156   evm.model.Contig5.249          0
```

```
247-  1: evm.model.Contig35.164   evm.model.Contig5.246     2e-175
247-  2: evm.model.Contig35.169   evm.model.Contig5.245      4e-41
247-  3: evm.model.Contig35.184   evm.model.Contig5.239      9e-47
247-  4: evm.model.Contig35.189   evm.model.Contig5.235          0
247-  5: evm.model.Contig35.191   evm.model.Contig5.229      8e-39
247-  6: evm.model.Contig35.193   evm.model.Contig5.227      4e-34
247-  7: evm.model.Contig35.196   evm.model.Contig5.222     2e-138
247-  8: evm.model.Contig35.197   evm.model.Contig5.221          0
247-  9: evm.model.Contig35.201   evm.model.Contig5.218      3e-85
247- 10: evm.model.Contig35.206   evm.model.Contig5.216     3e-103
247- 11: evm.model.Contig35.207   evm.model.Contig5.214     4e-178
247- 12: evm.model.Contig35.219   evm.model.Contig5.199      3e-87
247- 13: evm.model.Contig35.222   evm.model.Contig5.198      5e-90
247- 14: evm.model.Contig35.229   evm.model.Contig5.190          0
247- 15: evm.model.Contig35.233   evm.model.Contig5.187      4e-38
247- 16: evm.model.Contig35.247   evm.model.Contig5.171      9e-45
247- 17: evm.model.Contig35.250   evm.model.Contig5.169      9e-40
247- 18: evm.model.Contig35.274   evm.model.Contig5.153      9e-90
247- 19: evm.model.Contig35.277   evm.model.Contig5.152          0
247- 20: evm.model.Contig35.278   evm.model.Contig5.151      8e-58
247- 21: evm.model.Contig35.285   evm.model.Contig5.146      4e-96
247- 22: evm.model.Contig35.286   evm.model.Contig5.145      8e-39
247- 23: evm.model.Contig35.290   evm.model.Contig5.144     1e-104
247- 24: evm.model.Contig35.292   evm.model.Contig5.139     4e-162
247- 25: evm.model.Contig35.297   evm.model.Contig5.138      2e-75
247- 26: evm.model.Contig35.298   evm.model.Contig5.137      1e-57
247- 27: evm.model.Contig35.301   evm.model.Contig5.135      4e-30
247- 28: evm.model.Contig35.304   evm.model.Contig5.129          0
247- 29: evm.model.Contig35.306   evm.model.Contig5.127      8e-48
## Alignment 248: score=381.0 e_value=4.4e-19 N=8 ew35&ew5 minus
248-  0: evm.model.Contig35.108   evm.model.Contig5.510      3e-59
248-  1: evm.model.Contig35.109   evm.model.Contig5.502          0
248-  2: evm.model.Contig35.119   evm.model.Contig5.501          0
248-  3: evm.model.Contig35.120   evm.model.Contig5.498      5e-50
248-  4: evm.model.Contig35.121   evm.model.Contig5.497     7e-136
248-  5: evm.model.Contig35.122   evm.model.Contig5.496      1e-62
248-  6: evm.model.Contig35.123   evm.model.Contig5.495          0
248-  7: evm.model.Contig35.125   evm.model.Contig5.494          0
## Alignment 249: score=372.0 e_value=8.3e-17 N=8 ew35&ew5 minus
249-  0: evm.model.Contig35.398   evm.model.Contig5.320     7e-104
249-  1: evm.model.Contig35.416   evm.model.Contig5.306      2e-40
249-  2: evm.model.Contig35.418   evm.model.Contig5.305     2e-102
249-  3: evm.model.Contig35.428   evm.model.Contig5.304      4e-91
249-  4: evm.model.Contig35.429   evm.model.Contig5.303          0
249-  5: evm.model.Contig35.431   evm.model.Contig5.301      1e-24
249-  6: evm.model.Contig35.432   evm.model.Contig5.300     4e-105
249-  7: evm.model.Contig35.433   evm.model.Contig5.299          0
## Alignment 250: score=371.0 e_value=6e-14 N=8 ew35&ew5 minus
250-  0: evm.model.Contig35.326   evm.model.Contig5.100      9e-79
250-  1: evm.model.Contig35.329   evm.model.Contig5.99       9e-27
250-  2: evm.model.Contig35.331   evm.model.Contig5.97       2e-63
250-  3: evm.model.Contig35.334   evm.model.Contig5.93      1e-107
250-  4: evm.model.Contig35.336   evm.model.Contig5.91       6e-17
250-  5: evm.model.Contig35.343   evm.model.Contig5.89           0
```

```
250-  6: evm.model.Contig35.350   evm.model.Contig5.88      2e-149
250-  7: evm.model.Contig35.354   evm.model.Contig5.77      1e-135
## Alignment 251: score=314.0 e_value=1.9e-12 N=7 ew35&ew5 minus
251-  0: evm.model.Contig35.472   evm.model.Contig5.96       2e-23
251-  1: evm.model.Contig35.477   evm.model.Contig5.81           0
251-  2: evm.model.Contig35.482   evm.model.Contig5.69       8e-98
251-  3: evm.model.Contig35.488   evm.model.Contig5.65           0
251-  4: evm.model.Contig35.491   evm.model.Contig5.60       7e-34
251-  5: evm.model.Contig35.492   evm.model.Contig5.59      2e-161
251-  6: evm.model.Contig35.493   evm.model.Contig5.56           0
## Alignment 252: score=660.0 e_value=1.9e-39 N=14 ew36&ew36 minus
252-  0: evm.model.Contig36.457   evm.model.Contig36.541    1e-106
252-  1: evm.model.Contig36.471   evm.model.Contig36.539    1e-140
252-  2: evm.model.Contig36.472   evm.model.Contig36.538         0
252-  3: evm.model.Contig36.473   evm.model.Contig36.537         0
252-  4: evm.model.Contig36.474   evm.model.Contig36.536         0
252-  5: evm.model.Contig36.475   evm.model.Contig36.535         0
252-  6: evm.model.Contig36.476   evm.model.Contig36.534         0
252-  7: evm.model.Contig36.477   evm.model.Contig36.531         0
252-  8: evm.model.Contig36.481   evm.model.Contig36.530         0
252-  9: evm.model.Contig36.482   evm.model.Contig36.529         0
252- 10: evm.model.Contig36.483   evm.model.Contig36.528     1e-52
252- 11: evm.model.Contig36.485   evm.model.Contig36.525         0
252- 12: evm.model.Contig36.487   evm.model.Contig36.524         0
252- 13: evm.model.Contig36.503   evm.model.Contig36.504     2e-57
## Alignment 253: score=482.0 e_value=7.9e-24 N=10 ew36&ew36 minus
253-  0: evm.model.Contig36.8     evm.model.Contig36.134         0
253-  1: evm.model.Contig36.10    evm.model.Contig36.130     3e-74
253-  2: evm.model.Contig36.12    evm.model.Contig36.127    4e-160
253-  3: evm.model.Contig36.13    evm.model.Contig36.126     2e-76
253-  4: evm.model.Contig36.14    evm.model.Contig36.125     8e-36
253-  5: evm.model.Contig36.19    evm.model.Contig36.120     8e-85
253-  6: evm.model.Contig36.22    evm.model.Contig36.111    3e-164
253-  7: evm.model.Contig36.24    evm.model.Contig36.110         0
253-  8: evm.model.Contig36.25    evm.model.Contig36.109    9e-154
253-  9: evm.model.Contig36.26    evm.model.Contig36.108    4e-152
## Alignment 254: score=252.0 e_value=1.3e-08 N=6 ew37&ew37 plus
254-  0: evm.model.Contig37.280   evm.model.Contig37.340     7e-40
254-  1: evm.model.Contig37.291   evm.model.Contig37.344     4e-36
254-  2: evm.model.Contig37.296   evm.model.Contig37.348         0
254-  3: evm.model.Contig37.299   evm.model.Contig37.351    3e-127
254-  4: evm.model.Contig37.307   evm.model.Contig37.354         0
254-  5: evm.model.Contig37.333   evm.model.Contig37.380         0
## Alignment 255: score=430.0 e_value=3.8e-16 N=9 ew37&ew37 minus
255-  0: evm.model.Contig37.395   evm.model.Contig37.445     2e-68
255-  1: evm.model.Contig37.397   evm.model.Contig37.443         0
255-  2: evm.model.Contig37.398   evm.model.Contig37.442         0
255-  3: evm.model.Contig37.400   evm.model.Contig37.438     2e-52
255-  4: evm.model.Contig37.401   evm.model.Contig37.432     2e-47
255-  5: evm.model.Contig37.402   evm.model.Contig37.429         0
255-  6: evm.model.Contig37.403   evm.model.Contig37.423         0
255-  7: evm.model.Contig37.404   evm.model.Contig37.419         0
255-  8: evm.model.Contig37.406   evm.model.Contig37.418    8e-122
## Alignment 256: score=317.0 e_value=9e-14 N=7 ew37&ew37 minus
```

```
256-  0: evm.model.Contig37.320   evm.model.Contig37.394     2e-17
256-  1: evm.model.Contig37.333   evm.model.Contig37.380         0
256-  2: evm.model.Contig37.335   evm.model.Contig37.379     1e-43
256-  3: evm.model.Contig37.337   evm.model.Contig37.378    5e-124
256-  4: evm.model.Contig37.338   evm.model.Contig37.377         0
256-  5: evm.model.Contig37.356   evm.model.Contig37.364         0
256-  6: evm.model.Contig37.358   evm.model.Contig37.362         0
## Alignment 257: score=478.0 e_value=7e-27 N=12 ew37&ew9 plus
257-  0: evm.model.Contig37.428   evm.model.Contig9.405     6e-106
257-  1: evm.model.Contig37.429   evm.model.Contig9.406          0
257-  2: evm.model.Contig37.434   evm.model.Contig9.431      5e-46
257-  3: evm.model.Contig37.435   evm.model.Contig9.432     1e-140
257-  4: evm.model.Contig37.445   evm.model.Contig9.435     5e-131
257-  5: evm.model.Contig37.453   evm.model.Contig9.448      4e-17
257-  6: evm.model.Contig37.458   evm.model.Contig9.455          0
257-  7: evm.model.Contig37.459   evm.model.Contig9.461          0
257-  8: evm.model.Contig37.461   evm.model.Contig9.477      8e-18
257-  9: evm.model.Contig37.471   evm.model.Contig9.503          0
257- 10: evm.model.Contig37.478   evm.model.Contig9.505     3e-120
257- 11: evm.model.Contig37.480   evm.model.Contig9.526          0
## Alignment 258: score=334.0 e_value=2.9e-11 N=7 ew37&ew9 plus
258-  0: evm.model.Contig37.97    evm.model.Contig9.59           0
258-  1: evm.model.Contig37.100   evm.model.Contig9.64           0
258-  2: evm.model.Contig37.102   evm.model.Contig9.65       3e-35
258-  3: evm.model.Contig37.104   evm.model.Contig9.66      7e-173
258-  4: evm.model.Contig37.108   evm.model.Contig9.67           0
258-  5: evm.model.Contig37.109   evm.model.Contig9.68           0
258-  6: evm.model.Contig37.112   evm.model.Contig9.76      7e-131
## Alignment 259: score=318.0 e_value=5.4e-13 N=7 ew37&ew9 plus
259-  0: evm.model.Contig37.13    evm.model.Contig9.612      1e-37
259-  1: evm.model.Contig37.26    evm.model.Contig9.619          0
259-  2: evm.model.Contig37.27    evm.model.Contig9.620      6e-44
259-  3: evm.model.Contig37.32    evm.model.Contig9.621          0
259-  4: evm.model.Contig37.48    evm.model.Contig9.625     6e-170
259-  5: evm.model.Contig37.50    evm.model.Contig9.627          0
259-  6: evm.model.Contig37.51    evm.model.Contig9.628          0
## Alignment 260: score=980.0 e_value=4.2e-65 N=22 ew37&ew9 minus
260-  0: evm.model.Contig37.32    evm.model.Contig9.621          0
260-  1: evm.model.Contig37.40    evm.model.Contig9.614      1e-99
260-  2: evm.model.Contig37.41    evm.model.Contig9.613      4e-46
260-  3: evm.model.Contig37.46    evm.model.Contig9.611          0
260-  4: evm.model.Contig37.54    evm.model.Contig9.607     2e-143
260-  5: evm.model.Contig37.58    evm.model.Contig9.596      2e-27
260-  6: evm.model.Contig37.60    evm.model.Contig9.593     6e-136
260-  7: evm.model.Contig37.61    evm.model.Contig9.589      1e-79
260-  8: evm.model.Contig37.63    evm.model.Contig9.582     5e-101
260-  9: evm.model.Contig37.64    evm.model.Contig9.581     6e-179
260- 10: evm.model.Contig37.68    evm.model.Contig9.579     1e-130
260- 11: evm.model.Contig37.69    evm.model.Contig9.574     2e-127
260- 12: evm.model.Contig37.71    evm.model.Contig9.570      6e-77
260- 13: evm.model.Contig37.75    evm.model.Contig9.559      3e-30
260- 14: evm.model.Contig37.76    evm.model.Contig9.550     4e-118
260- 15: evm.model.Contig37.78    evm.model.Contig9.544      3e-77
260- 16: evm.model.Contig37.79    evm.model.Contig9.535          0
```

```
260- 17: evm.model.Contig37.80    evm.model.Contig9.534      4e-31
260- 18: evm.model.Contig37.82    evm.model.Contig9.533     1e-100
260- 19: evm.model.Contig37.89    evm.model.Contig9.529          0
260- 20: evm.model.Contig37.90    evm.model.Contig9.514      3e-49
260- 21: evm.model.Contig37.94    evm.model.Contig9.494     1e-110
## Alignment 261: score=739.0 e_value=8e-52 N=17 ew37&ew9 minus
261-  0: evm.model.Contig37.297   evm.model.Contig9.231      6e-23
261-  1: evm.model.Contig37.311   evm.model.Contig9.218      4e-24
261-  2: evm.model.Contig37.317   evm.model.Contig9.211      1e-62
261-  3: evm.model.Contig37.335   evm.model.Contig9.208      2e-89
261-  4: evm.model.Contig37.358   evm.model.Contig9.184      2e-46
261-  5: evm.model.Contig37.365   evm.model.Contig9.182      1e-87
261-  6: evm.model.Contig37.368   evm.model.Contig9.177     1e-173
261-  7: evm.model.Contig37.375   evm.model.Contig9.176      3e-83
261-  8: evm.model.Contig37.379   evm.model.Contig9.171      8e-26
261-  9: evm.model.Contig37.382   evm.model.Contig9.149          0
261- 10: evm.model.Contig37.384   evm.model.Contig9.146          0
261- 11: evm.model.Contig37.385   evm.model.Contig9.145     3e-121
261- 12: evm.model.Contig37.386   evm.model.Contig9.143          0
261- 13: evm.model.Contig37.387   evm.model.Contig9.142          0
261- 14: evm.model.Contig37.389   evm.model.Contig9.141      1e-41
261- 15: evm.model.Contig37.390   evm.model.Contig9.140          0
261- 16: evm.model.Contig37.392   evm.model.Contig9.132      9e-19
## Alignment 262: score=260.0 e_value=1.3e-11 N=7 ew37&ew9 minus
262-  0: evm.model.Contig37.27    evm.model.Contig9.620      6e-44
262-  1: evm.model.Contig37.39    evm.model.Contig9.612      1e-96
262-  2: evm.model.Contig37.58    evm.model.Contig9.604      3e-73
262-  3: evm.model.Contig37.70    evm.model.Contig9.578      2e-62
262-  4: evm.model.Contig37.78    evm.model.Contig9.562      2e-70
262-  5: evm.model.Contig37.79    evm.model.Contig9.543          0
262-  6: evm.model.Contig37.82    evm.model.Contig9.539     5e-131
## Alignment 263: score=257.0 e_value=3.2e-08 N=6 ew37&ew9 minus
263-  0: evm.model.Contig37.463   evm.model.Contig9.522          0
263-  1: evm.model.Contig37.469   evm.model.Contig9.516          0
263-  2: evm.model.Contig37.479   evm.model.Contig9.503          0
263-  3: evm.model.Contig37.487   evm.model.Contig9.501          0
263-  4: evm.model.Contig37.488   evm.model.Contig9.484     7e-164
263-  5: evm.model.Contig37.489   evm.model.Contig9.480          0
## Alignment 264: score=615.0 e_value=1.4e-36 N=15 ew38&ew39 plus
264-  0: evm.model.Contig38.245   evm.model.Contig39.265    1e-149
264-  1: evm.model.Contig38.253   evm.model.Contig39.276     3e-12
264-  2: evm.model.Contig38.256   evm.model.Contig39.285     1e-94
264-  3: evm.model.Contig38.278   evm.model.Contig39.310     2e-26
264-  4: evm.model.Contig38.290   evm.model.Contig39.316    2e-121
264-  5: evm.model.Contig38.292   evm.model.Contig39.318     4e-15
264-  6: evm.model.Contig38.295   evm.model.Contig39.338     3e-76
264-  7: evm.model.Contig38.297   evm.model.Contig39.345     3e-76
264-  8: evm.model.Contig38.301   evm.model.Contig39.349         0
264-  9: evm.model.Contig38.303   evm.model.Contig39.355    1e-105
264- 10: evm.model.Contig38.306   evm.model.Contig39.358     4e-47
264- 11: evm.model.Contig38.313   evm.model.Contig39.375     1e-96
264- 12: evm.model.Contig38.319   evm.model.Contig39.377     7e-58
264- 13: evm.model.Contig38.331   evm.model.Contig39.382     3e-22
264- 14: evm.model.Contig38.334   evm.model.Contig39.397    4e-168
```

```
## Alignment 265: score=286.0 e_value=9.3e-13 N=7 ew38&ew39 plus
265-  0: evm.model.Contig38.51    evm.model.Contig39.183    3e-115
265-  1: evm.model.Contig38.58    evm.model.Contig39.190    2e-130
265-  2: evm.model.Contig38.59    evm.model.Contig39.191     8e-90
265-  3: evm.model.Contig38.65    evm.model.Contig39.195     2e-32
265-  4: evm.model.Contig38.87    evm.model.Contig39.199     1e-94
265-  5: evm.model.Contig38.105   evm.model.Contig39.224     3e-77
265-  6: evm.model.Contig38.114   evm.model.Contig39.226         0
## Alignment 266: score=447.0 e_value=1e-22 N=11 ew38&ew39 minus
266-  0: evm.model.Contig38.29    evm.model.Contig39.206     2e-68
266-  1: evm.model.Contig38.46    evm.model.Contig39.199    1e-144
266-  2: evm.model.Contig38.59    evm.model.Contig39.198     2e-90
266-  3: evm.model.Contig38.65    evm.model.Contig39.195     2e-32
266-  4: evm.model.Contig38.88    evm.model.Contig39.185     2e-60
266-  5: evm.model.Contig38.96    evm.model.Contig39.179         0
266-  6: evm.model.Contig38.97    evm.model.Contig39.173     5e-11
266-  7: evm.model.Contig38.99    evm.model.Contig39.172    7e-166
266-  8: evm.model.Contig38.107   evm.model.Contig39.169    5e-131
266-  9: evm.model.Contig38.129   evm.model.Contig39.156     7e-88
266- 10: evm.model.Contig38.137   evm.model.Contig39.150    7e-176
## Alignment 267: score=413.0 e_value=8.5e-18 N=9 ew38&ew39 minus
267-  0: evm.model.Contig38.236   evm.model.Contig39.293     1e-29
267-  1: evm.model.Contig38.243   evm.model.Contig39.290         0
267-  2: evm.model.Contig38.244   evm.model.Contig39.287         0
267-  3: evm.model.Contig38.250   evm.model.Contig39.284    3e-156
267-  4: evm.model.Contig38.251   evm.model.Contig39.283    1e-117
267-  5: evm.model.Contig38.258   evm.model.Contig39.273     1e-50
267-  6: evm.model.Contig38.261   evm.model.Contig39.269    2e-134
267-  7: evm.model.Contig38.264   evm.model.Contig39.266    8e-103
267-  8: evm.model.Contig38.275   evm.model.Contig39.256    1e-126
## Alignment 268: score=407.0 e_value=6.3e-19 N=9 ew38&ew39 minus
268-  0: evm.model.Contig38.160   evm.model.Contig39.431    2e-157
268-  1: evm.model.Contig38.167   evm.model.Contig39.425     2e-57
268-  2: evm.model.Contig38.172   evm.model.Contig39.423         0
268-  3: evm.model.Contig38.178   evm.model.Contig39.419         0
268-  4: evm.model.Contig38.179   evm.model.Contig39.418     1e-31
268-  5: evm.model.Contig38.183   evm.model.Contig39.416     3e-88
268-  6: evm.model.Contig38.188   evm.model.Contig39.412     3e-60
268-  7: evm.model.Contig38.190   evm.model.Contig39.410    4e-120
268-  8: evm.model.Contig38.211   evm.model.Contig39.409     4e-79
## Alignment 269: score=290.0 e_value=9.6e-09 N=6 ew39&ew39 plus
269-  0: evm.model.Contig39.1     evm.model.Contig39.34      1e-86
269-  1: evm.model.Contig39.3     evm.model.Contig39.44      8e-79
269-  2: evm.model.Contig39.4     evm.model.Contig39.45      8e-90
269-  3: evm.model.Contig39.5     evm.model.Contig39.47     7e-171
269-  4: evm.model.Contig39.6     evm.model.Contig39.48      3e-46
269-  5: evm.model.Contig39.7     evm.model.Contig39.49          0
## Alignment 270: score=407.0 e_value=5.7e-18 N=9 ew4&ew4 minus
270-  0: evm.model.Contig4.399    evm.model.Contig4.472          0
270-  1: evm.model.Contig4.401    evm.model.Contig4.471          0
270-  2: evm.model.Contig4.405    evm.model.Contig4.470          0
270-  3: evm.model.Contig4.408    evm.model.Contig4.469     2e-158
270-  4: evm.model.Contig4.410    evm.model.Contig4.468      2e-88
270-  5: evm.model.Contig4.413    evm.model.Contig4.466      2e-78
```

```
270-  6: evm.model.Contig4.435    evm.model.Contig4.459     1e-143
270-  7: evm.model.Contig4.442    evm.model.Contig4.457      2e-69
270-  8: evm.model.Contig4.450    evm.model.Contig4.451          0
## Alignment 271: score=290.0 e_value=1.6e-16 N=7 ew4&ew4 minus
271-  0: evm.model.Contig4.54     evm.model.Contig4.133      3e-82
271-  1: evm.model.Contig4.67     evm.model.Contig4.132      4e-59
271-  2: evm.model.Contig4.68     evm.model.Contig4.117      2e-39
271-  3: evm.model.Contig4.69     evm.model.Contig4.95      3e-177
271-  4: evm.model.Contig4.74     evm.model.Contig4.91       1e-12
271-  5: evm.model.Contig4.83     evm.model.Contig4.90       7e-28
271-  6: evm.model.Contig4.85     evm.model.Contig4.89           0
## Alignment 272: score=278.0 e_value=1e-09 N=6 ew4&ew4 minus
272-  0: evm.model.Contig4.190    evm.model.Contig4.248          0
272-  1: evm.model.Contig4.204    evm.model.Contig4.236          0
272-  2: evm.model.Contig4.206    evm.model.Contig4.235      7e-96
272-  3: evm.model.Contig4.207    evm.model.Contig4.234          0
272-  4: evm.model.Contig4.212    evm.model.Contig4.228          0
272-  5: evm.model.Contig4.216    evm.model.Contig4.224      2e-77
## Alignment 273: score=348.0 e_value=6.4e-11 N=7 ew40&ew40 minus
273-  0: evm.model.Contig40.67    evm.model.Contig40.100         0
273-  1: evm.model.Contig40.68    evm.model.Contig40.99          0
273-  2: evm.model.Contig40.69    evm.model.Contig40.97          0
273-  3: evm.model.Contig40.70    evm.model.Contig40.96          0
273-  4: evm.model.Contig40.72    evm.model.Contig40.95          0
273-  5: evm.model.Contig40.73    evm.model.Contig40.94      3e-32
273-  6: evm.model.Contig40.74    evm.model.Contig40.93      5e-76
## Alignment 274: score=423.0 e_value=9.1e-19 N=9 ew41&ew41 plus
274-  0: evm.model.Contig41.12    evm.model.Contig41.90          0
274-  1: evm.model.Contig41.31    evm.model.Contig41.97          0
274-  2: evm.model.Contig41.33    evm.model.Contig41.99          0
274-  3: evm.model.Contig41.35    evm.model.Contig41.102     2e-83
274-  4: evm.model.Contig41.36    evm.model.Contig41.103         0
274-  5: evm.model.Contig41.38    evm.model.Contig41.104         0
274-  6: evm.model.Contig41.40    evm.model.Contig41.108    7e-180
274-  7: evm.model.Contig41.41    evm.model.Contig41.109         0
274-  8: evm.model.Contig41.44    evm.model.Contig41.111         0
## Alignment 275: score=274.0 e_value=4.5e-09 N=6 ew5&ew5 plus
275-  0: evm.model.Contig5.609    evm.model.Contig5.650      6e-72
275-  1: evm.model.Contig5.612    evm.model.Contig5.659      6e-93
275-  2: evm.model.Contig5.613    evm.model.Contig5.661          0
275-  3: evm.model.Contig5.617    evm.model.Contig5.674          0
275-  4: evm.model.Contig5.619    evm.model.Contig5.677     4e-173
275-  5: evm.model.Contig5.623    evm.model.Contig5.678          0
## Alignment 276: score=1332.0 e_value=1.2e-91 N=28 ew5&ew5 minus
276-  0: evm.model.Contig5.673    evm.model.Contig5.1032     4e-78
276-  1: evm.model.Contig5.692    evm.model.Contig5.1027     3e-36
276-  2: evm.model.Contig5.696    evm.model.Contig5.1026         0
276-  3: evm.model.Contig5.700    evm.model.Contig5.1024     6e-66
276-  4: evm.model.Contig5.704    evm.model.Contig5.1022    1e-105
276-  5: evm.model.Contig5.705    evm.model.Contig5.1020     3e-41
276-  6: evm.model.Contig5.706    evm.model.Contig5.1019         0
276-  7: evm.model.Contig5.707    evm.model.Contig5.1018    2e-114
276-  8: evm.model.Contig5.711    evm.model.Contig5.1017         0
276-  9: evm.model.Contig5.714    evm.model.Contig5.1015         0
```

```
276- 10: evm.model.Contig5.715    evm.model.Contig5.1014         0
276- 11: evm.model.Contig5.716    evm.model.Contig5.1013         0
276- 12: evm.model.Contig5.717    evm.model.Contig5.1011         0
276- 13: evm.model.Contig5.718    evm.model.Contig5.1010    1e-180
276- 14: evm.model.Contig5.722    evm.model.Contig5.1009         0
276- 15: evm.model.Contig5.723    evm.model.Contig5.1007         0
276- 16: evm.model.Contig5.725    evm.model.Contig5.1006     1e-64
276- 17: evm.model.Contig5.727    evm.model.Contig5.1004         0
276- 18: evm.model.Contig5.730    evm.model.Contig5.1001         0
276- 19: evm.model.Contig5.734    evm.model.Contig5.1000         0
276- 20: evm.model.Contig5.741    evm.model.Contig5.999      1e-22
276- 21: evm.model.Contig5.746    evm.model.Contig5.998          0
276- 22: evm.model.Contig5.747    evm.model.Contig5.997          0
276- 23: evm.model.Contig5.758    evm.model.Contig5.996     9e-108
276- 24: evm.model.Contig5.759    evm.model.Contig5.995      2e-61
276- 25: evm.model.Contig5.762    evm.model.Contig5.994          0
276- 26: evm.model.Contig5.763    evm.model.Contig5.993          0
276- 27: evm.model.Contig5.765    evm.model.Contig5.992     4e-116
## Alignment 277: score=389.0 e_value=3.7e-14 N=8 ew5&ew5 minus
277-  0: evm.model.Contig5.87     evm.model.Contig5.119          0
277-  1: evm.model.Contig5.90     evm.model.Contig5.117          0
277-  2: evm.model.Contig5.91     evm.model.Contig5.113          0
277-  3: evm.model.Contig5.92     evm.model.Contig5.112          0
277-  4: evm.model.Contig5.96     evm.model.Contig5.111     3e-143
277-  5: evm.model.Contig5.97     evm.model.Contig5.109          0
277-  6: evm.model.Contig5.99     evm.model.Contig5.108     1e-150
277-  7: evm.model.Contig5.100    evm.model.Contig5.106     1e-127
## Alignment 278: score=346.0 e_value=2.3e-12 N=7 ew5&ew5 minus
278-  0: evm.model.Contig5.276    evm.model.Contig5.303          0
278-  1: evm.model.Contig5.278    evm.model.Contig5.302          0
278-  2: evm.model.Contig5.279    evm.model.Contig5.301      9e-46
278-  3: evm.model.Contig5.280    evm.model.Contig5.300          0
278-  4: evm.model.Contig5.281    evm.model.Contig5.299          0
278-  5: evm.model.Contig5.283    evm.model.Contig5.298          0
278-  6: evm.model.Contig5.286    evm.model.Contig5.295     5e-110
## Alignment 279: score=328.0 e_value=1.8e-13 N=7 ew5&ew5 minus
279-  0: evm.model.Contig5.903    evm.model.Contig5.991      8e-74
279-  1: evm.model.Contig5.916    evm.model.Contig5.984      2e-28
279-  2: evm.model.Contig5.917    evm.model.Contig5.982      4e-29
279-  3: evm.model.Contig5.919    evm.model.Contig5.981      9e-36
279-  4: evm.model.Contig5.925    evm.model.Contig5.980          0
279-  5: evm.model.Contig5.926    evm.model.Contig5.978          0
279-  6: evm.model.Contig5.929    evm.model.Contig5.977          0
## Alignment 280: score=291.0 e_value=1.7e-09 N=6 ew5&ew5 minus
280-  0: evm.model.Contig5.849    evm.model.Contig5.881          0
280-  1: evm.model.Contig5.850    evm.model.Contig5.880     1e-101
280-  2: evm.model.Contig5.854    evm.model.Contig5.879     6e-115
280-  3: evm.model.Contig5.861    evm.model.Contig5.872          0
280-  4: evm.model.Contig5.862    evm.model.Contig5.871          0
280-  5: evm.model.Contig5.863    evm.model.Contig5.870      2e-63
## Alignment 281: score=317.0 e_value=9.6e-13 N=7 ew6&ew6 plus
281-  0: evm.model.Contig6.932    evm.model.Contig6.1007    1e-128
281-  1: evm.model.Contig6.956    evm.model.Contig6.1008    6e-155
281-  2: evm.model.Contig6.967    evm.model.Contig6.1009         0
```

```
281-  3: evm.model.Contig6.968    evm.model.Contig6.1010        0
281-  4: evm.model.Contig6.969    evm.model.Contig6.1011        0
281-  5: evm.model.Contig6.970    evm.model.Contig6.1012        0
281-  6: evm.model.Contig6.971    evm.model.Contig6.1013    9e-94
## Alignment 282: score=632.0 e_value=7.3e-30 N=13 ew6&ew6 minus
282-  0: evm.model.Contig6.724    evm.model.Contig6.779     6e-89
282-  1: evm.model.Contig6.725    evm.model.Contig6.778    5e-138
282-  2: evm.model.Contig6.726    evm.model.Contig6.777         0
282-  3: evm.model.Contig6.727    evm.model.Contig6.776         0
282-  4: evm.model.Contig6.728    evm.model.Contig6.775         0
282-  5: evm.model.Contig6.729    evm.model.Contig6.769    2e-145
282-  6: evm.model.Contig6.730    evm.model.Contig6.768         0
282-  7: evm.model.Contig6.731    evm.model.Contig6.767     1e-75
282-  8: evm.model.Contig6.732    evm.model.Contig6.766         0
282-  9: evm.model.Contig6.733    evm.model.Contig6.761         0
282- 10: evm.model.Contig6.734    evm.model.Contig6.760         0
282- 11: evm.model.Contig6.739    evm.model.Contig6.759         0
282- 12: evm.model.Contig6.743    evm.model.Contig6.753     9e-75
## Alignment 283: score=254.0 e_value=6.6e-11 N=6 ew6&ew6 minus
283-  0: evm.model.Contig6.934    evm.model.Contig6.1025        0
283-  1: evm.model.Contig6.948    evm.model.Contig6.1018    5e-31
283-  2: evm.model.Contig6.956    evm.model.Contig6.1008   6e-155
283-  3: evm.model.Contig6.974    evm.model.Contig6.985         0
283-  4: evm.model.Contig6.975    evm.model.Contig6.984         0
283-  5: evm.model.Contig6.978    evm.model.Contig6.983    2e-146
## Alignment 284: score=253.0 e_value=1.4e-13 N=6 ew6&ew6 minus
284-  0: evm.model.Contig6.828    evm.model.Contig6.1057        0
284-  1: evm.model.Contig6.852    evm.model.Contig6.1047        0
284-  2: evm.model.Contig6.872    evm.model.Contig6.1023        0
284-  3: evm.model.Contig6.873    evm.model.Contig6.1021    5e-66
284-  4: evm.model.Contig6.874    evm.model.Contig6.1020        0
284-  5: evm.model.Contig6.875    evm.model.Contig6.1019        0
## Alignment 285: score=283.0 e_value=1.4e-13 N=6 ew7&ew7 minus
285-  0: evm.model.Contig7.60     evm.model.Contig7.105    1e-118
285-  1: evm.model.Contig7.75     evm.model.Contig7.89      3e-98
285-  2: evm.model.Contig7.76     evm.model.Contig7.86          0
285-  3: evm.model.Contig7.77     evm.model.Contig7.85          0
285-  4: evm.model.Contig7.78     evm.model.Contig7.84      3e-30
285-  5: evm.model.Contig7.79     evm.model.Contig7.83          0
## Alignment 286: score=252.0 e_value=1.9e-11 N=6 ew7&ew7 minus
286-  0: evm.model.Contig7.413    evm.model.Contig7.488     6e-83
286-  1: evm.model.Contig7.420    evm.model.Contig7.462         0
286-  2: evm.model.Contig7.426    evm.model.Contig7.450         0
286-  3: evm.model.Contig7.427    evm.model.Contig7.449     1e-41
286-  4: evm.model.Contig7.436    evm.model.Contig7.445         0
286-  5: evm.model.Contig7.441    evm.model.Contig7.444     6e-47
## Alignment 287: score=351.0 e_value=6.1e-17 N=8 ew8&ew8 plus
287-  0: evm.model.Contig8.9      evm.model.Contig8.457     6e-41
287-  1: evm.model.Contig8.26     evm.model.Contig8.466     2e-28
287-  2: evm.model.Contig8.30     evm.model.Contig8.467     2e-60
287-  3: evm.model.Contig8.38     evm.model.Contig8.469     1e-73
287-  4: evm.model.Contig8.57     evm.model.Contig8.486    7e-123
287-  5: evm.model.Contig8.59     evm.model.Contig8.488         0
287-  6: evm.model.Contig8.64     evm.model.Contig8.489    3e-143
```

```
287-  7: evm.model.Contig8.65     evm.model.Contig8.490          0
## Alignment 288: score=302.0 e_value=8.3e-13 N=7 ew9&ew9 plus
288-  0: evm.model.Contig9.264    evm.model.Contig9.323      2e-21
288-  1: evm.model.Contig9.285    evm.model.Contig9.340      2e-40
288-  2: evm.model.Contig9.289    evm.model.Contig9.346     6e-159
288-  3: evm.model.Contig9.290    evm.model.Contig9.347      3e-64
288-  4: evm.model.Contig9.295    evm.model.Contig9.349      4e-73
288-  5: evm.model.Contig9.301    evm.model.Contig9.354      1e-61
288-  6: evm.model.Contig9.316    evm.model.Contig9.362      4e-61
## Alignment 289: score=258.0 e_value=2e-10 N=6 ew9&ew9 plus
289-  0: evm.model.Contig9.202    evm.model.Contig9.281     9e-139
289-  1: evm.model.Contig9.203    evm.model.Contig9.286          0
289-  2: evm.model.Contig9.217    evm.model.Contig9.302      1e-62
289-  3: evm.model.Contig9.218    evm.model.Contig9.316      1e-76
289-  4: evm.model.Contig9.221    evm.model.Contig9.317      2e-62
289-  5: evm.model.Contig9.230    evm.model.Contig9.325          0
```
